# A metal π-Lewis base activation model for Pd-catalyzed hydroamination of amines and 1,3-dienes†

**DOI:** 10.1039/d2sc05835a

**Published:** 2023-04-03

**Authors:** Xiao Yan, Xiu-Ming Yang, Peng Yan, Bo Zhao, Rong Zeng, Bin Pan, Ying-Chun Chen, Lei Zhu, Qin Ouyang

**Affiliations:** a College of Pharmacy, Third Military Medical University Shapingba Chongqing 400038 China zhulei@tmmu.edu.cn ouyangq@tmmu.edu.cn; b Key Laboratory of Drug-Targeting and Drug Delivery System of the Ministry of Education, Sichuan Research Center for Drug Precision Industrial Technology, West China School of Pharmacy, Sichuan University Chengdu 610041 China

## Abstract

As a general mechanism proposal, a Pd(ii)–H migration insertion process is not able to well explicate the Pd-catalyzed hydroamination of amines and 1,3-dienes. Here we demonstrate that 1,3-dienes form electron-neutral and HOMO-raised η^2^-complexes with Pd(0) *via* π-Lewis base activation, which undergoes protonation with a variety of acidic sources, such as Brønsted acids, Lewis acid-activated indazoles, and Pd(ii) pre-catalyst triggered ammonium salts. The resultant π-allyl palladium complexes undergo the amination reaction to give the final observed products. FMO and NPA analyses have revealed the nature of Pd(0) mediated π-Lewis base activation of 1,3-dienes. The calculation results show that the π-Lewis base activation pathway is more favourable than the Pd(ii)–H species involved one in different reactions. Further control experiments corroborated our mechanistic proposal, and an efficient Pd(0) mediated hydroamination reaction was developed.

## Abstract 2

We propose a metal π-Lewis base activation model for Pd-catalyzed hydroamination of amines and 1,3-dienes, wherein the electron-rich Pd(0) was considered as a Lewis base to activate dienes *via* π-back donation.

## Graphical abstract

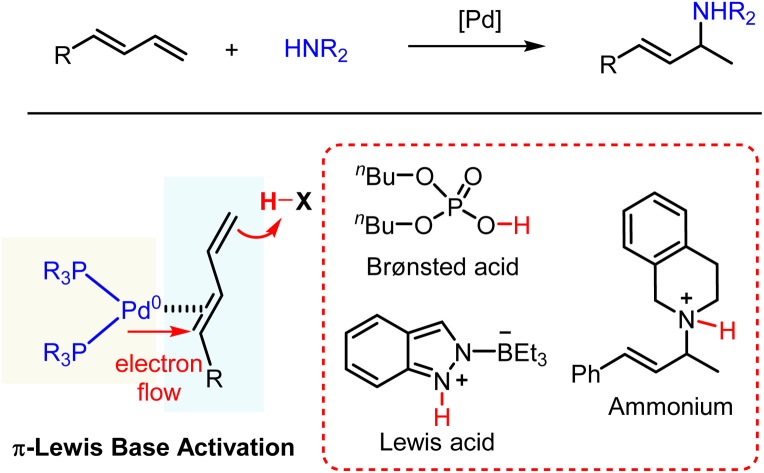

## Introduction

The hydroamination reaction of 1,3-dienes provides an efficient protocol to access densely functionalized chemicals which are widely applied in natural product synthesis, medicinal chemistry, and materials science.^1^ Over the past few decades, transition metal catalysis has been extensively explored in this field owing to its success in controllable selectivity and extensive product scope.^2^ In particular, Pd catalysis has received broad attention because of its high efficiency, mild conditions, and remarkable enantiocontrol.^3^

**Scheme 1:**
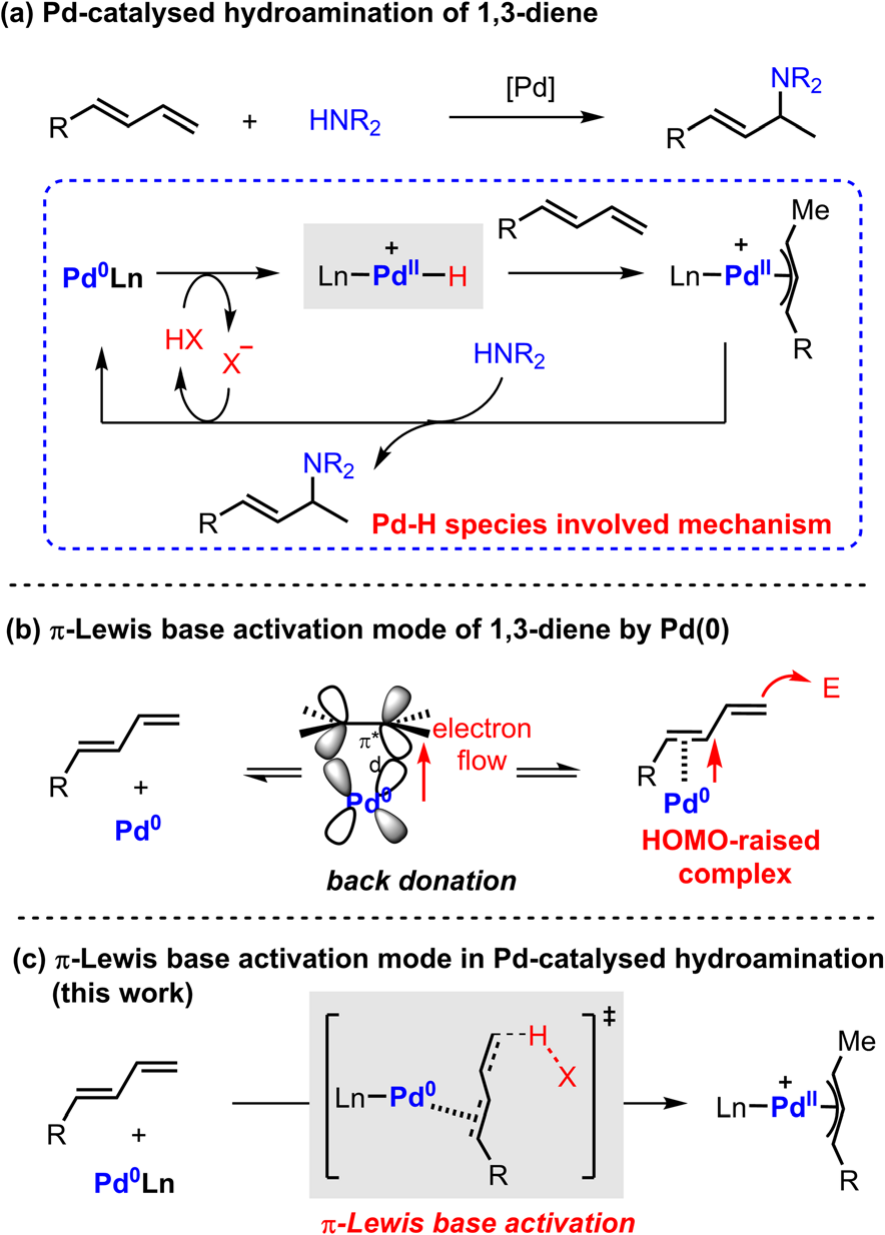
Pd(0) catalyzed hydroamination of 1,3-dienes and proposed mechanism.

The general proposed mechanism of Pd-catalyzed hydroamination of 1,3-dienes is described in Scheme 1a.^4^ The oxidative protonation of Pd(0) with an ancillary Brønsted acid generates Pd(ii)–H species, from which migratory insertion affords the key π-allyl palladium complex. The outer sphere attack of amine results in C–N bond formation and regenerates the Pd(0) catalyst. The Pd(ii)–H species are generally regarded as the crucial intermediates in the catalytic cycle. However, the Pd(ii)–H species have not been directly observed yet by experimental findings due to their thermodynamic instability. Theoretical investigation also suggested that the formation of Pd(ii)–H species would be kinetically infeasible under the reported catalytic conditions.^5^ Indeed, these observations cast a shadow on the mechanism study of Pd-catalyzed hydroamination of 1,3-dienes.

Alternatively, Pd(0) can directly activate 1,3-dienes through back donation.^6^ Our previous work indicated that the highest occupied molecular orbital (HOMO) energy of a η^2^-coordinated Pd(0)-diene complex is significantly raised compared with the parent one.^7^ It directly enhances the nucleophilicity of 1,3-dienes to attack electrophilic partners in a Friedel–Crafts reaction pattern (Scheme 1b). As a result, we speculated that a π-Lewis base activation mode might be similarly involved in the reported Pd-catalyzed hydroamination of 1,3-dienes (Scheme 1c). In this catalytic mode, Pd(0) would coordinate with 1,3-dienes and raise the electron density of unsaturated bonds, which would accelerate the protonation process with diverse acidic sources in a vinylogous manner to generate the key π-allyl palladium complexes.

**Scheme 2:**
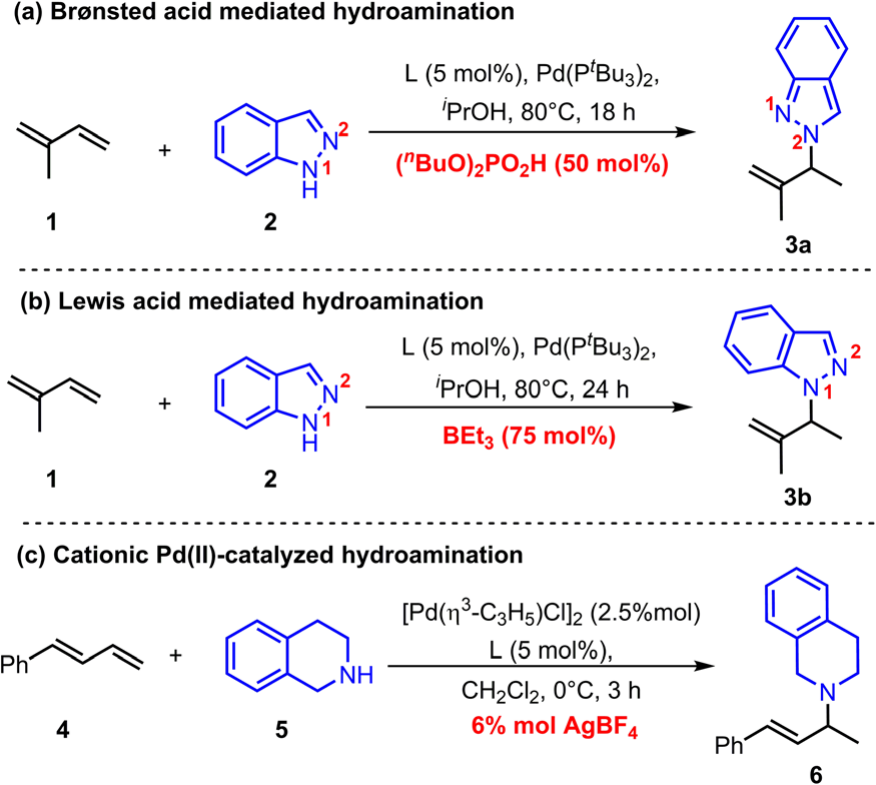
Representative Pd-catalyzed hydroamination of 1,3-diene.

Recently, Chen and co-workers reported a Pd(0)-catalyzed regiodivergent hydroamination of isoprene and indazoles,^8^ which could give the N^2^-functionalized product 3a with the assistance of Brønsted acid (^*n*^BuO)_2_PO_2_H (Scheme 2a). Instead, Lewis acid BEt_3_ could facilitate this reaction to produce N^1^-functionalized product 3b (Scheme 2b). Moreover, Malcolmson and co-workers demonstrated that the hydroamination of aliphatic amines and 1,3-dienes could be catalyzed by the cationic Pd(ii)-π-allyl catalyst without additional acid (Scheme 2c).^9^ In addition, the more accessible neutral [Pd(π-allyl)Cl]_2_ catalyst was also sufficient to mediate similar hydroamination reactions.^4*b*,10^ These results show that the Pd-catalyzed hydroamination of dienes could occur whether there is a Brønsted acid or not, which implies that there is an alternative activation model beyond the Pd(ii)–H migration insertion process. Herein, we reported a mechanism study for Pd-catalyzed hydroamination of 1,3-dienes and found that the π-Lewis base activation model is more favourable than the Pd(ii)–H migration insertion process. In this mechanism, Pd(0) complex activated 1,3-dienes could react with different kinds of electrophilic partners (acids) including phosphoric acid, Lewis acid-activated indazoles, and Pd(ii) pre-catalyst triggered ammonium salts. Importantly, according to these results, we updated the previously reported [Pd(π-allyl)Cl]_2_ catalyzed hydroamination of 1,3-dienes with pyrazole, and obtained an increased yield with higher efficiency by using catalytic amounts of Pd(0) and pyrazole hydrochloride.

## Computational methods

In this work, all of the density functional theory (DFT) calculations were performed by using Gaussian 09 ^11^ software packages. The B3LYP^12^ function together with the 6-31G(d)^13^ basis set (SDD^14^ basis set for Pd) was used for the geometry optimization and frequency analysis. Vibrational frequency calculations were performed for all the stationary points to confirm if each optimized structure is a local minimum or a transition state structure, as well as deriving the thermochemical corrections for the enthalpies and free energies. The intrinsic reaction coordinate (IRC) path was performed to check the energy profiles connecting each transition state to two associated minima of the proposed intermediates.^15^ After optimization, the B3LYP-D3 ^16^ function with the 6-311++G(d,p) basis set (SDD basis set for Pd) was used to calculate the single-point energies to give more accurate energy information. Besides, the solvent effect was considered by single-point calculations at the gas-phase stationary points with the SMD solvation model.^17^ When obtaining the relative Gibbs energy at 298 K, a correction of −2.6 (or 2.6) kcal mol^−1^ was performed for the transformation involving two molecules to one molecule (or one molecule to two molecules), to reduce the overestimation of entropy contribution.^18^ Optimized structures were illustrated by using CYLview.^19^

## Results and discussion

**Fig. 1:**
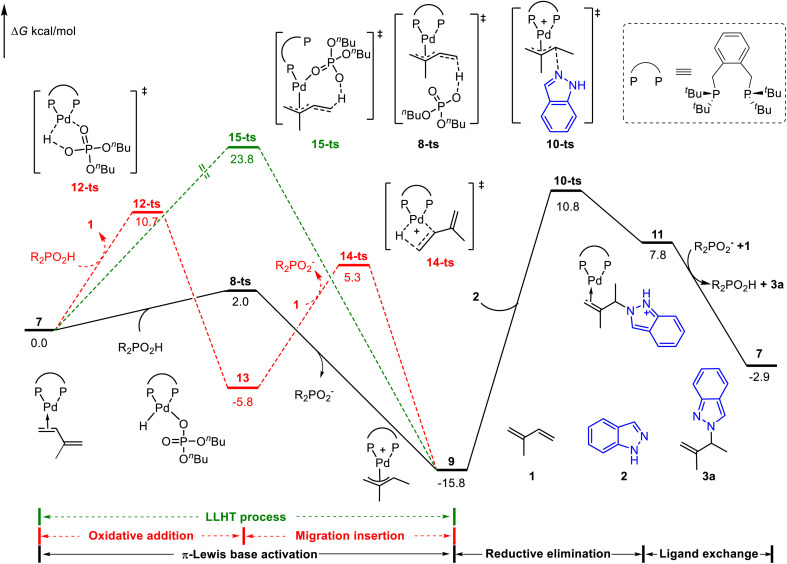
Reaction energy profile of the phosphoric acid-mediated Pd-catalyzed hydroamination of indazoles and isoprene.

We first considered the mechanism of Brønsted acid (^*n*^BuO)_2_PO_2_H mediated Pd(0)-catalyzed hydroamination of isoprene.^8^ The whole catalytic cycle could be divided into two parts: (1) the generation of the Pd(ii)-π-allyl complex; (2) outer sphere reductive elimination guided C–N bond formation. As shown in Fig. 1, the catalytic cycle starts from the isoprene coordinated Pd(0) complex 7. The Pd(ii)–H species involved oxidative addition/migration insertion path was illustrated in the red line with a corresponding energy barrier of 10.7 kcal mol^−1^ (*via*12-ts). On the other side, the π-Lewis base activation pathway proceeds *via*8-ts to afford Pd(ii)-π-allyl complex 9 directly (black line). In contrast, the corresponding energy barrier is only 2.0 kcal mol^−1^, which is 8.7 kcal mol^−1^ lower than that of the Pd(ii)–H species involved pathway. Moreover, the ligand-to-ligand hydrogen transfer (LLHT) process was also considered (green line),^5*d*,20^ and the corresponding energy barrier is up to 23.8 kcal mol^−1^ (*via*15-ts). These results indicated that π-Lewis base activation is energetically favourable in this Brønsted acid mediated Pd(0)-catalyzed hydroamination of dienes.

When the Pd(ii)-π-allyl complex 9 is formed, the subsequent outer sphere reductive elimination results in the C–N bond formation *via*10-ts with an energy barrier of 26.6 kcal mol^−1^ in the presence of indazoles. Product 3a was released after the subsequent ligand exchange, in which the active intermediate 7 was concomitantly regenerated. Calculated results showed that reductive elimination is the rate-determining step in the catalytic cycle. The generation of Pd(ii)-π-allyl complex 9*via* the π-Lewis base activation pathway is a reversible process. Previously observed deuterium incorporation at both the two methyl groups and terminal position of alkenes in deuterium-labeled experiments^8^ validated our computationally predicted pathway.

**Fig. 2:**
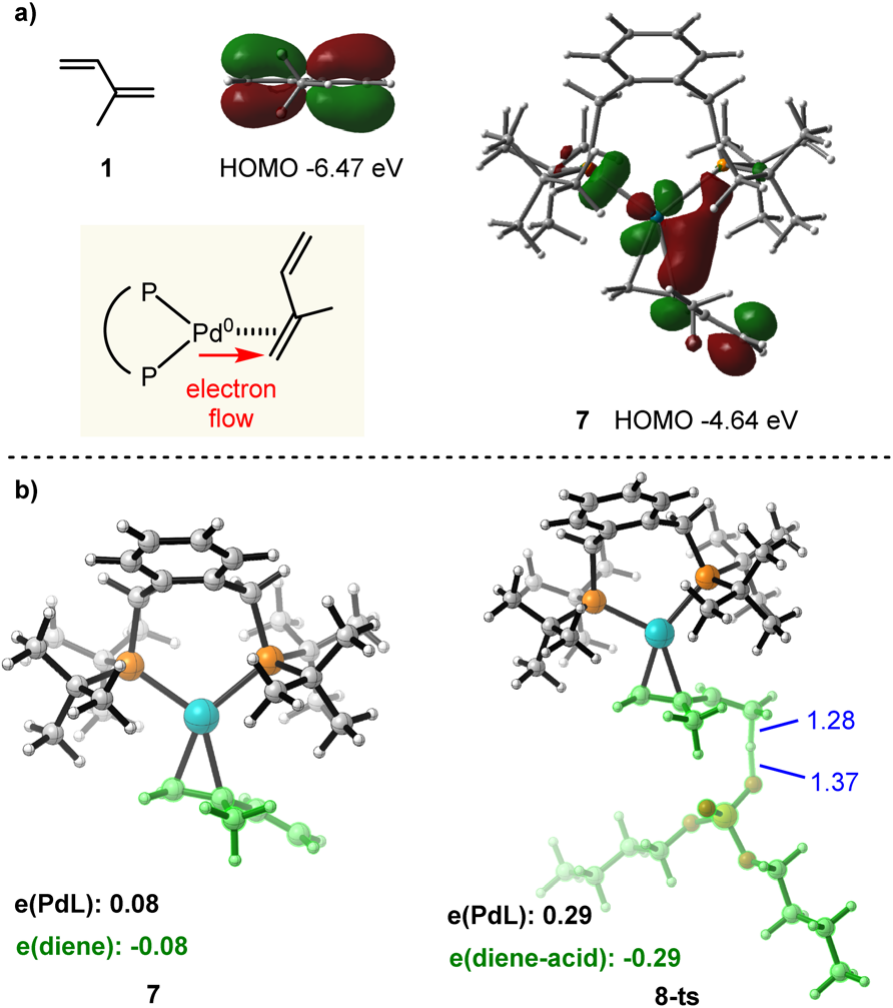
(a) Calculated frontier molecular orbitals of 1 and 7. (b) Optimized structures and Mulliken charge distribution of 7 and 8-ts.

Next, we turned to investigate the nature of Pd(0) mediated π-Lewis base activation of isoprene. As depicted in our aforementioned π-Lewis base activation mode (Scheme 1b), when the 1,3-diene coordinates to Pd(0), the electron flows to the 1,3-diene from the electron-rich Pd(0) center through back donation, resulting in the increase of HOMO energy for the η^2^-coordinated Pd(0)-diene complex as well as the enhancement of electron density for the 1,3-diene moiety. To validate this theory, we conducted frontier molecular orbital (FMO) and natural population analysis (NPA). As shown in Fig. 2a, the HOMO energy of isoprene 1 is −6.47 eV. The HOMO energy of the η^2^-coordinated Pd(0)-isoprene complex 7 is −4.64 eV, indicating a significant increase in the HOMO energy. The HOMO of 7 is a d-orbital of Pd, which is substantially polarized toward the diene moiety corroborating the back donation. In the diene moiety of 7, the HOMO is mainly located at the distal carbon atom indicating the vinylogous activation of isoprene. The computed Mulliken charge of the PdL fragment in 7 is +0.08, indicating charge transfer from PdL to the diene (Fig. 2b). Meanwhile, the more considerable amount (0.29*e*) of charge transfer in transition state 8-ts further verifies the π-Lewis base activation mode.

**Fig. 3:**
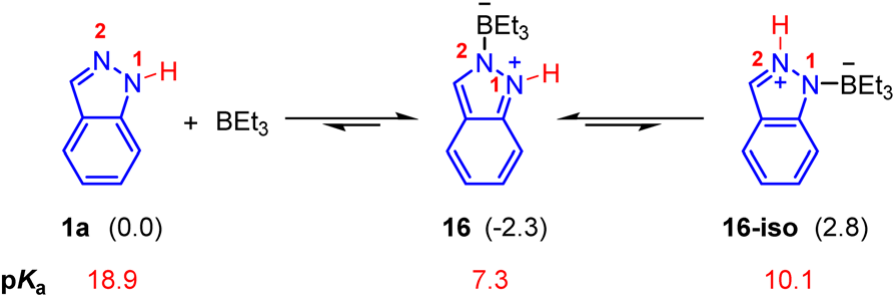
Interaction between indazoles with BEt_3_. The values in parentheses represent relative energy which is given in kcal mol^−1^.

For the Lewis acid BEt_3_ mediated Pd(0)-catalyzed hydroamination of indazoles and isoprene, N^1^-functionalized product 3b was generated (Scheme 2a). Firstly, we investigated the mechanism proposed in a previous report, in which a small amount of BEt_3_ was expected to undergo oxidative addition with Pd(0) and the subsequent hydride elimination to deliver the Et_2_B–Pd(ii)–H species.^8,21^ However, calculated results showed that the activation energy of this pathway is extremely high, which is hard to occur under these reaction conditions (see the ESI for details, Fig. S3†). Thus, we turned to consider the essential role of BEt_3_. As shown in Fig. 3, the combination of indazole 2 and BEt_3_ could generate the Lewis acid–base adduct 16 which is 2.3 kcal mol^−1^ exothermic. The tautomer 16-iso was also considered, but it is 5.1 kcal mol^−1^ endothermic compared with 16. Meanwhile, the predicted p*K*_a_ of the N–H bond in 16 is 7.3, which is much lower than that of 1a (p*K*_a_ = 18.9). It indicates that the acidity of the N–H bond in 16 is significantly enhanced in the presence of BEt_3_. Therefore, we anticipated that 16 could act as an electrophilic partner to drive the catalytic cycle of this Pd(0)-catalyzed hydroamination *via* π-Lewis base activation.

**Fig. 4:**
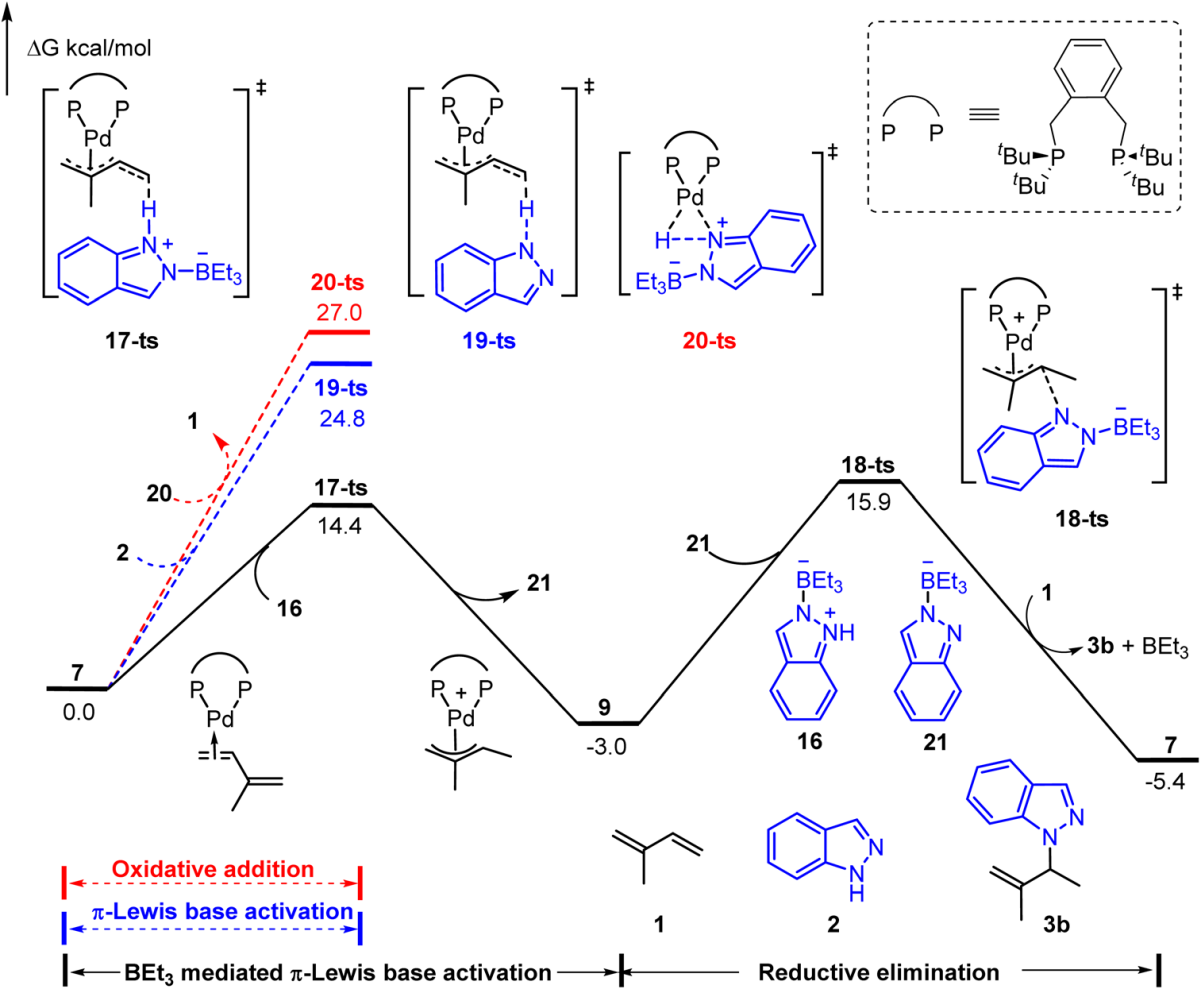
Reaction energy profile of the Lewis acid-mediated Pd-catalyzed hydroamination of indazoles with isoprene.

As shown in Fig. 4, three possible pathways were considered: (1) BEt_3_ mediated π-Lewis base activation (path A: black line); (2) π-Lewis base activation with non-activated indazole (path B: blue line); (3) the oxidative addition of Pd(0) with the N–H bond (path C: red line). The activation energy of path A (*via*17-ts) is 10.4 kcal mol^−1^ lower than that of path B (*via*19-ts). This result correlated with the acidity difference of the N–H bond in 1a and 16, indicating the significant acceleration of BEt_3_ in the π-Lewis base activation pathway. Moreover, the energy barrier of 20-ts (path C) is the highest and up to 27.0 kcal mol^−1^, which suggests that the indazole is insufficient to oxidize Pd(0) directly even under the assistance of BEt_3_. The subsequent outer sphere reductive elimination results in the C–N bond formation *via*18-ts with an energy barrier of 18.9 kcal mol^−1^ in the presence of BEt_3_ stabilized indazole anion 21. These results suggested that the π-Lewis base activation pathway is more favourable in this Lewis acid-mediated case, which lets the Pd-catalyzed hydroamination of 1,3-dienes become a reality in the absence of a Brønsted acid.

**Fig. 5:**
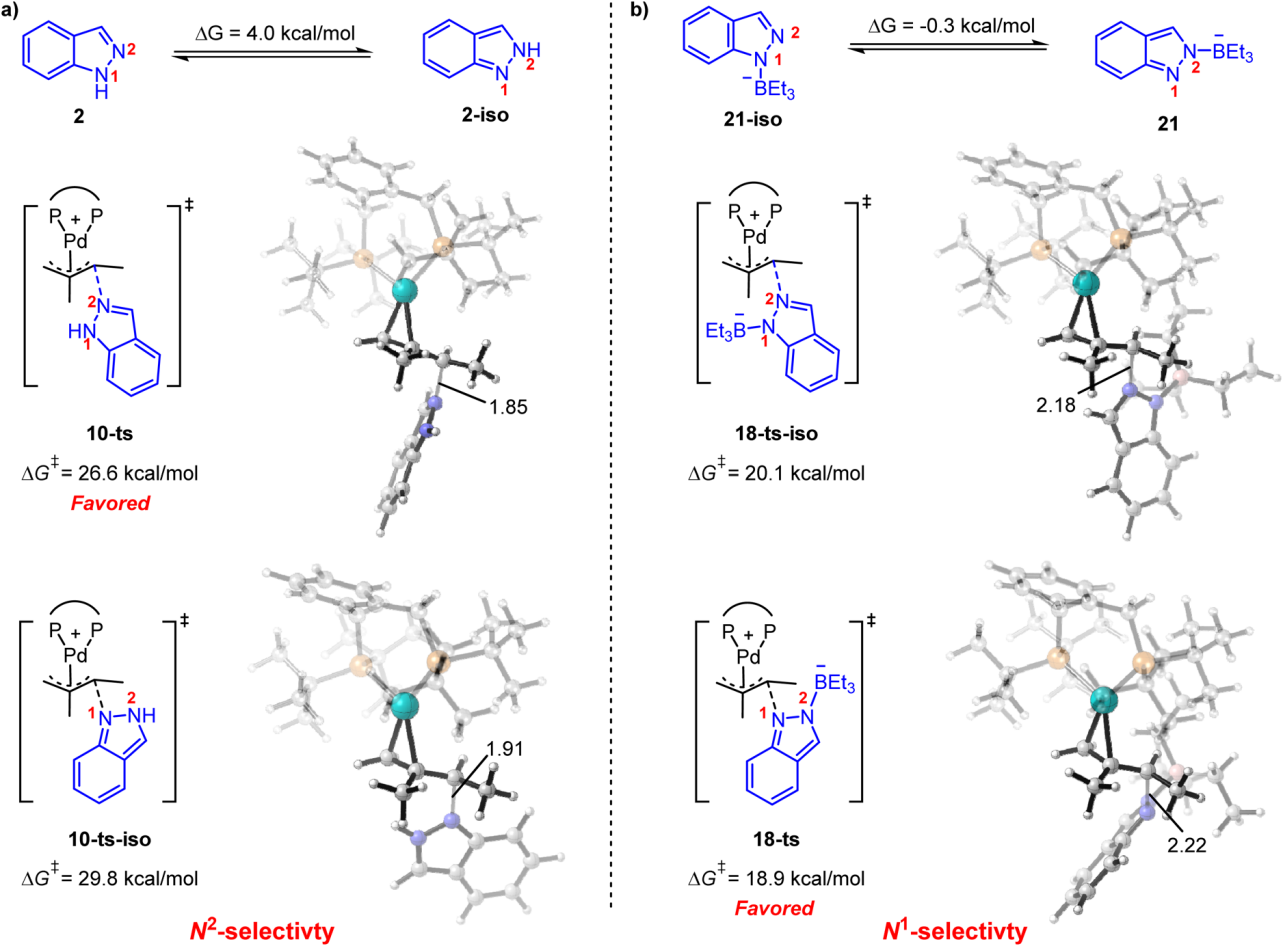
Origin of the N^1^- and N^2^-selectivity for (a) phosphoric acid-mediated hydroamination and (b) Lewis acid-mediated hydroamination. Δ*G*^‡^ in this figure is in kcal mol^−1^ with respect to the corresponding Pd(ii)-π-allyl complex.

It is also noteworthy that the functionalization of the N^1^- or N^2^-position in indazole could be governed by acid co-catalysts.^8^ As shown in Fig. 5a, 1*H*-indazole 2 or 2*H*-indazole 2-iso could be employed as a nucleophile in the reductive elimination step for the phosphoric acid-mediated system. The energy barrier of N^2^-functionalization (*via*10-ts) is 3.2 kcal mol^−1^ lower than that of N^1^-functionalization (*via*10-ts-iso), which is correlated with the energy difference between 2 and 2-iso. It indicates that the N^2^-selectivity is determined by the stability of nucleophiles. In contrast, BEt_3_ stabilized indazole anion 21 and 21-iso were employed as nucleophiles in the BEt_3_-mediated system. 21 and 21-iso exhibit almost the same stability (Fig. 5b). The N^1^-selectivity is determined by the less steric hindrance between BEt_3_ and the ligand in 18-ts. These results suggest that the N^1^- and N^2^-selectivity would be ascribed to the different nucleophiles involved in reductive elimination steps. The predicted selectivity is consistent with experimental observation.

**Fig. 6:**
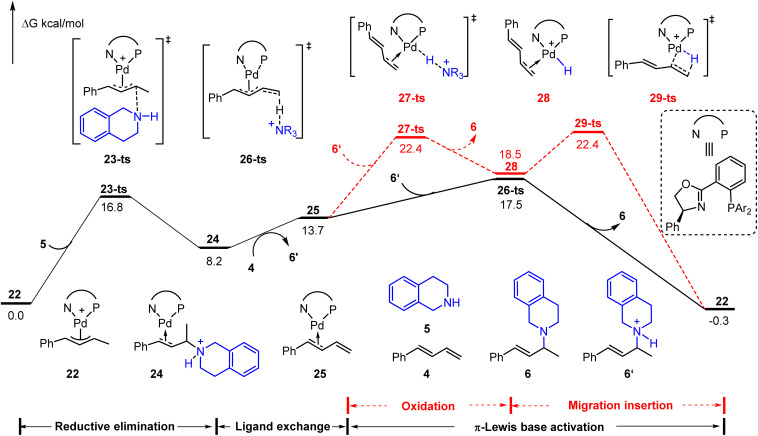
Reaction energy profile of the cationic Pd(ii)-π-allyl mediated Pd-catalyzed hydroamination of aliphatic amine with 1,3-diene.

Besides these hydroamination reactions using a Brønsted acid or Lewis acid as an additive, the cationic Pd(ii)-π-allyl catalyst could achieve the hydroamination of aliphatic amines with 1,3-dienes without an additional acid.^9*b*^ The ammonium salt, which is generated through outer sphere amine attack of the cationic Pd(ii)-π-allyl catalyst, was considered as an initial electrophile to drive the catalytic cycle. As depicted in Fig. 6, starting from cationic Pd(ii)-π-allyl complex 22, the reductive elimination results in the C–N bond formation *via*23-ts with an energy barrier of 16.8 kcal mol^−1^. The subsequent ligand exchange affords the diene-coordinated Pd(0) complex 25 in the presence of phenylbutadiene 4. The activation energy barrier of the Pd(ii)–H species involved pathway (*via*27-ts) is 4.9 kcal mol^−1^ higher than that of the π-Lewis base activation pathway (*via*26-ts). Thus, it further verified the generality of the π-Lewis base activation mode in Pd-catalyzed hydroamination reactions.

**Fig. 7:**
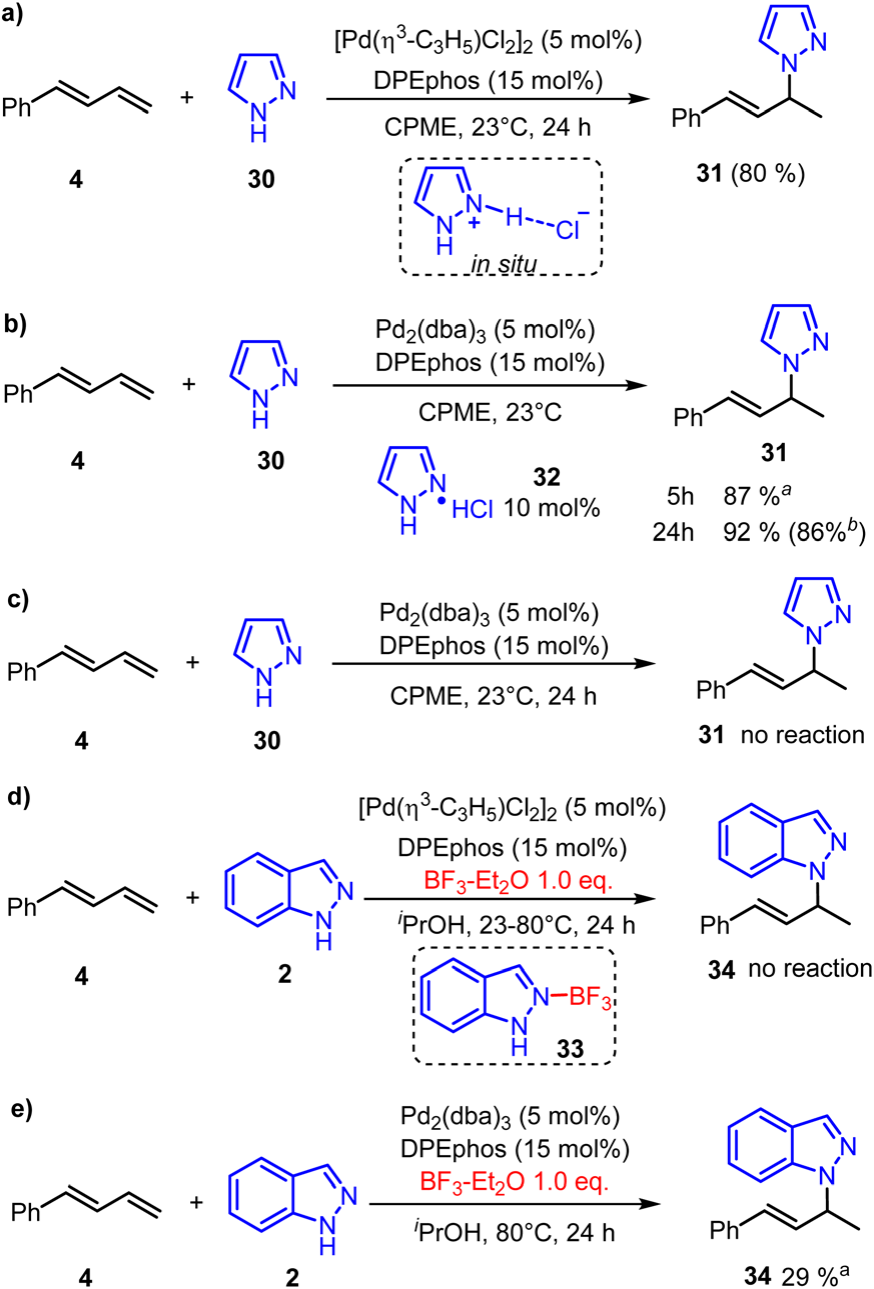
Control experiments. (a) Neutral Pd(ii)-catalyzed hydroamination. (b) Pd(0)-catalyzed hydroamination with hydrochloride. (c) Pd(0)-catalyzed hydroamination without hydrochloride. (d) BF_3_ inhibited Pd(ii)-catalyzed hydroamination. (e) BF_3_ promoted Pd(0)-catalyzed hydroamination. ^*a*^The yield was determined by ^1^H-NMR with mesitylene as the internal standard. ^*b*^Isolated yield.

In addition, the neutral [Pd(π-allyl)Cl]_2_ could also promote the hydroamination reaction of pyrazole and phenylbutadiene in 80% yield after 24 hours (Fig. 7a).^22^ Calculated results showed that the generation of pyrazole hydrochloride from [Pd(π-allyl)Cl]_2_ is more favorable than that of allyl chloride (Fig. S7†). Based on these results, we inferred that the pyrazole hydrochloride generated through reductive elimination of the catalyst with pyrazole could serve as an initial electrophile in the π-Lewis base activation mode. Subsequently, we conducted the reaction by using Pd(0) (Pd_2_(dba)_3_, 5 mol%) and pyrazole hydrochloride (10 mol%) as catalysts (Fig. 7b). To our delight, this reaction was nearly completed in 5 hours giving hydroaminated product 31 in an increased yield of 87%. Extending the reaction time to 24 hours could obtain a slightly higher yield of 92% (86% isolated yield). In addition, the reaction did not work in the absence of pyrazole hydrochloride (Fig. 7c), which emphasized the critical role of the catalytic amounts of pyrazole hydrochloride. Moreover, when the BF_3_ additive (1.0 equiv.) was added, the neutral [Pd(π-allyl)Cl]_2_ catalyzed hydroamination of indazole 2 was completely quenched at 23 °C or 80 °C. We speculate that the combination of 2 and BF_3_ generates Lewis acid-base adduct 33. So the reductive amination of indazole 2 and [Pd(π-allyl)Cl]_2_ was inhibited, and the active Pd(0)-intermediate cannot be generated to realize the catalytic process. Meanwhile, the control trial (Fig. 7e) showed that the corresponding transformation can proceed smoothly when the Pd(0)-catalyst (Pd_2_(dba)_3_, 5 mol%) was used. It suggests that the BF_3_ additive only intervenes in the reductive amination process. On the other hand, it can facilitate the Pd(0)-catalyzed hydroamination as a Lewis acid co-catalyst, which is also realized in Chen's report.^8^ These indicate that the enhanced electrophilicity of pyrazole hydrochloride might be the key reason to react with the Pd(0) activated 1,3-diene and promote the catalytic process.

**Fig. 8:**
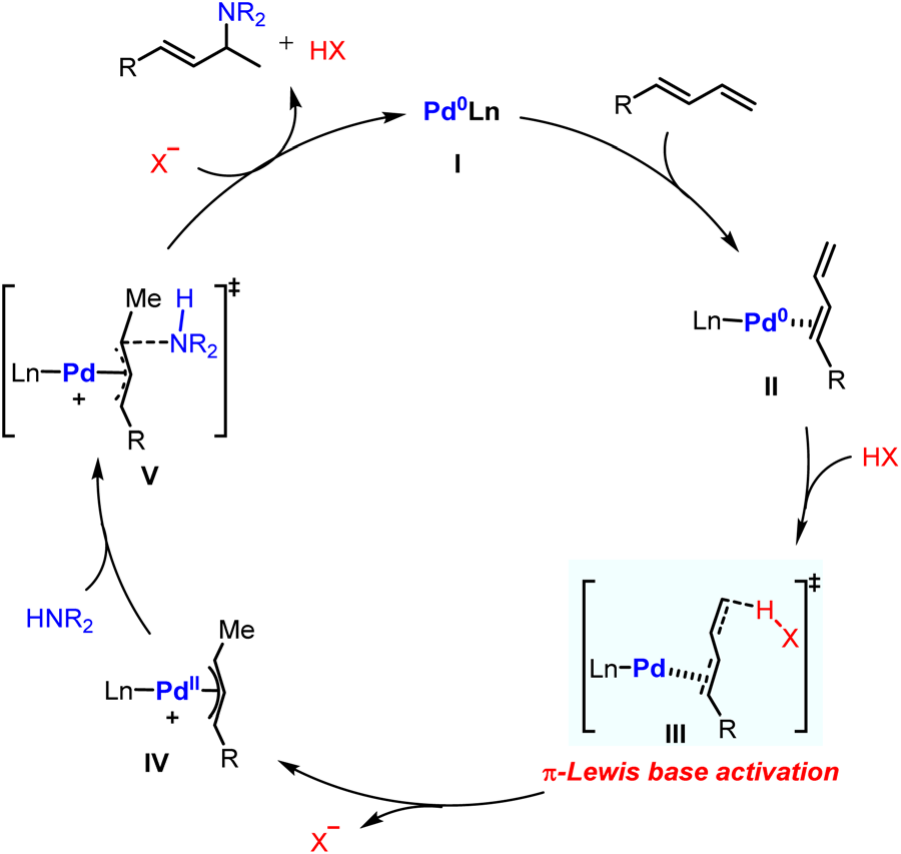
Proposed catalytic cycle for Pd-catalyzed hydroamination of dienes.

Based on the aforementioned results, the proposed catalytic cycle for Pd-catalyzed hydroamination of 1,3-dienes was therefore constructed. The whole catalytic cycle could be divided into two parts: (1) π-Lewis base activation mediated Pd(ii)-π-allyl complex generation; (2) outer sphere reductive elimination guided C–N bond formation. As shown in Fig. 8, the coordination of 1,3-dienes to Pd(0) affords the η^2^-coordinated complex II, in which the electron density for the diene moiety is enhanced and the HOMO energy of II is raised through back donation. Thus, the Pd-π-allyl complex IV could be generated directly after protonation. Ultimately, the reductive elimination releases the hydroaminated product and regenerates the Pd(0) catalyst.

## Conclusions

In summary, we performed a mechanism study on Pd-catalyzed hydroamination of 1,3-dienes. Our mechanistic study showed that the Pd(0)-complex activates 1,3-dienes through back donation *via* a π-Lewis base activation mode. Thus the Pd(0) coordinated 1,3-dienes could react with diverse acidic sources to generate the Pd-π-allyl complexes, in which the unstable Pd(ii)–H species were avoided. FMO and NPA analyses illustrated the increase of HOMO energy for the η^2^-coordinated Pd(0)-diene complexes and the enhancement of electron density for the diene moiety, which revealed the nature of Pd(0) mediated π-Lewis base activation of 1,3-dienes. Calculated results indicated that the π-Lewis base activation pathway is more favourable than the Pd(ii)–H species involved one in the related Pd-catalyzed hydroamination of 1,3-dienes. Different electrophiles including phosphoric acid, Lewis acid-activated indazoles, and Pd(ii) pre-catalyst triggered ammonium salts are sufficient to serve as electrophilic partners. Moreover, further control experiments corroborated our mechanistic proposal, and a more efficient Pd(0) mediated hydroamination was developed. We believe that this study will aid in the understanding of Pd-catalyzed hydroamination, and it will provide a practical theoretical guide for further experimental investigations.

## Data availability

All experimental procedures, characterization, and computational data for this study, can be found in the ESI.†

## Author contributions

The manuscript was written through the contributions of all authors. All authors have approved the final version of the manuscript.

## Conflicts of interest

The authors declare no competing financial interest.

## Supplementary Material

SC-014-D2SC05835A-s001
