# Supplementary material for: A metal π-Lewis base activation model for Pd-catalyzed hydroamination of amines and 1,3-dienes
Source: Chem Sci. 2023 Apr 3;14(17):4597–604. doi: 10.1039/d2sc05835a (PMC10155909; doi:10.1039/d2sc05835a)
Supplement: SC-014-D2SC05835A-s001 [file SC-014-D2SC05835A-s001.pdf]

Electronic Supplementary Material (ESI) for Chemical Science.
This journal is © The Royal Society of Chemistry 2023

Electronic Supplementary Information

for

Metal π-Lewis Base Activation Model in Pd-Catalyzed Hydroamination of Amine and 1,3-Dienes

Xiao Yan,†, § Xiu-Ming Yang,†, § Peng Yan,†, § Bo Zhao,† Rong Zeng,† Bin Pan,† Ying-chun Chen,†,‡ Lei Zhu,*† and Qin Ouyang*†

†College of Pharmacy, Third Military Medical University, Shapingba, Chongqing 400038, China

‡Key Laboratory of Drug-Targeting and Drug Delivery System of the Ministry of Education and Sichuan Research Center for Drug Precision Industrial Technology, West China School of Pharmacy, Sichuan University, Chengdu 610041, China

§These authors contributed equally.

Table of Contents

1. Alternative transition states structures in the π-Lewis base activation step S2
2. Alternative transition states structures in the LLHT step S3
3. BEt_3 mediated generation Pd(II)-H species S3
4. The initial cycle of cationic Pd(II)–π-allyl mediated Pd-catalyzed hydroamination S4
5. Alternative transition states structures for the oxidative protonation process S5
6. Neutral pathway for the Pd-catalyzed hydroamination of aliphatic amine with diene S6
7. The pre-activation of $[Pd(\pi\text{-allyl})Cl]_2$ in the neutral catalytic system S7
8. Control experiments S8
9. Detection the complex II and IV by ^{1}H NMR experiments S10
10. Reaction scope of alkyl amine S11
11. Complete References for Gaussian 09 ....S11
12. Calculated energy and geometry for all intermediates and transition states S12

1. Alternative transition states structures in the π-Lewis base activation step

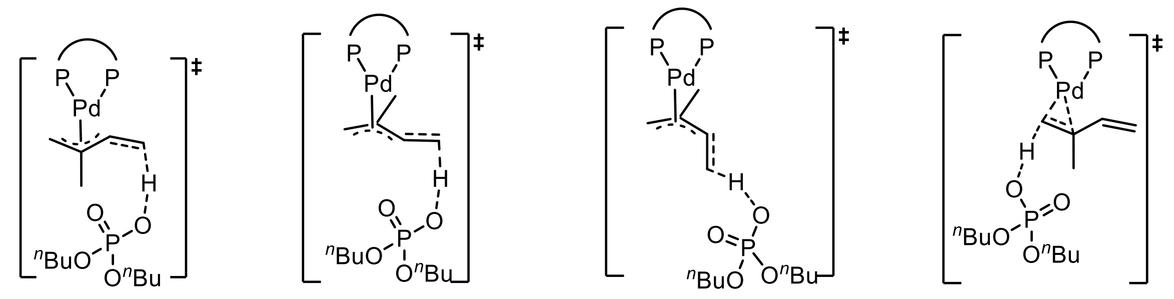

8-ts ΔG = 2.0 kcal/mol

33-ts ΔG = 2.8 kcal/mol

34-ts ΔG = 10.8 kcal/mol

35-ts ΔG = 11.8 kcal/mol

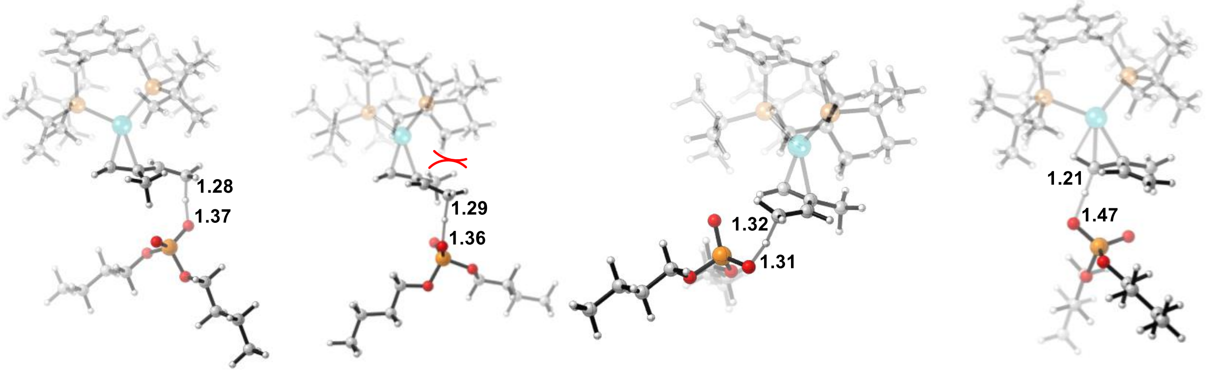

Fig. S1. The structure and energy difference of various π-lewis base activation transition states in Pd-catalyzed hydroamination of indazoles with isoprene.

As shown in **Fig. S1**, alternative transition state structures in the π-Lewis base activation step were considered. Due to the steric hindrance between methyl at isoprene and ligand, the relative free energy of **33-ts** is 0.8 kcal/mol higher than that of **8-ts**. Meanwhile, the cis-conformation isoprene involved transition state **34-ts** is 8.8 kcal/mol higher than that of **8-ts**. In **35-ts**, phosphoric acid reacts with the adjacent carbon atom. and the relative free energy of **35-ts** is 9.8 kcal/mol higher than that of **8-ts**, indicating the vinylogy activation of diene is more favorable. It is consistent with the orbital analysis in Figure 2.

2. Alternative transition states structures in the LLHT step

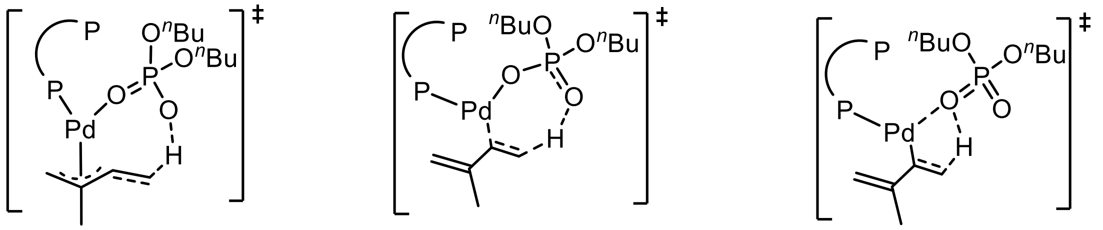

15-ts **36-ts** **37-ts**

Δ*G* = 23.8 kcal/mol Δ*G* = 27.0 kcal/mol Δ*G* = 28.1 kcal/mol

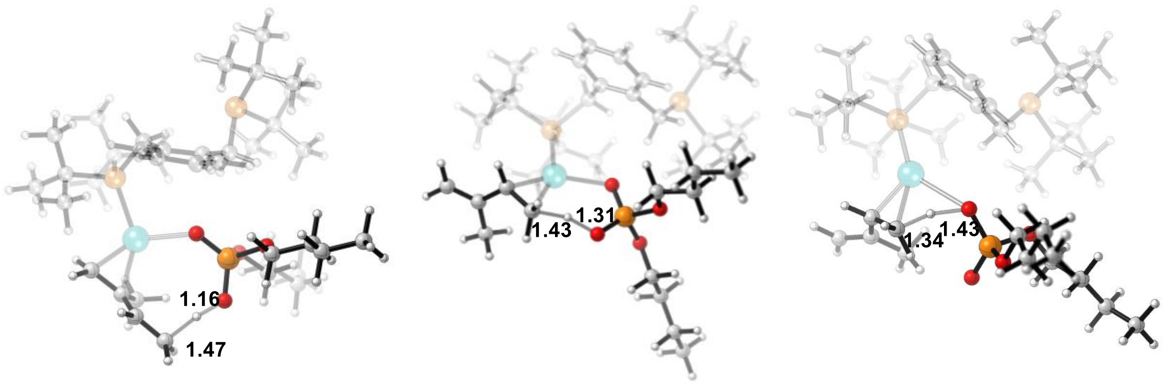

Fig. S2. The structure and energy difference of various LLHT transition state in Pd-catalyzed hydroamination of indazoles with isoprene.

As shown in **Fig. S2**, alternative transition state structures in the LLHT step were considered. Calculated results showed that all of these are much higher than that of **8-ts** for the π-Lewis base activation process.

3. BEt_3 mediated generation Pd(II)-H species

The ancillary Lewis acid BEt_3 could facilitate the Pd(0)-catalyzed hydroamination of indazoles with isoprene to get N^1-functionalized product **3b** (**Scheme 2a**). For this Brønsted acid absent system, a **BEt_3** activating Pd(0) mechanism was proposed in the previous report. A small amount of **BEt_3** was expected to undergo oxidative addition with Pd(0) and the subsequent hydride elimination to deliver the Et_2B-Pd(II)-H species. To evaluate the possibility of this pathway, we have calculated the free energy profile. As shown in Figure S3, the activation free energy of this pathway is up to 66.9 kcal/mol (referring to **7→39-ts**). The extremely high activation free energy suggests this process is hard to occur in this reaction condition. Thus the possibility of the BEt_3 mediated generation Pd(II)-H species pathway was excluded.

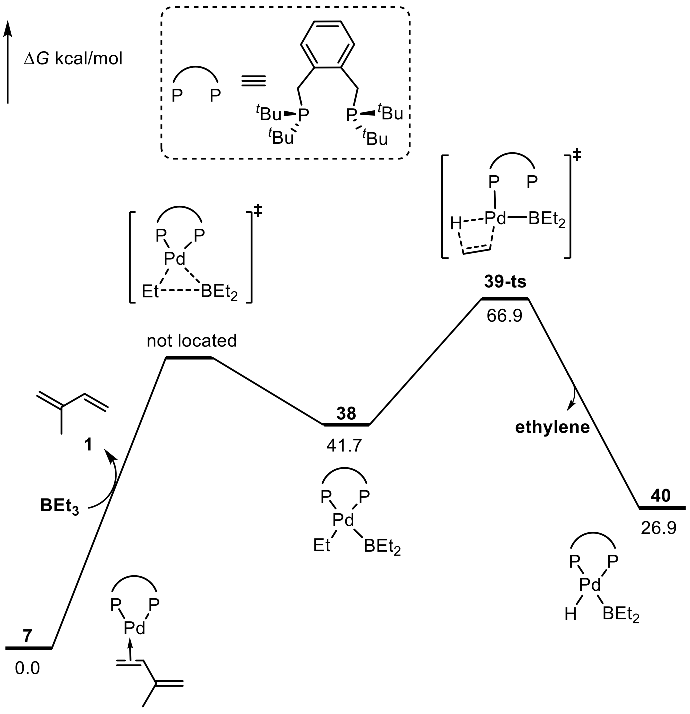

Fig. S3. Free energy profile of the pathway for BEt_3 mediated generation Pd(II)-H species.

4. The initial cycle of cationic Pd(II)–π-allyl mediated Pd-catalyzed hydroamination

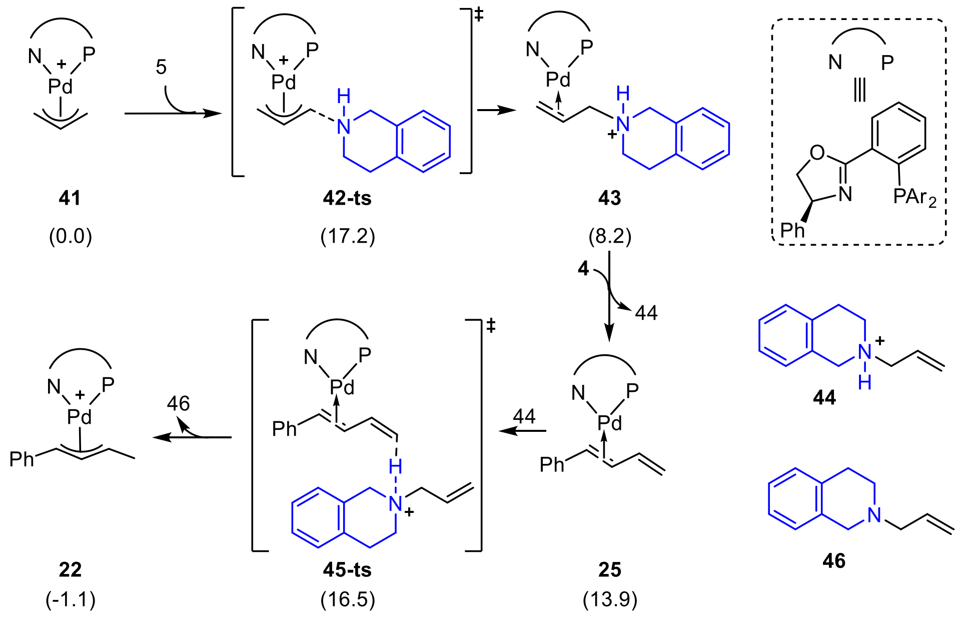

Fig. S4. The initial cycle of cationic Pd(II)–π-allyl mediated Pd-catalyzed hydroamination of aliphatic amine with diene. The data in parentheses represent the relative free energy, which is given in kcal/mol.

The main reaction mechanism of cationic Pd(II)–π-allyl mediated Pd-catalyzed hydroamination of aliphatic amine with diene was given in the text. The active complex **22** could be generated through

the initial cycle of cationic Pd(II)−π-allyl mediated Pd-catalyzed hydroamination (Figure S4). The reaction starts from the amine **5** nucleophilic attack of the Pd(II)-π-allyl species **41** to give Pd(0) complex **25** and quaternary ammonium salt cation **44**. Subsequently, the formation of Pd(II)-π-allyl species **22** via the π-Lewis base activation process between **44** and **25**. The activation free energy for the initial cycle of cationic Pd(II)−π-allyl mediated Pd-catalyzed hydroamination is 17.2 kcal/mol (referring to **41→42-ts**), indicating this process could proceed smoothly in the reaction condition.

5. Alternative transition states structures for the oxidative protonation process

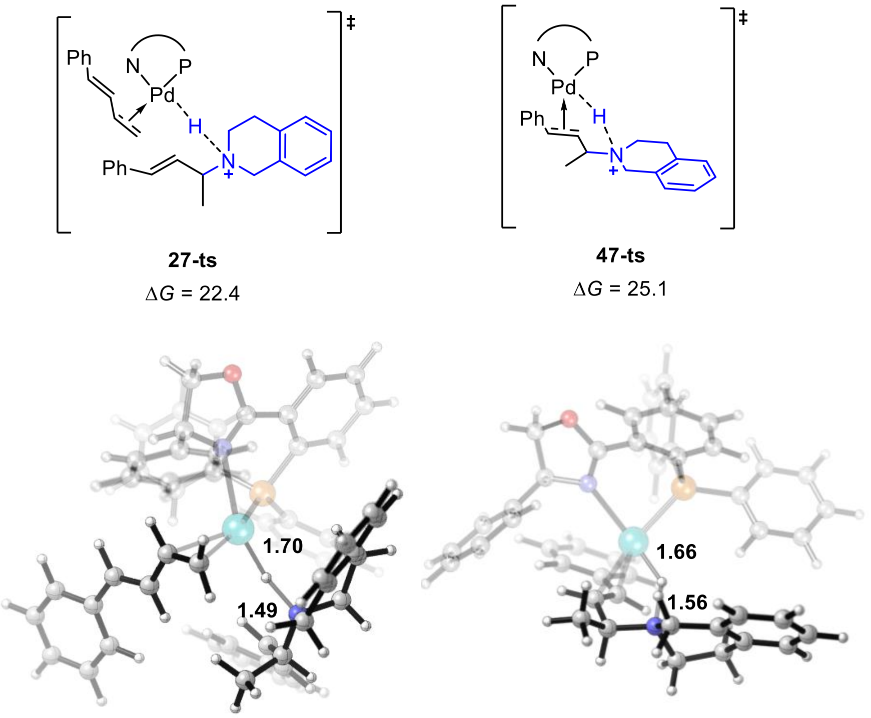

Fig. S5. The structure and energy of different transition states for the generation of Pd(II)-H species via the oxidative protonation process.

As shown in **Fig. S5**, alternative transition state structures in the generation of Pd(II)-H species via the oxidative protonation process were considered. Calculated results showed that all of these are much higher than that of **26-ts** for the π-Lewis base activation process.

6. Neutral pathway for the Pd-catalyzed hydroamination of aliphatic amine with diene

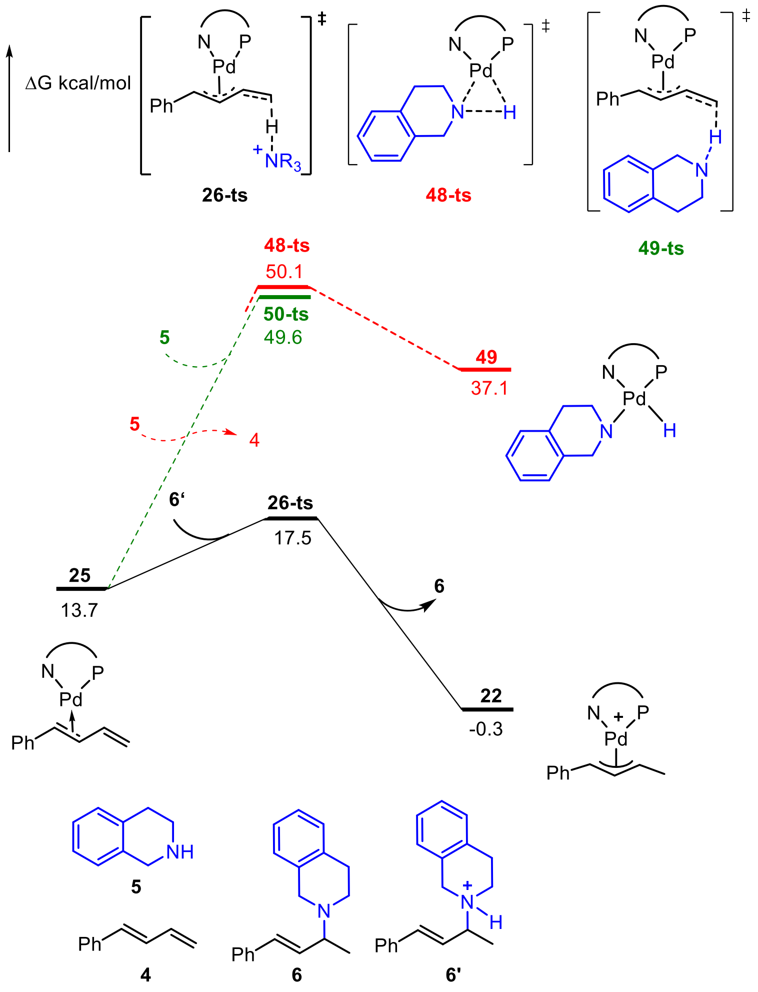

Fig. S6. Free energy profile of the neutral pathway for the Pd-catalyzed hydroamination of aliphatic amine with diene.

Neutral aliphatic amine **5** direct oxidation addition with Pd complexes in hydroamination reaction was discussed. As shown in Figure S6, calculated results show that the energy barrier of oxidation addition with neutral amine **5** is up to 50.1 kcal/mol (referring to **25→48-ts**). It suggests that the neutral amine **5** is insufficient to oxidize Pd(0) species. Moreover, the aliphatic amine **5** involved π-Lewis base activation process needs to overcome a high energy barrier of 49.6 kcal/mol (referring to **25→50-ts**). These results emphasize the critical role of the cationic Pd(II) pre-catalyst triggered ammonium salt **6′**.

7. The pre-activation of [Pd(π-allyl)Cl]₂ in the neutral catalytic system

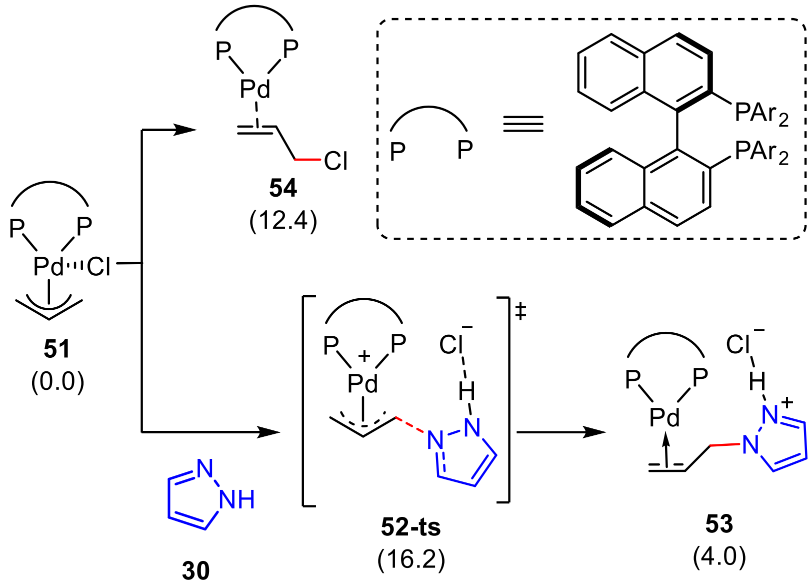

Fig. S7. The pre-activation of $[Pd(\pi\text{-allyl})Cl]_2$ in the neutral catalytic system. The data in parentheses represent the relative free energy, which is given in kcal/mol.

In addition, the neutral $[Pd(\pi\text{-allyl})Cl]_2$ catalyst was also sufficient to mediate hydroamination reactions. Based on the above results, we anticipated that the pyrazole hydrochloride generated through reductive elimination with pyrazole could serve as an initial electrophile of the π-Lewis base activation mode. To validate this issue, we have calculated the pre-activation of $[Pd(\pi\text{-allyl})Cl]_2$ in this neutral catalytic system. As shown in Figure S7, $[Pd(\pi\text{-allyl})Cl]_2$ **51** could directly afford allyl chloride (**54**) with an endothermic by 12.4 kcal/mol. However, the formation of pyrazole hydrochloride **53** via **52-ts** is only endothermic by 4.0 kcal/mol. It suggests that the formation of pyrazole hydrochloride **53** is more thermodynamically favorable. And the generated pyrazole hydrochloride could drive the catalytic cycle via the π-Lewis base activation process to obtain desired hydroaminated products.

8. Control experiments

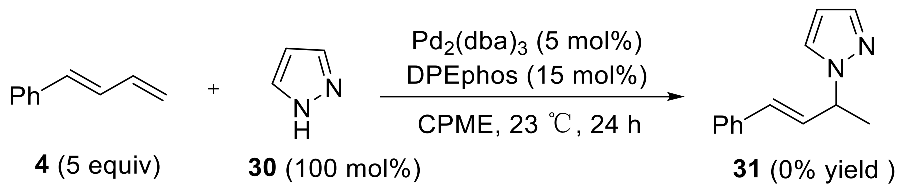

When the catalyst was changed from $[Pd(\eta^3\text{-}C_3H_5)Cl]_2$ to $Pd_2(dba)_3$, the reaction could not proceed normally and no target product was detected. The detailed operations are as follows:

Procedure: In a N_2-filled glovebox, an oven dried vial equipped with a magnetic stir bar was charged with $Pd_2(dba)_3$ (9.2 mg, 0.01 mmol), DPEphos (16.2 mg, 0.03 mmol) and CPME (1.0 mL). The resulting mixture was stirred for 10 mins and then (*E*)-buta-1,3-dien-1-ylbenzene **4** (130.2 mg, 140.2 μL, 1.0 mmol) followed by a solution of freshly recrystallized pyrazole **30** (13.6 mg, 0.2 mmol) in CPME (1.0 mL) were added dropwise. The mixture was removed from the glovebox and stirred at 23 °C for 24 h. TLC and ^{1}H-NMR analysis showed that no target product **31** was formed.

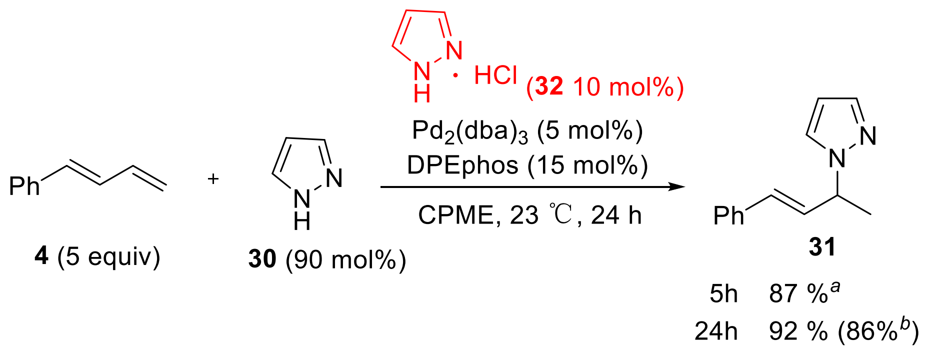

[a]The yield was determined by 1H-NMR with mesitylene as the internal standard.
[b]isolated yield.

However, when a catalytic amount of pyrazole hydrochloride **32** was added, the reaction was back on track and the target product was obtained in a high isolated yield of 87%. Increasing the reaction time to 24 hours could obtain a slightly higher yield of 92% (86% isolated yield).The detailed operations are as follows:

Procedure: In a N_2-filled glovebox, an oven dried vial equipped with a magnetic stir bar was charged with $Pd_2(dba)_3$ (9.2 mg, 0.01 mmol), DPEphos (16.2 mg, 0.03 mmol) and CPME (1.0 mL). The resulting mixture was stirred for 10 mins and then (*E*)-buta-1,3-dien-1-ylbenzene **4** (130.2 mg, 140.2 μL, 1.0 mmol) followed by a solution of freshly recrystallized pyrazole **30** (12.3 mg, 0.18 mmol) and pyrazole hydrochloride **32** (2.1 mg, 0.02 mmol) in CPME (1.0 mL) were added dropwise. The mixture was removed from the glovebox and stirred at 23 °C until no starting material was

observed by TLC. After completion, the mixture was concentrated and purified by flash chromatography on silica gel (EtOAc:petroleum ether = 1:10) to give the product **31** (34.1 mg, 0.172 mmol), as a white solid, yielding 86%. The structure of product **31** was identified by comparison with the NMR spectra of the previous report (*Angew. Chem. Int. Ed.* **2021**, *60*, 19660).

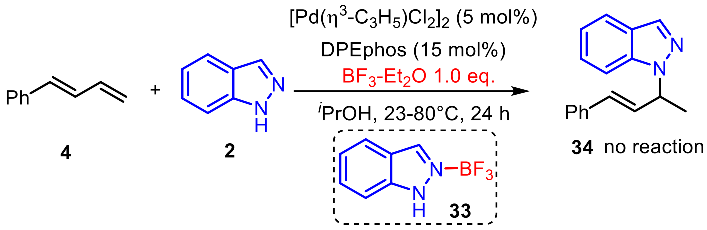

Moreover, when 1.0 equiv BF_3 additive was added, the neutral [Pd(π-allyl)Cl]$_2$ catalyzed hydroamination of indazole **2** was completely quenched at 23°C or 80°C. We speculate that the combination of **2** and BF_3 generates Lewis acid-base adduct **33**. So that the reductive amination of indazole **2** and [Pd(π-allyl)Cl]$_2$ was inhibited, and the active Pd(0)-intermediate cannot generate to realize the catalytic process.

Procedure: In a N_2-filled glovebox, an oven dried vial equipped with a magnetic stir bar was charged with [Pd(π-allyl)Cl]$_2$ (1.83mg, 0.005 mmol), DPEphos (8.1 mg, 0.015 mmol) and ^{i}PrOH (0.50 mL). The resulting mixture was stirred for 10 mins and then (*E*)-buta-1,3-dien-1-ylbenzene **4** (56 μL, 0.4 mmol) followed by a solution of freshly recrystallized indazole **2** (23.6 mg, 0.2 mmol) and BF_3-Et_2O (46.5%, 0.2mmol, 25μL) in ^{i}PrOH (0.50 mL) were added dropwise. The mixture was removed from the glovebox and stirred at 80 ºC until no starting material was observed by TLC. The structure of product **34** was identified by comparison with the NMR spectra of the previous report (*Org. Lett.* **2022**, *24,* 2452).

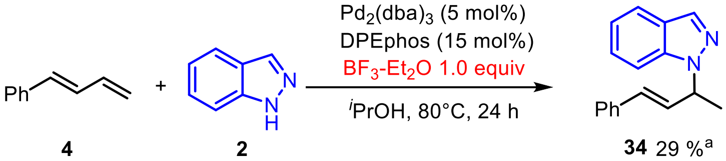

Meanwhile, the controlled trial (Fig 7e) showed that the corresponding transformation can proceed smoothly when Pd(0)-catalyst ($Pd_2(dba)_3$, 5 mol %) was used. It suggests that the BF_3 additive only intervenes in the reductive amination process. On the other hand, it can facilitate the Pd(0)-catalyzed hydroamination as a Lewis acid co-catalyst.

Procedure: In a N_2-filled glovebox, an oven dried vial equipped with a magnetic stir bar was charged with $Pd_2(dba)_3$ (9.2 mg, 0.01 mmol), DPEphos (16.2 mg, 0.03 mmol) and ^{i}PrOH (0.50 mL). The resulting mixture was stirred for 10 mins and then (*E*)-buta-1,3-dien-1-ylbenzene **4** (140 μL, 1

mmol) followed by a solution of freshly recrystallized indazole **2** (23.6 mg, 0.2 mmol) and BF_3-Et_2O (46.5%, 0.2mmol, 25μL) in ^{i}PrOH (0.50 mL) were added dropwise. The mixture was removed from the glovebox and stirred at 80 ºC until no starting material was observed by TLC. The structure of product **34** was identified by comparison with the NMR spectra of the previous report (*Org. Lett.* **2022**, *24,* 2452).

The above control experiments demonstrate that the in situ generated pyrazole hydrochloride is the key to this Pd-catalyzed hydroamination, which is corroborate with our calculation results.

9. Detection the complex II and IV by ^{1}H NMR experiments

we have found the interaction of 1,3-diene with Pd(0)-catalyst via ^{1}H NMR experiments. As shown below, The ^{1}H NMR experiments showed that the diene moiety in **4** experienced apparent high-field shifts when Pd(0)-catalyst and ligand were added, potentially supporting the electron-donating effect of Pd(0) in 1,3-diene coordinated η^2-Pd(0)-complex (**II**) (The similar phenomenon was observed in our previous report: *J. Am. Chem. Soc.*, 2021, **143**, 4809). These results provide reliable evidence for the HOMO-raised η^2-complexes with Pd(0) achieving π-Lewis base activation.

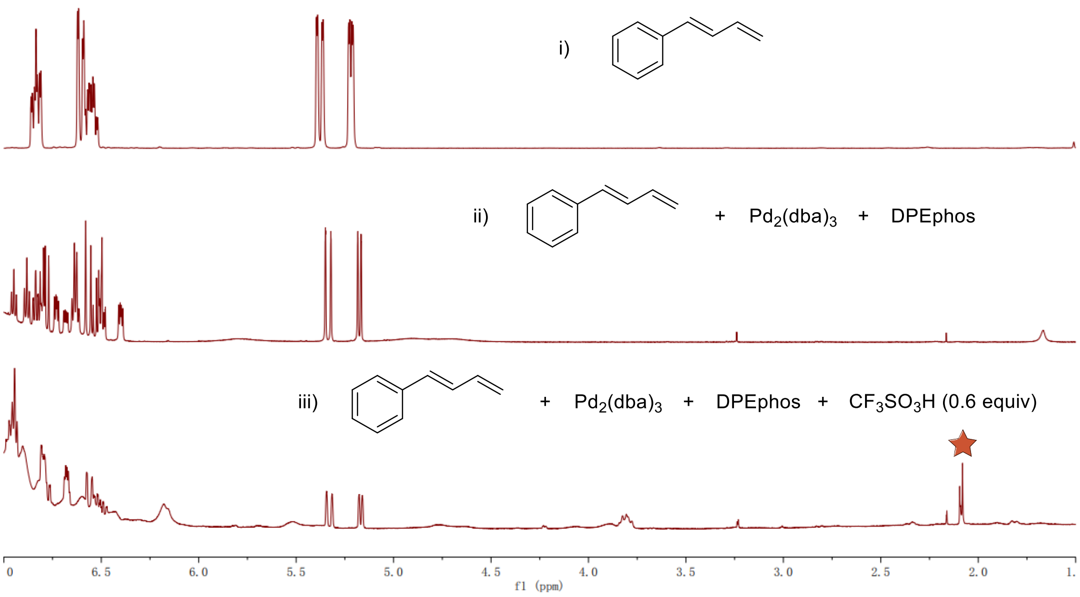

Fig. S8 The ^{1}H NMR experiments of 1,3-diene **4** with adding $Pd_2(dba)_3$, DPEphos, and CF_3SO_3H to trace the **complex IV**.

Complex **III** was considered a transition state, so it cannot be detected by experimental methods. Pd-π-allyl complex **IV** is well known in the Pd-catalyzed reactions. We also try to detect Pd-π-allyl complex **IV** in the reaction system. The 1H NMR experiments showed that when Brønsted acid CF_3SO_3H is added, the characteristic signal for the alkyl group was apparently observed. We

speculate this could be methyl of Pd-π-allyl complex **IV**. This result provides reliable evidence for the Pd-π-allyl complex **IV** could potentially be considered as the reaction intermediate.

10. Reaction scope of alkyl amine

we have evaluated the reactivity of simple alkyl amine with our developed Pd(0)-catalyzed hydroamination of 1,3-diene. The reaction result showed that alkyl amine **35** exhibits lower reactivity compared with pyrazole. It's worth noting that this reaction could proceed with the assistance of pyrazole hydrochloride so that the preparation of other hydrochloride is unnecessary. Moreover, the yield of this reaction can be raised to 74% in the presence of the $AgPF_6$ additive.

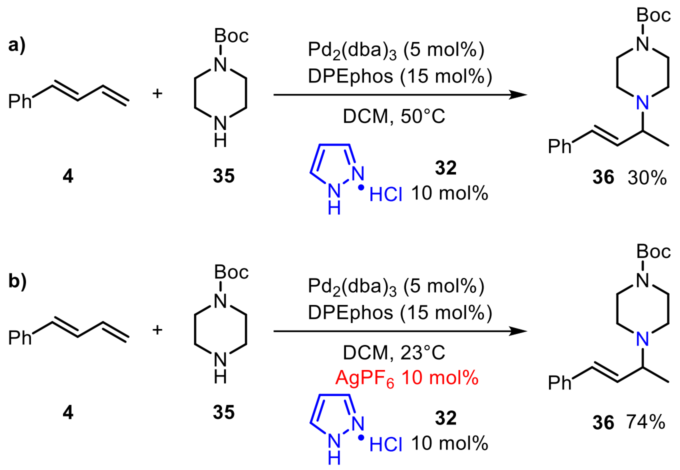

Fig. S9 The reaction scope of alkyl amine.

Procedure: In a N_2-filled glovebox, an oven dried vial equipped with a magnetic stir bar was charged with $Pd_2(dba)_3$ (9.2 mg, 0.01 mmol), DPEphos (16.2 mg, 0.03 mmol) and DCM (0.5 mL). The resulting mixture was stirred for 10 mins and then (*E*)-buta-1,3-dien-1-ylbenzene **4** (130.2 mg, 140.2 µL, 1.0 mmol) followed by a solution of freshly recrystallized piperazine **35** (37.2 mg, 0.2 mmol) and pyrazole hydrochloride **32** (2.1 mg, 0.02 mmol) in DCM (1.0 mL) were added dropwise. The mixture was removed from the glovebox and stirred at 50 ºC until no starting material was observed by TLC. The structure of product **36** was identified by comparison with the NMR spectra of the previous report (*J. Am. Chem. Soc.* **2017**, *139,* 7180).

11. Complete References for Gaussian 09

Gaussian 09, Revision D.01, Frisch, M. J.; Trucks, G. W.; Schlegel, H. B.; Scuseria, G. E.; Robb, M. A.; Cheeseman, J. R.; Scalmani, G.; Barone, V.; Mennucci, B.; Petersson, G. A.; Nakatsuji, H.; Caricato, M.; Li, X.; Hratchian, H. P.; Izmaylov, A. F.; Bloino, J.; Zheng, G.; Sonnenberg, J. L.;

Hada, M.; Ehara, M.; Toyota, K.; Fukuda, R.; Hasegawa, J.; Ishida, M.; Nakajima, T.; Honda, Y.; Kitao, O.; Nakai, H.; Vreven, T.; Montgomery, Jr., J. A.; Peralta, J. E.; Ogliaro, F.; Bearpark, M.; Heyd, J. J.; Brothers, E.; Kudin, K. N.; Staroverov, V. N.; Kobayashi, R.; Normand, J.; Raghavachari, K.; Rendell, A.; Burant, J. C.; Iyengar, S. S.; Tomasi, J.; Cossi, M.; Rega, N.; Millam, N. J.; Klene, M.; Knox, J. E.; Cross, J. B.; Bakken, V.; Adamo, C.; Jaramillo, J.; Gomperts, R.; Stratmann, R. E.; Yazyev, O.; Austin, A. J.; Cammi, R.; Pomelli, C.; Ochterski, J. W.; Martin, R. L.; Morokuma, K.; Zakrzewski, V. G.; Voth, G. A.; Salvador, P.; Dannenberg, J. J.; Dapprich, S.; Daniels, A. D.; Farkas, Ö.; Foresman, J. B.; Ortiz, J. V.; Cioslowski, J.; Fox, D. J. Gaussian, Inc., Wallingford CT, **2013**.

12. Calculated energy and geometry for all intermediates and transition states

1

Zero-point correction= 0.114116 (Hartree/Particle)
Thermal correction to Energy= 0.119951
Thermal correction to Enthalpy= 0.120895
Thermal correction to Gibbs Free Energy= 0.085640
E(solv) = -195.38939962

C	0.65954100	1.40247800	0.00007100
C	0.51598000	-0.10056500	-0.00007700
H	0.18162900	1.84598300	-0.88273700
H	0.17795400	1.84634200	0.88060300
H	1.71227800	1.69922300	0.00218900
C	1.58984500	-0.90969300	-0.00012100
H	2.60314500	-0.51765200	0.00011800
H	1.48668400	-1.99204000	0.00009500
C	-0.83343700	-0.67948900	0.00015600
C	-1.98629500	0.00466400	-0.00006200
H	-2.02058600	1.09056200	-0.00014600
H	-2.94379200	-0.50785400	-0.00016700
H	-0.87112000	-1.76892700	0.00024700

2

Zero-point correction= 0.118564 (Hartree/Particle)
Thermal correction to Energy= 0.124689
Thermal correction to Enthalpy= 0.125633
Thermal correction to Gibbs Free Energy= 0.088311
E(solv)= -379.98585428

C	-0.50801500	0.60256100	0.00000000
C	0.00000000	-0.72265000	0.00000000
C	1.37760700	-0.99669700	0.00000000
C	2.23453700	0.09315200	0.00000000
C	1.74836100	1.42296900	0.00000000
C	0.38851900	1.68815000	0.00000000
C	-1.92804400	0.43453100	0.00000000
H	1.75688100	-2.01457300	0.00000000
H	3.30803100	-0.07587900	0.00000000
H	2.45934000	2.24429900	0.00000000
H	0.02030400	2.71053300	0.00000000
H	-2.69865300	1.19440500	0.00000000
N	-2.26949000	-0.84183900	0.00000000
N	-1.10006500	-1.53633600	0.00000000
H	-1.13680800	-2.54366200	0.00000000

3a

Zero-point correction= 0.236593 (Hartree/Particle)
Thermal correction to Energy= 0.249038
Thermal correction to Enthalpy= 0.249983
Thermal correction to Gibbs Free Energy= 0.197293
E(solv)= -575.39747912

H	-3.46392000	0.56300000	2.09960100
C	-2.37236100	0.03882300	-2.01262800
C	-2.79690800	-0.18875100	-0.58223800
H	-2.39019100	1.10627400	-2.27303400
H	-1.34751900	-0.31402100	-2.17855700
H	-3.03097400	-0.49075700	-2.70698300
C	-3.79882200	-1.01237400	-0.26426500
H	-4.34691300	-1.54646400	-1.03594400
H	-4.10809600	-1.19826500	0.75854100
C	-2.03570400	0.61895900	0.46447600

C	-2.40614700	0.34517100	1.92469500
H	-2.20514900	-0.69764300	2.18197700
H	-1.81025700	0.98446400	2.58336800
H	-2.20993200	1.68254600	0.25040600
C	1.56696200	0.79660100	-0.06002600
C	1.27470300	-0.58365600	0.21530600
C	2.31523800	-1.54734600	0.24205400
C	3.59884500	-1.11540000	-0.00253100
C	3.89178800	0.25535500	-0.27633500
C	2.90045500	1.21011300	-0.30827900
C	0.31664500	1.42026500	-0.00218800
H	2.08913300	-2.58859100	0.45049600
H	4.41732100	-1.83041800	0.01133400
H	4.92301500	0.54306100	-0.46253400
H	3.13276800	2.25104900	-0.51723400
H	0.01171800	2.44769200	-0.13940600
N	-0.58193000	0.44652400	0.28365800
N	-0.04509300	-0.78059200	0.42344600

3b

Zero-point correction=0.236598 (Hartree/Particle)

Thermal correction to Energy=0.249124

Thermal correction to Enthalpy=0.250068

Thermal correction to Gibbs Free Energy=0.196789

E(Solv)= -575.40256649

C	2.11670700	-0.56320900	-1.94419700
C	2.61121600	-0.32242900	-0.53841200
H	1.63381400	-1.54575700	-2.03759300
H	1.37010400	0.18657700	-2.23143400
H	2.93910800	-0.51855000	-2.66414800
C	3.88111700	0.00268400	-0.28275600
H	4.60459400	0.10076300	-1.08794100
H	4.25109800	0.19268500	0.71902100
C	1.57272900	-0.51725100	0.56313300
C	2.04749300	-0.22215400	1.99008400
H	2.34581600	0.82543500	2.08566000
H	2.89418000	-0.86103000	2.25941200
H	1.24898600	-1.56574900	0.51573800
C	-1.72952100	0.96371000	-0.08696300
C	-0.93469000	-0.18989300	0.14729900
C	-1.50163000	-1.47557700	0.21558700
C	-2.87368500	-1.57326400	0.04275800
C	-3.67915800	-0.43309400	-0.19308000
C	-3.12040800	0.83258100	-0.25964500
C	-0.79103200	2.03822800	-0.07973200
H	-0.89849600	-2.36009500	0.39710200
H	-3.34450200	-2.55156900	0.08997300
H	-4.75024100	-0.55965600	-0.32256000
H	-3.74025900	1.70670500	-0.44042900
H	-0.97207800	3.09608600	-0.22187100
N	0.43949300	1.60667000	0.13364100
N	0.35894900	0.25426600	0.26608500
H	1.23396000	-0.41437700	2.69653600

4

Zero-point correction= 0.167365 (Hartree/Particle)

Thermal correction to Energy= 0.176323

Thermal correction to Enthalpy= 0.177268

Thermal correction to Gibbs Free Energy= 0.132588

E(solv)= -387.20701714

C	-0.89639100	-0.66149100	-0.00011300
C	-1.98196100	0.14281400	0.00003100
H	-1.06886200	-1.73856300	-0.00028400
H	-1.86416800	1.22590300	0.00022200
C	-3.34610600	-0.35165400	-0.00004200
C	-4.43904200	0.42949000	0.00011300
H	-3.46700500	-1.43551200	-0.00024500
C	0.51375100	-0.26592200	-0.00006000
C	1.50040000	-1.27030700	0.00003000
C	0.94328900	1.07659900	-0.00010500
C	2.85755500	-0.95313300	0.00009600
H	1.19080700	-2.31303800	0.00005800
C	2.29795800	1.39403300	-0.00003700
H	0.21127000	1.87888100	-0.00021500
C	3.26380800	0.38210600	0.00006700
H	3.59745400	-1.74924200	0.00016700
H	2.60440100	2.43689800	-0.00007100
H	4.32067700	0.63430000	0.00011400
H	-5.44056300	0.01050500	0.00005400
H	-4.36358500	1.51466000	0.00031800

5

Zero-point correction= 0.183110 (Hartree/Particle)

Thermal correction to Energy= 0.190826

Thermal correction to Enthalpy= 0.191771

Thermal correction to Gibbs Free Energy= 0.150823

E(solv) = -404.49274818

C	-1.18463300	1.45974300	-0.04489200

C	0.12353900	0.69091400	0.00165400
C	0.13143100	-0.71314000	0.04183200
C	-1.17190100	-1.48764900	0.10214800
C	-2.36057700	-0.62953100	-0.33739800
H	1.32781000	2.47376400	-0.06960800
H	-1.32190000	1.84418900	-1.07721500
C	1.33928200	1.38564500	-0.03874400
C	1.36108500	-1.38627500	0.04265700
H	-1.10476700	-2.38948100	-0.52002300
H	-3.30152100	-1.13704600	-0.09852300
C	2.56668500	-0.68943200	0.00475900
C	2.55592500	0.70737800	-0.03637700
H	1.36672100	-2.47427100	0.07210300
H	3.50901200	-1.23103300	0.00508200
H	3.48940900	1.26329600	-0.06382000
N	-2.30838000	0.63521200	0.39534300
H	-3.17459900	1.15231900	0.26527100
H	-1.11914800	2.33886600	0.60848500
H	-2.33015300	-0.48953000	-1.43641000
H	-1.34721700	-1.82346800	1.13342700

6

Zero-point correction= 0.243101 (Hartree/Particle)
Thermal correction to Energy= 0.256411
Thermal correction to Enthalpy= 0.257355
Thermal correction to Gibbs Free Energy= 0.200191
E(Solv)= -613.52886444

C	3.80322000	-1.51756500	-0.57991900
C	2.51082900	-1.00182400	-0.61655200
C	2.20941500	0.23764100	-0.02064200
C	3.25404600	0.93133200	0.61578500
C	4.54939100	0.41632600	0.65347200
C	4.83073000	-0.81183800	0.05422400
H	4.01090100	-2.47718700	-1.04624800
H	1.72771400	-1.57131400	-1.10849800
H	3.04237400	1.88953300	1.08530700
H	5.33756800	0.97482100	1.15159700
H	5.83803200	-1.21829700	0.08170200
C	0.86491100	0.83844800	-0.02836400
H	0.78972400	1.76999000	0.53003100
C	-0.22993500	0.36691900	-0.64329700
H	-0.17612000	-0.56296100	-1.20768800
C	-1.60564200	1.00077600	-0.63611800
C	-1.69215600	2.34178800	0.11792600
H	-1.45265800	2.21130200	1.17818500
H	-1.00127900	3.07741000	-0.30839100
H	-1.86813400	1.19977700	-1.68938500
C	-3.87142200	-1.48727300	0.94709700
C	-2.59035600	-0.89061900	0.93644500
C	-2.62445600	0.01368700	-0.11355500
N	-3.87620200	-0.09850100	-0.64273500
H	-4.25332400	-2.25042400	1.61309400
H	-1.75359100	-1.08632900	1.59105200
N	-4.65677600	-1.00664800	-0.01609700
H	-2.70663100	2.74800300	0.05357800
H	-4.25517600	0.40493200	-1.43151300

7

Zero-point correction= 0.750691 (Hartree/Particle)
Thermal correction to Energy= 0.792039
Thermal correction to Enthalpy= 0.792983
Thermal correction to Gibbs Free Energy= 0.681307
E(solv)= -1947.68693849

Pd	-0.40337300	-0.95832000	-0.49870200
P	1.87911400	-0.75359000	0.23858700
P	-1.54384200	0.99473500	0.32848600
C	2.32495500	0.84139500	1.17855600
C	2.07505900	2.19682500	0.54366800
C	3.18022600	2.91197200	0.04904300
C	3.07078700	4.19294100	-0.48502300
C	1.82485500	4.81551900	-0.50276800
C	0.72670400	4.14274600	0.02503700
C	0.81101500	2.83718200	0.54151000
C	-0.43157700	2.27012700	1.20042100
C	3.16153700	-0.83237400	-1.20806000
C	2.57763400	0.06145100	-2.32708400
C	4.58924000	-0.34751400	-0.88919900
C	3.25454800	-2.27404400	-1.74959800
C	2.37070800	-2.05603000	1.59539300
C	1.63220700	-1.68703500	2.90209900
C	3.87531800	-2.15776700	1.90924900
C	1.84801900	-3.44297700	1.16488600
C	-2.41252100	2.00385400	-1.07408300
C	-1.32370900	2.25925500	-2.14318800
C	-3.04111600	3.34096600	-0.64220500
C	-3.50139100	1.14502600	-1.75141100
C	-2.77148700	0.68570400	1.80222400
C	-3.14853800	1.92632700	2.63852700

C	-4.06822200	0.02004900	1.30092500
C	-2.05955100	-0.32089300	2.73240100
H	1.72687900	0.76742400	2.09102400
H	3.37339300	0.78920200	1.49293500
H	4.16485700	2.45992000	0.11435000
H	3.95414200	4.70399100	-0.85804200
H	1.71067000	5.82263100	-0.89433100
H	-0.23264300	4.65225200	0.05749500
H	-1.05570500	3.11322900	1.50918000
H	-0.14617400	1.75377000	2.12422500
H	1.58279000	-0.28078900	-2.62884400
H	2.49480900	1.10747900	-2.01813200
H	3.23839400	0.02192000	-3.20441400
H	4.61599600	0.70353000	-0.59936200
H	5.07362400	-0.93099600	-0.10373900
H	5.20561600	-0.44419000	-1.79369600
H	3.79804700	-2.26045500	-2.70418800
H	3.80675400	-2.93576700	-1.07513100
H	2.26955100	-2.70943300	-1.93271300
H	0.56819600	-1.50116500	2.72836400
H	1.71218400	-2.52656300	3.60538900
H	2.06473100	-0.81281600	3.39907400
H	4.31773700	-1.19072700	2.17246200
H	4.01950700	-2.82425600	2.77100900
H	4.44583700	-2.58130500	1.07807700
H	2.30421600	-3.80081700	0.23931100
H	2.07781800	-4.17461700	1.95181900
H	0.76447900	-3.42510700	1.01972600
H	-1.78322700	2.75622200	-3.00870700
H	-0.51317900	2.89589400	-1.78482700
H	-0.88507500	1.31600500	-2.48668400
H	-3.90726800	3.19305500	0.01035600
H	-2.33650300	4.00031400	-0.12648100
H	-3.39617000	3.87981700	-1.53149100
H	-3.79484400	1.62680500	-2.69413600
H	-3.13356200	0.14319000	-1.98301600
H	-4.40250600	1.04476700	-1.14422200
H	-3.87593700	1.62849700	3.40612200
H	-2.29093400	2.35795300	3.16362100
H	-3.61363400	2.71499200	2.04088600
H	-3.87925500	-0.79372300	0.59418100
H	-4.60771400	-0.40084200	2.16073700
H	-4.73968900	0.74227000	0.82774500
H	-1.82129900	-1.24874000	2.20347200
H	-1.12993300	0.08057500	3.15081900
H	-2.71765200	-0.56401800	3.57792300
C	-2.50332900	-3.88366600	0.17197800
C	-2.85827700	-2.95808000	-0.97041400
H	-1.89869800	-4.73152100	-0.17539200
H	-1.91053100	-3.36303000	0.93440300
H	-3.40241300	-4.28764600	0.64761900
C	-4.14565600	-2.79236600	-1.33174900
H	-4.95509600	-3.29328300	-0.80739200
H	-4.42756800	-2.15691300	-2.16789700
C	-1.76781500	-2.28629800	-1.70597300
C	-0.42828800	-2.73135100	-1.73270900
H	-2.10898900	-1.69825600	-2.55734700
H	0.16295300	-2.53558500	-2.62547300
H	-0.12892100	-3.62327200	-1.18714800

8-ts

Zero-point correction= 1.026340 (Hartree/Particle)
Thermal correction to Energy= 1.085841
Thermal correction to Enthalpy= 1.086786
Thermal correction to Gibbs Free Energy= 0.926309
E(solv)= -2906.62578869

Pd	1.31511000	0.07065000	-0.19985800
P	2.87615700	1.88195400	0.02274000
P	2.66773000	-1.90758800	-0.71728700
C	4.70198300	1.35459700	0.00075200
C	5.22130800	0.29458000	0.95758200
C	5.94059700	0.71331300	2.09028200
C	6.54255700	-0.17943800	2.97282600
C	6.47245600	-1.54440300	2.70825800
C	5.80249000	-1.97787600	1.56777200
C	5.15169400	-1.09554300	0.68841200
C	4.54348500	-1.67029700	-0.57638400
C	2.69963100	2.92241200	1.64584300
C	2.44929700	1.89935100	2.77765100
C	3.92886300	3.77665200	2.02000300
C	1.48916500	3.87724200	1.56611800
C	2.85318600	3.07703600	-1.50710100
C	3.36974000	2.29822100	-2.73614000
C	3.70893600	4.34863900	-1.34772500
C	1.39662900	3.47843000	-1.82392800
C	2.25347800	-3.32152100	0.52877600
C	2.16606300	-2.64761900	1.91846500
C	3.26562900	-4.48085700	0.61133000

C	0.86851400	-3.90686400	0.18880500
C	2.56482100	-2.58574300	-2.53312800
C	3.10813500	-4.01425900	-2.72905700
C	1.09458300	-2.53259700	-2.99353300
C	3.36754700	-1.64739600	-3.46127400
H	4.85386400	1.00792800	-1.02433100
H	5.31473100	2.25540200	0.11126800
H	6.06649800	1.77565900	2.26785900
H	7.08493500	0.19344200	3.83706200
H	6.95778300	-2.26433500	3.36114300
H	5.81095900	-3.03714200	1.33229800
H	5.02626800	-2.62927000	-0.79393700
H	4.78493700	-1.01240200	-1.41543700
H	1.54233200	1.31486200	2.59895500
H	3.28327900	1.20033800	2.89206600
H	2.32881200	2.43538300	3.72865500
H	4.80884500	3.17319600	2.23974300
H	4.19549700	4.50403700	1.25079000
H	3.69462500	4.33991200	2.93295800
H	1.29216000	4.27499000	2.57027100
H	1.68281700	4.73439100	0.91454900
H	0.57799800	3.38569800	1.22433900
H	2.84061800	1.34861500	-2.85610600
H	3.18445400	2.89585700	-3.63780500
H	4.44554800	2.10109300	-2.69931800
H	4.74750700	4.12838200	-1.07871900
H	3.73040900	4.88524800	-2.30567300
H	3.29873500	5.03718500	-0.60455100
H	0.90975200	4.01587300	-1.00834600
H	1.39278100	4.13810400	-2.70167300
H	0.79025100	2.60043600	-2.06285600
H	1.87516100	-3.40025700	2.66375200
H	3.12225600	-2.21971300	2.23215300
H	1.41569700	-1.85139800	1.93105700
H	3.36816700	-5.02867200	-0.32670600
H	4.25689800	-4.15051200	0.92479500
H	2.91463000	-5.19539400	1.36783400
H	0.53473000	-4.53951700	1.02182000
H	0.11763800	-3.12798100	0.03995000
H	0.89155800	-4.53711000	-0.70531500
H	3.11346200	-4.24782700	-3.80223200
H	4.13655000	-4.12777900	-2.36927100
H	2.48578600	-4.76831400	-2.24085500
H	0.70750900	-1.50996400	-2.96155200

H	1.02797500	-2.88917500	-4.03017400
H	0.43535700	-3.15485000	-2.38605300
H	3.10506800	-0.59652000	-3.31145800
H	4.44989800	-1.75772400	-3.34252600
H	3.13163800	-1.89732200	-4.50351200
C	-1.36005300	-0.88172300	1.55889400
C	-0.96873700	-0.19280800	0.26980400
H	-2.44445800	-0.76009200	1.69137300
H	-1.13765800	-1.95370900	1.54938500
H	-0.84366800	-0.43384100	2.41476500
C	-0.46032000	1.14082200	0.29546300
H	-0.67679100	1.79897900	-0.54423000
H	-0.45722500	1.64330300	1.25897100
C	-1.31810800	-0.74701400	-0.98360200
C	-2.09891800	-1.91157600	-1.22058100
H	-1.99442800	-2.35620100	-2.21295800
H	-2.14486900	-2.65204400	-0.42062600
H	-1.11933600	-0.11451900	-1.84981000
P	-5.18162300	-0.27030600	-0.24827400
O	-4.45316100	-0.25314800	1.06254100
O	-4.62014800	-1.12456600	-1.39906600
H	-3.29804800	-1.46351000	-1.26905600
O	-6.76233100	-0.66308000	-0.05082700
O	-5.37605100	1.22026200	-0.88263700
C	-7.05860900	-1.85920300	0.67743100
H	-6.57466800	-1.81848000	1.66188600
H	-6.65422600	-2.72655900	0.13600300
C	-8.57128100	-1.97674400	0.81610700
H	-8.94768000	-1.08102900	1.32867200
H	-9.01813200	-1.97473500	-0.18706400
C	-9.00041400	-3.23619700	1.57941600
H	-8.53833000	-3.23426700	2.57697700
H	-8.60985700	-4.12589000	1.06497600
C	-10.52119300	-3.35939700	1.72349100
H	-10.80004100	-4.26710500	2.27155400
H	-10.93784400	-2.50102300	2.26488900
H	-11.00934500	-3.39842600	0.74185100
C	-5.86316100	2.26455200	-0.03133100
H	-6.91204200	2.06331100	0.22545600
H	-5.28295200	2.27911900	0.89999200
C	-5.73737200	3.58852700	-0.77494800
H	-6.28896400	3.51518300	-1.72175800
H	-4.68246300	3.74554200	-1.03923100
C	-6.25554200	4.77814100	0.04319400

H	-7.30830100	4.60703700	0.30926200
H	-5.70733900	4.83437900	0.99458700
C	-6.12968200	6.11435800	-0.69666900
H	-6.69630200	6.10091400	-1.63588600
H	-6.50774800	6.94603400	-0.09050100
H	-5.08350300	6.33146400	-0.94634300

9

Zero-point correction=0.765533 (Hartree/Particle)

Thermal correction to Energy=0.807061

Thermal correction to Enthalpy=0.808005

Thermal correction to Gibbs Free Energy=0.696417

E(solv)= -1948.17224206

Pd	-0.26012100	-1.13831900	-0.38489200
P	1.94493700	-0.37243300	0.23771200
P	-1.73374000	0.69200600	0.35592400
C	2.01790600	1.32503900	1.07382400
C	1.49660700	2.60095200	0.43902100
C	2.42042600	3.52061700	-0.08550600
C	2.04189700	4.77736400	-0.55099000
C	0.70949800	5.16702800	-0.44716500
C	-0.21247200	4.28697400	0.11346100
C	0.14130900	2.99483100	0.53601500
C	-0.90679100	2.14915800	1.22956500
C	3.16483900	-0.23647300	-1.25866800
C	2.36047600	0.41387300	-2.40677600
C	4.40413700	0.63846400	-0.97272900
C	3.68338900	-1.61329000	-1.72255600
C	2.70450700	-1.44501400	1.66840100
C	1.85483100	-1.22108400	2.94088700
C	4.17295800	-1.09517200	1.98812300
C	2.62163900	-2.94815200	1.33809500
C	-2.66050600	1.47813300	-1.14366400
C	-1.59193200	1.69732200	-2.24019100
C	-3.35432000	2.82258600	-0.84653600
C	-3.72135100	0.51102400	-1.69763400
C	-2.96081000	0.14490500	1.74954200
C	-4.10432600	1.14308800	2.01612400
C	-3.56480100	-1.22432900	1.39028500
C	-2.15574500	-0.03859000	3.05677200
H	1.49120500	1.16484600	2.01738100
H	3.06784400	1.47801700	1.33980800
H	3.47556200	3.27096200	-0.08377000
H	2.78976200	5.45580400	-0.95026500
H	0.39352600	6.15665400	-0.76313300
H	-1.23023500	4.63096500	0.26527900
H	-1.71166500	2.80395400	1.57808600
H	-0.47227000	1.70376300	2.12847600
H	1.51109000	-0.20510800	-2.71191000
H	1.98053700	1.40305800	-2.13421800
H	3.01614000	0.53788100	-3.27789700
H	4.14648200	1.68024200	-0.79535100
H	5.00254100	0.27841200	-0.13404000
H	5.04711100	0.61817000	-1.86121800
H	4.27376300	-1.46446600	-2.63507500
H	4.34664400	-2.07695300	-0.98690700
H	2.88824600	-2.31613000	-1.96768300
H	0.78561400	-1.35698400	2.74686300
H	2.14836300	-1.96383600	3.69244300
H	2.00899700	-0.23712700	3.39218300
H	4.32751000	-0.03349600	2.20211800
H	4.47372100	-1.64863200	2.88642200
H	4.85469400	-1.39134700	1.18668400
H	3.15423000	-3.21971700	0.42414100
H	3.07974800	-3.51170000	2.16011300
H	1.58343800	-3.27792200	1.25324900
H	-2.07682100	2.11419500	-3.13214200
H	-0.81221600	2.39710100	-1.92904400
H	-1.11095200	0.75622700	-2.52946400
H	-4.14590900	2.73888900	-0.10025900
H	-2.65578400	3.59355500	-0.52605600
H	-3.81956500	3.18068100	-1.77354600
H	-4.14121800	0.93949200	-2.61620800
H	-3.29110400	-0.45716700	-1.95500500
H	-4.55358600	0.35491200	-1.00510600
H	-4.66098400	0.81183900	2.90185500
H	-3.74773100	2.15705700	2.22268300
H	-4.81670700	1.18847600	1.18827600
H	-2.78401400	-1.98225000	1.27554000
H	-4.23086300	-1.54645300	2.20060300
H	-4.15493700	-1.19744600	0.47274500
H	-1.26847000	-0.66411800	2.91113600
H	-1.84620300	0.91066800	3.50268100
H	-2.79342800	-0.54377500	3.79224600
C	-0.96020100	-4.42468600	0.00197200
C	-0.78587800	-3.33041800	-1.02815900
H	-1.72275700	-4.18410400	0.74875600
H	-1.27623300	-5.34883700	-0.49856900

H	-0.02359300	-4.63762900	0.52213900
C	0.51806800	-2.87019500	-1.38563200
H	1.36890700	-3.40853900	-0.98832300
H	0.66499300	-2.47780000	-2.39171300
C	-1.87652600	-2.68644600	-1.60787400
C	-3.29735800	-3.14731400	-1.43566600
H	-3.51063400	-3.52425700	-0.43331300
H	-4.02333700	-2.36814400	-1.67454700
H	-1.67831200	-2.08023800	-2.49139700
H	-3.48032600	-3.97376400	-2.13930000

10-ts

Zero-point correction= 0.884580 (Hartree/Particle)

Thermal correction to Energy= 0.933326

Thermal correction to Enthalpy= 0.934271

Thermal correction to Gibbs Free Energy= 0.804430

E(Solv)= -2328.13125863

Pd	-0.16740700	-0.10479700	0.02020400
P	-1.22030900	2.03516000	0.40902500
P	-1.96540800	-1.75667700	0.41032500
C	-3.09835800	1.97727500	0.67011700
C	-4.00894500	1.32136400	-0.35387000
C	-4.73208200	2.14660700	-1.23207100
C	-5.66371700	1.64588100	-2.13754300
C	-5.92914300	0.27927700	-2.15149900
C	-5.25775500	-0.54921800	-1.25665400
C	-4.28381300	-0.06843700	-0.36494700
C	-3.70047200	-1.03636900	0.64665900
C	-0.97057900	3.31327900	-1.02289000
C	-1.16998600	2.52555300	-2.33828200
C	-1.95151400	4.50459200	-1.01538500
C	0.45728200	3.90036800	-1.00590800
C	-0.66455100	2.83420200	2.08851500
C	-1.22587300	1.97762600	3.24386200
C	-1.13458900	4.28751800	2.29630400
C	0.87342700	2.77586800	2.20393800
C	-2.12375000	-2.92112000	-1.12140200
C	-2.03871600	-2.00028300	-2.36148100
C	-3.41832500	-3.75217100	-1.21659100
C	-0.92576300	-3.88962900	-1.15818800
C	-1.83692300	-2.81365300	2.03440200
C	-2.75922000	-4.04703000	2.08817500
C	-0.37731800	-3.27225300	2.21815100
C	-2.18337500	-1.90929500	3.23810900
H	-3.21022000	1.46094800	1.62669200
H	-3.44042300	3.00362000	0.83790500
H	-4.59367800	3.22077500	-1.17843000
H	-6.19773700	2.32306800	-2.79763100
H	-6.67411000	-0.13617400	-2.82374200
H	-5.52597300	-1.60012300	-1.22325300
H	-4.39583300	-1.87268900	0.77362900
H	-3.64504500	-0.54014300	1.61884000
H	-0.44917100	1.70829600	-2.43456000
H	-2.17429700	2.09864600	-2.41343700
H	-1.03174300	3.20489300	-3.18966200
H	-1.90444900	5.09488300	-0.09866400
H	-1.68927700	5.17307800	-1.84553800
H	-2.98426800	4.19786300	-1.17369500
H	0.61822200	4.45014400	-1.94219300
H	0.59991400	4.61340200	-0.18895300
H	1.23416900	3.13843100	-0.93587700
H	-0.98084900	0.91967100	3.11367300
H	-0.76946700	2.30935800	4.18498900
H	-2.30854700	2.07652900	3.36400400
H	-2.21764300	4.40098900	2.18416500
H	-0.88334200	4.59854400	3.31874200
H	-0.64196800	4.98886000	1.61832300
H	1.38530300	3.32833400	1.41352900
H	1.17475400	3.21681300	3.16307200
H	1.22578700	1.73990400	2.18395200
H	-2.06374800	-2.61706300	-3.26973200
H	-2.87479700	-1.29803600	-2.41315500
H	-1.10964200	-1.42108700	-2.36838000
H	-3.53489400	-4.46058200	-0.39515600
H	-4.31264100	-3.13011600	-1.26057200
H	-3.39108400	-4.33505900	-2.14654400
H	-0.90156300	-4.39316700	-2.13331500
H	0.02324900	-3.36163300	-1.03258400
H	-0.99662500	-4.67023000	-0.39473800
H	-2.70055800	-4.49197900	3.09017900
H	-3.80993600	-3.79724600	1.90897700
H	-2.46262000	-4.82176400	1.37641100
H	0.30970000	-2.41931600	2.21775600
H	-0.27630200	-3.78937100	3.18115800
H	-0.06221800	-3.97194700	1.44088300
H	-1.62519200	-0.96911000	3.22150200
H	-3.25091700	-1.68033000	3.30182900
H	-1.91430200	-2.43410000	4.16328100

H	1.75910300	-3.48647300	0.38758700
C	1.92676700	-1.36951100	-2.18343700
C	1.90520200	-0.80442000	-0.77064500
H	2.93327600	-1.30507300	-2.62795200
H	1.60756700	-2.41426000	-2.23975000
H	1.26369000	-0.79348800	-2.83477600
C	1.70791500	0.60511500	-0.60136100
H	1.65260400	1.20521000	-1.50707500
H	2.21813100	1.11394100	0.21580000
C	2.60205900	-1.52337900	0.27965700
C	2.74213900	-3.03020800	0.25766500
H	3.14023100	-3.39648000	-0.69445900
H	3.39058000	-3.37702000	1.06589400
H	2.45190400	-1.11584900	1.27744000
C	6.19445900	0.30038700	0.39717400
C	6.32455000	-0.51630100	-0.75713300
C	7.44856500	-0.45758400	-1.59642800
C	8.43408400	0.44927500	-1.24454900
C	8.32199800	1.27860600	-0.09875900
C	7.21625100	1.21673300	0.72720000
C	4.95063800	-0.07113500	0.97118900
H	7.54261400	-1.08766500	-2.47485000
H	9.32191300	0.52940600	-1.86445500
H	9.12574500	1.97173400	0.12791700
H	7.13349300	1.85002400	1.60484400
H	4.45505900	0.30833900	1.85415400
N	4.38596400	-1.03091300	0.24997900
N	5.18989100	-1.28173600	-0.81753100
H	4.99016200	-2.07132800	-1.41332300

10-ts-iso

Zero-point correction= 0.885154 (Hartree/Particle)

Thermal correction to Energy= 0.933640

Thermal correction to Enthalpy= 0.934584

Thermal correction to Gibbs Free Energy= 0.805712

E(Solv) = -2328.12742014

Pd	-0.16073100	0.05783700	-0.15316400
P	-1.52221400	1.94618500	0.49658600
P	-1.54663800	-1.91722900	0.38513900
C	-3.29993200	1.52675000	1.00477800
C	-4.21264100	0.73797100	0.08155200
C	-5.19602100	1.43436400	-0.64166100
C	-6.14322500	0.79022400	-1.43318800
C	-6.15318600	-0.60106700	-1.48156600
C	-5.21642100	-1.31068400	-0.73527200
C	-4.22538300	-0.67797200	0.03433500
C	-3.33009900	-1.54377200	0.90050100
C	-1.71818300	3.28604600	-0.88685000
C	-1.95665900	2.51091200	-2.20314900
C	-2.89049200	4.26891100	-0.68260500
C	-0.43547500	4.13300300	-1.03162900
C	-0.88701000	2.78852600	2.12559800
C	-1.11597300	1.81255700	3.29983100
C	-1.57880300	4.12259600	2.46913900
C	0.63677800	3.01512800	2.03099400
C	-1.71312800	-3.04773400	-1.17034800
C	-1.96643700	-2.09306900	-2.36084900
C	-2.84365900	-4.09458400	-1.13257600
C	-0.38356300	-3.78610600	-1.41801400
C	-0.98633100	-2.97363500	1.91497000
C	-1.64808000	-4.36078400	2.02842400
C	0.54385100	-3.14977500	1.86869800
C	-1.31230300	-2.18555300	3.20307700
H	-3.17775800	0.97399500	1.93949200
H	-3.79584900	2.46579400	1.27079100
H	-5.25103500	2.51350700	-0.55007600
H	-6.88383400	1.37105300	-1.97501100
H	-6.90097100	-1.13239800	-2.06289800
H	-5.28128500	-2.39360500	-0.71891500
H	-3.83275200	-2.50025700	1.07838900
H	-3.22360900	-1.07243700	1.88077200
H	-1.12021500	1.84692000	-2.44104000
H	-2.86567400	1.90375800	-2.16298900
H	-2.07004000	3.22679700	-3.02786700
H	-2.82329800	4.83089600	0.25042300
H	-2.87685500	4.99836400	-1.50264100
H	-3.86066700	3.77570900	-0.71748300
H	-0.51485000	4.73022900	-1.94917900
H	-0.30935000	4.83571500	-0.20324000
H	0.47035000	3.53052000	-1.10930800
H	-0.70614800	0.82179200	3.08411800
H	-0.59711400	2.19822300	4.18631700
H	-2.17060800	1.70691200	3.56996900
H	-2.66917500	4.03429400	2.51173300
H	-1.24403300	4.44927400	3.46222200
H	-1.32396400	4.92042000	1.76717600
H	0.92380200	3.67279000	1.20799000
H	0.98514500	3.48225100	2.96111700

H	1.16750900	2.06521300	1.91503600
H	-2.02027000	-2.67994400	-3.28741000
H	-2.90709300	-1.54542100	-2.25884700
H	-1.15890900	-1.36144100	-2.46783500
H	-2.72713100	-4.82323400	-0.32914600
H	-3.83050200	-3.64063100	-1.04250400
H	-2.83537700	-4.65071100	-2.07906300
H	-0.40946200	-4.24688400	-2.41390400
H	0.46966900	-3.10367000	-1.39034300
H	-0.21148500	-4.58987700	-0.69596800
H	-1.35799200	-4.81581800	2.98452000
H	-2.74157100	-4.30920200	2.01654800
H	-1.32611100	-5.04402400	1.23836000
H	1.05292200	-2.18093300	1.83064600
H	0.87656700	-3.67234800	2.77492700
H	0.86895300	-3.74588500	1.01340900
H	-0.94276400	-1.15721600	3.16117400
H	-2.38252700	-2.16355400	3.42709900
H	-0.81926200	-2.67640400	4.05140100
H	2.38349700	-2.92742700	-0.33832200
C	1.81361800	-0.75306800	-2.66068200
C	1.88204600	-0.22124700	-1.23318700
H	2.76617600	-0.62239700	-3.19941900
H	1.57461100	-1.81853700	-2.70941300
H	1.04458400	-0.22180100	-3.22889400
C	1.44768400	1.12614300	-0.96781600
H	1.14890600	1.72760900	-1.82508400
H	1.94668000	1.69878700	-0.18644000
C	2.81304000	-0.83461400	-0.30833800
C	3.24479000	-2.27173300	-0.48588200
H	3.63802200	-2.45035400	-1.48980200
H	4.00879900	-2.55760000	0.23952800
H	2.71724600	-0.50469300	0.72182000
C	6.62400000	0.81631100	-0.72765100
C	5.74863100	-0.08655900	-0.03644800
C	6.23557500	-0.91034100	1.00680000
C	7.57523500	-0.81421000	1.31350700
C	8.45350300	0.07234900	0.62365900
C	7.99797900	0.88728200	-0.38618500
C	5.80577800	1.47506700	-1.65608200
H	5.58155000	-1.58107200	1.55266700
H	7.98011400	-1.43066600	2.11061200
H	9.49961200	0.10156400	0.91134600
H	8.66421900	1.56788200	-0.90698000
H	6.01468600	2.24323800	-2.38650300
N	4.57146400	0.97278400	-1.50161100
N	4.49257200	0.03582700	-0.53843600
H	3.70707600	1.24600000	-1.95838600

11

Zero-point correction= 0.886879 (Hartree/Particle)

Thermal correction to Energy= 0.935272

Thermal correction to Enthalpy= 0.936216

Thermal correction to Gibbs Free Energy= 0.808188

E(Solv)= -2328.13979717

Pd	-0.13775500	-0.03257600	0.11792600
P	-1.48897500	1.98252700	0.52164700
P	-1.77783000	-1.87247600	0.31433000
C	-3.36815300	1.74012500	0.62345800
C	-4.10131300	1.04651100	-0.51087700
C	-4.85151500	1.83395300	-1.40109900
C	-5.61281700	1.28560200	-2.42972900
C	-5.67125800	-0.09878800	-2.56546100
C	-4.97820200	-0.89747300	-1.65996500
C	-4.17751000	-0.36263400	-0.63631000
C	-3.58841200	-1.31382800	0.38874100
C	-1.26621000	3.33641900	-0.84729600
C	-1.28361100	2.58484700	-2.19857700
C	-2.35776600	4.42623700	-0.88584600
C	0.09082000	4.05813300	-0.70020000
C	-1.14875100	2.78092300	2.25830800
C	-1.71417000	1.83858000	3.34236500
C	-1.76826000	4.17629600	2.46834300
C	0.37504100	2.85853100	2.49468300
C	-1.70434200	-3.03611300	-1.22668900
C	-1.59777300	-2.09740000	-2.45145000
C	-2.90514400	-3.97989100	-1.43830200
C	-0.42823200	-3.89737400	-1.16331300
C	-1.73612300	-2.94251900	1.94056800
C	-2.55278300	-4.24831800	1.89113800
C	-0.27254700	-3.28404400	2.27653500
C	-2.27786900	-2.09065800	3.10989800
H	-3.50909100	1.16868100	1.54462600
H	-3.82938500	2.71743600	0.79843300
H	-4.87212900	2.90939700	-1.26143700
H	-6.17586800	1.93423400	-3.09443900
H	-6.27791000	-0.55673800	-3.34126700
H	-5.09398000	-1.97401600	-1.72772100

H	-4.21031500	-2.21435200	0.42457500
H	-3.67148400	-0.85301600	1.37541500
H	-0.48682800	1.83743600	-2.25764500
H	-2.23479000	2.07346400	-2.37098800
H	-1.13969700	3.30517500	-3.01498000
H	-2.44298800	4.98332900	0.04875400
H	-2.10063800	5.14823000	-1.67199200
H	-3.33843300	4.02508800	-1.13711200
H	0.26898800	4.65496600	-1.60433400
H	0.10036200	4.75043200	0.14635600
H	0.93052900	3.37070000	-0.58857700
H	-1.35187200	0.81522500	3.21124100
H	-1.37499700	2.18508800	4.32683900
H	-2.80764000	1.82437700	3.36929500
H	-2.84598200	4.19150300	2.27596700
H	-1.62432400	4.47611900	3.51473800
H	-1.29700200	4.94294900	1.84824300
H	0.88934600	3.49310500	1.76967800
H	0.56161300	3.28040600	3.49091800
H	0.82608400	1.86154600	2.46216000
H	-1.48058900	-2.70233600	-3.36056400
H	-2.49058400	-1.47901600	-2.57614900
H	-0.73417500	-1.43029400	-2.37131600
H	-3.00273000	-4.73102700	-0.65339400
H	-3.85354200	-3.44764700	-1.51731700
H	-2.76301200	-4.51744700	-2.38499200
H	-0.27866600	-4.39085900	-2.13255500
H	0.45801600	-3.29260600	-0.95687200
H	-0.49575800	-4.68626900	-0.40782200
H	-2.56578100	-4.69760200	2.89299300
H	-3.59508900	-4.08521400	1.59920100
H	-2.11669500	-4.98815900	1.21496100
H	0.33416800	-2.37627500	2.36230900
H	-0.23193300	-3.81125800	3.23869000
H	0.18063600	-3.94077200	1.52995800
H	-1.80740100	-1.10493300	3.15328900
H	-3.36277900	-1.95875600	3.07122300
H	-2.05342600	-2.60309700	4.05381300
H	1.88519500	-2.97321500	0.78555300
C	2.13660700	-1.02749500	-1.95813700
C	1.98876100	-0.50102200	-0.53549100
H	3.17212600	-0.93411500	-2.32804200
H	1.85960700	-2.08090600	-2.05485700
H	1.49887200	-0.46275300	-2.64373200
C	1.69213000	0.89022700	-0.35440600
H	1.58069400	1.51836800	-1.23690400
H	2.04069500	1.42134000	0.53352600
C	2.91858000	-1.13817600	0.48344500
C	2.90612100	-2.65966400	0.58283500
H	3.23012300	-3.13293300	-0.34901300
H	3.53217600	-3.02179000	1.40497900
H	2.73779800	-0.69858200	1.46943300
C	6.59109100	-0.58960600	-0.14830500
C	6.00138500	0.67369400	-0.42634900
C	6.75950400	1.77964100	-0.84635500
C	8.12195600	1.57648400	-0.97159000
C	8.73247400	0.32377600	-0.69170000
C	7.98783100	-0.76253700	-0.28160500
C	5.51777500	-1.43106600	0.21842500
H	6.30289300	2.74030100	-1.05908400
H	8.74926100	2.40210200	-1.29350200
H	9.80732600	0.22825300	-0.80563500
H	8.45358400	-1.71912700	-0.06823300
H	5.51839600	-2.46869000	0.51130900
N	4.38218200	-0.73362900	0.16831600
N	4.66364000	0.54440800	-0.19455800
H	3.87318500	1.13860100	-0.43668600

12-ts

Zero-point correction= 0.908227 (Hartree/Particle)

Thermal correction to Energy= 0.961275

Thermal correction to Enthalpy= 0.962219

Thermal correction to Gibbs Free Energy= 0.817393

E(Solv)= -2709.98191475

Pd	-0.08672000	-0.26286500	0.18644300
P	-1.42532600	-0.16021900	2.00335400
P	-1.32882300	-1.11431500	-1.80394100
C	-3.22088700	-0.65084200	1.58377300
C	-4.01560900	0.07934100	0.51177600
C	-4.93430500	1.05863000	0.93007600
C	-5.78280400	1.72613000	0.05148600
C	-5.75806800	1.39020000	-1.29946500
C	-4.88669200	0.39426700	-1.73015500
C	-3.99565100	-0.26616100	-0.86558600
C	-3.16984000	-1.39880700	-1.44998700
C	-1.54917000	1.57513700	2.84358500
C	-1.68003500	2.59041100	1.68461900
C	-2.73050800	1.77068500	3.81502300

C	-0.24643700	1.89393300	3.60792500
C	-1.01183300	-1.50677300	3.33567400
C	-1.34181300	-2.89334900	2.73913900
C	-1.76897600	-1.36377400	4.66968600
C	0.50795000	-1.48706300	3.60601700
C	-1.26451800	0.12381100	-3.27736500
C	-1.47357000	1.52392200	-2.65264100
C	-2.30674900	-0.08309900	-4.39233800
C	0.14946200	0.09836200	-3.89715600
C	-0.80194500	-2.87667200	-2.40172700
C	-1.34786800	-3.31242400	-3.77356300
C	0.74190900	-2.93036200	-2.41585900
C	-1.27997600	-3.89916200	-1.34640700
H	-3.11996300	-1.69904800	1.29240500
H	-3.80366200	-0.64411400	2.51220800
H	-5.00829000	1.28463200	1.98848200
H	-6.47188800	2.47770900	0.42682200
H	-6.42623700	1.87334600	-2.00705700
H	-4.91443200	0.09410200	-2.77325200
H	-3.66745400	-1.76407000	-2.35580000
H	-3.16632200	-2.23449500	-0.74435800
H	-0.83050200	2.51340200	0.99875700
H	-2.59610700	2.44606200	1.10511700
H	-1.69959900	3.60803200	2.09856200
H	-2.66325400	1.13154100	4.69744500
H	-2.72708900	2.81097200	4.16734300
H	-3.69981100	1.59789800	3.34582600
H	-0.27061100	2.95003600	3.90960900
H	-0.14946700	1.30296200	4.52409900
H	0.64875300	1.74343700	2.99949500
H	-0.90704200	-3.01318800	1.74161400
H	-0.91079800	-3.66937300	3.38510500
H	-2.41776000	-3.08621100	2.68024200
H	-2.85465900	-1.30233900	4.53578000
H	-1.56907900	-2.24648100	5.29230800
H	-1.44218000	-0.48988100	5.23977200
H	0.86043800	-0.53388400	4.00443200
H	0.75022000	-2.26754700	4.34062700
H	1.07040100	-1.68839300	2.68985200
H	-1.39899800	2.28426300	-3.44273800
H	-2.45361300	1.62869300	-2.17915900
H	-0.70781000	1.73686900	-1.89965500
H	-2.20567100	-1.04766100	-4.89455400
H	-3.33197900	0.00362500	-4.02559500
H	-2.17266500	0.69693400	-5.15454600
H	0.25212800	0.95765800	-4.57433400
H	0.93148200	0.17148600	-3.13494900
H	0.32192900	-0.80188500	-4.49567700
H	-1.08096700	-4.36310300	-3.95450400
H	-2.44002800	-3.24097700	-3.83138900
H	-0.92178300	-2.72723600	-4.59302800
H	1.15657500	-2.65458500	-1.44151100
H	1.06466600	-3.95310800	-2.65611200
H	1.18194800	-2.26000300	-3.15623300
H	-1.00205600	-3.59570200	-0.33164000
H	-2.36162200	-4.06523500	-1.37776300
H	-0.80009600	-4.86658700	-1.54436800
P	2.91114600	0.67248000	0.02326500
O	1.91787900	0.03542600	-0.94018500
O	2.29258400	0.88750300	1.43595400
H	1.07270300	0.41531300	1.28156100
O	4.29442300	-0.15079700	0.11235300
O	3.54743900	2.05276100	-0.51299600
C	2.77917100	3.26864000	-0.41822100
H	2.52863000	3.45241000	0.63302900
H	1.84384800	3.15564700	-0.98223400
C	3.61871000	4.40088700	-0.99232300
H	3.87924400	4.15601900	-2.03027600
H	4.56255900	4.45611200	-0.43443000
C	2.89368800	5.75207400	-0.93638900
H	2.62789500	5.98186800	0.10515500
H	1.94400900	5.68105900	-1.48566000
C	3.73039100	6.89908100	-1.51324300
H	3.98321500	6.71450100	-2.56443000
H	3.19039000	7.85133100	-1.46155800
H	4.67145000	7.01787700	-0.96259400
C	4.24266900	-1.57853400	0.29960300
H	3.69667000	-2.03166500	-0.53619200
H	3.70243300	-1.80471700	1.22878900
C	5.67244200	-2.09581800	0.36886100
H	6.19326100	-1.81314500	-0.55531800
H	6.19184900	-1.58823300	1.19200400
C	5.73514800	-3.61643700	0.56448100
H	5.20178200	-4.11353200	-0.25825500
H	5.19978600	-3.88949600	1.48505300
C	7.17094900	-4.14748700	0.63453200
H	7.18755500	-5.23436100	0.77422000
H	7.72194500	-3.92007300	-0.28612000

H	7.71986100	-3.69451100	1.46905000

13

Zero-point correction= 0.912443 (Hartree/Particle)

Thermal correction to Energy= 0.965236

Thermal correction to Enthalpy= 0.966180

Thermal correction to Gibbs Free Energy=0.823148

E(Solv)= -2711.23539894

Pd	-0.36198900	0.34059000	0.54218800
P	-2.07187300	1.75899900	0.05777900
P	-1.36448500	-1.89104400	0.81313600
C	-3.80217100	0.99508700	-0.03948200
C	-4.12723900	-0.12969900	-1.00631800
C	-4.86598000	0.18659400	-2.15912400
C	-5.30066300	-0.78021600	-3.06164700
C	-5.03604800	-2.12070300	-2.79583800
C	-4.34956800	-2.45808400	-1.63248500
C	-3.86636400	-1.49541700	-0.72999600
C	-3.23848500	-1.98330500	0.56343200
C	-1.73500400	2.59189300	-1.64565100
C	-1.23881200	1.46590200	-2.58394900
C	-2.96959200	3.26638800	-2.27734400
C	-0.61474600	3.64614600	-1.53441300
C	-2.35946500	3.08475400	1.43649300
C	-2.98938800	2.38148500	2.66031700
C	-3.28978600	4.23691700	1.00598200
C	-1.01059800	3.67436900	1.90139000
C	-0.61737000	-3.18849600	-0.39584100
C	-0.47801200	-2.46667100	-1.75720200
C	-1.47714100	-4.45422000	-0.58436800
C	0.79003500	-3.62377000	0.06500000
C	-1.21920300	-2.49654800	2.64088600
C	-1.63161200	-3.96445100	2.86151600
C	0.22975200	-2.28785700	3.13463800
C	-2.12598400	-1.59985200	3.51391800
H	-3.97686600	0.64676300	0.98218800
H	-4.50929800	1.81449200	-0.20879100
H	-5.14162000	1.22126000	-2.33541900
H	-5.86431400	-0.48720500	-3.94285800
H	-5.38609400	-2.90092500	-3.46582800
H	-4.21283200	-3.50828700	-1.39639000
H	-3.56151400	-3.01445600	0.73988200
H	-3.64029700	-1.39340300	1.39262500
H	-0.29058400	1.04049800	-2.24014600
H	-1.97096100	0.65861400	-2.68346500
H	-1.07574000	1.89113600	-3.58358800
H	-3.40604500	4.04623600	-1.64926900
H	-2.66016100	3.74115100	-3.21722200
H	-3.74831800	2.54681000	-2.52874100
H	-0.33481900	3.95540400	-2.54951500
H	-0.93579600	4.54500200	-0.99844100
H	0.28184300	3.24510800	-1.05515000
H	-2.41801500	1.49632300	2.95612200
H	-2.98058300	3.07742000	3.50863000
H	-4.03132200	2.08997700	2.49563300
H	-4.25378900	3.88589300	0.62218000
H	-3.50126500	4.86672800	1.87999400
H	-2.83105900	4.87952800	0.24978000
H	-0.46577000	4.17451200	1.09857100
H	-1.20645100	4.41805800	2.68542500
H	-0.35868500	2.90100800	2.31549900
H	-0.10219700	-3.18426400	-2.49941900
H	-1.43673500	-2.08561800	-2.12413300
H	0.23310600	-1.63669900	-1.70688200
H	-1.68223500	-4.97792800	0.35285700
H	-2.42691700	-4.24345900	-1.07756200
H	-0.92918300	-5.15045900	-1.23285200
H	1.25476400	-4.19119000	-0.75253400
H	1.43488100	-2.77439200	0.29492800
H	0.75015200	-4.28915600	0.93353100
H	-1.61139300	-4.18340900	3.93772600
H	-2.64699500	-4.17723500	2.50817700
H	-0.94470400	-4.66379700	2.37780600
H	0.55669700	-1.25255600	3.01302400
H	0.27478100	-2.54743300	4.20131200
H	0.95122500	-2.91313400	2.60833800
H	-1.92495700	-0.53746700	3.34322100
H	-3.19357600	-1.78636400	3.35817900
H	-1.91534600	-1.80825100	4.57057800
P	2.64898200	-0.09639200	-0.17559700
O	1.54995400	-0.52210000	0.82542800
O	2.28349300	0.20799200	-1.59688800
H	0.35448300	1.72968100	0.41662400
O	3.51369500	1.12810600	0.49429200
O	3.70826500	-1.32745200	-0.01857400
C	3.52012600	2.43355200	-0.09789600
H	2.60662700	2.96790700	0.19976400
H	3.51686500	2.34436500	-1.18974200

C	4.75248800	3.18240500	0.39535000
H	4.74175200	3.19871800	1.49348300
H	5.64925500	2.62095600	0.09972800
C	4.82890700	4.61474300	-0.14911200
H	3.92318200	5.16540800	0.14374000
H	4.82745200	4.58878800	-1.24809600
C	6.06576000	5.37561300	0.34071700
H	6.09428300	6.39500600	-0.06145500
H	6.07815200	5.44736300	1.43526600
H	6.98884200	4.86928200	0.03289300
C	4.89631100	-1.31413100	-0.82222500
H	5.52197400	-0.46039700	-0.52721800
H	4.62294700	-1.18875900	-1.87761300
C	5.63982300	-2.62498600	-0.60088400
H	5.85277700	-2.73250100	0.47105800
H	4.97660000	-3.45656700	-0.87451300
C	6.94326800	-2.70744700	-1.40584600
H	7.59527600	-1.86551600	-1.13272300
H	6.72086500	-2.58585400	-2.47549700
C	7.69636200	-4.02441300	-1.18904800
H	8.62263300	-4.05671400	-1.77436400
H	7.96310900	-4.15829800	-0.13347000
H	7.08323400	-4.88416500	-1.48620000

14-ts

Zero-point correction= 0.759767 (Hartree/Particle)

Thermal correction to Energy= 0.801255

Thermal correction to Enthalpy= 0.802199

Thermal correction to Gibbs Free Energy= 0.690055

E(Solv)= -1948.13224241

Pd	-0.38672400	-0.95014200	-0.42840500
P	1.87573800	-0.87299200	0.29051800
P	-1.42223400	1.14402000	0.39677800
C	2.41902900	0.70793400	1.16181100
C	2.28664700	2.04894200	0.46538200
C	3.44810200	2.64209200	-0.05691600
C	3.44135300	3.90333700	-0.64671600
C	2.25204400	4.62601100	-0.68586600
C	1.10299200	4.07526700	-0.12390500
C	1.07977200	2.78938800	0.44183600
C	-0.17608000	2.34114500	1.16823400
C	3.02898100	-1.08248400	-1.24037600
C	2.47718700	-0.14719800	-2.34153300
C	4.50257100	-0.71199000	-0.96949800
C	2.99454700	-2.53169000	-1.76790600
C	2.24329000	-2.21333300	1.63207000
C	1.61779200	-1.75350500	2.96736400
C	3.74991000	-2.45165100	1.86334600
C	1.57008200	-3.54640100	1.24234800
C	-2.27993100	2.15225100	-1.00508400
C	-1.35243800	2.04193800	-2.23726100
C	-2.50014900	3.64545600	-0.68725500
C	-3.65240300	1.54417800	-1.35769700
C	-2.62074900	0.89856200	1.89485900
C	-3.41729000	2.16063400	2.28044500
C	-3.59749300	-0.24851700	1.57523500
C	-1.79184600	0.45668700	3.12138700
H	1.83656600	0.71197900	2.08685500
H	3.46273300	0.56014600	1.45961200
H	4.39420400	2.11887000	0.03554700
H	4.36177100	4.32509900	-1.03902700
H	2.22272800	5.62438700	-1.11185100
H	0.20714100	4.68498400	-0.08249600
H	-0.73946200	3.22974500	1.46954800
H	0.11588100	1.84471200	2.09671700
H	1.44978000	-0.40711700	-2.61693600
H	2.49348700	0.90343700	-2.04088600
H	3.10226600	-0.24815900	-3.23767900
H	4.96668900	-1.33130100	-0.20052100
H	5.07087500	-0.86376600	-1.89526100
H	4.62385700	0.33436200	-0.69253300
H	3.53783400	-2.56421100	-2.72006600
H	3.48599500	-3.23695500	-1.09245900
H	1.97933600	-2.89039200	-1.96415800
H	0.56173800	-1.49050100	2.85773000
H	1.67485700	-2.58245000	3.68285800
H	2.14699000	-0.90784400	3.41588200
H	4.29471500	-1.53025700	2.09304200
H	3.86888400	-3.11846700	2.72616600
H	4.23352400	-2.93874100	1.01305200
H	1.92511600	-3.94384900	0.28885700
H	1.79627800	-4.29350600	2.01302700
H	0.48161200	-3.44370100	1.18724400
H	-1.78596300	2.61385100	-3.06755400
H	-0.35467200	2.44577600	-2.04096200
H	-1.23807700	1.00424000	-2.56939300
H	-3.09908800	3.80963100	0.21038600
H	-1.56374900	4.19212800	-0.58284000

H	-3.04114000	4.10077700	-1.52629300
H	-4.00474000	1.99274200	-2.29500200
H	-3.61374700	0.46368900	-1.50189600
H	-4.40669300	1.75676300	-0.59520200
H	-3.97605100	1.95469800	3.20213600
H	-2.77473700	3.02390800	2.48195600
H	-4.14928200	2.44308900	1.51968100
H	-3.06437900	-1.18316600	1.37936700
H	-4.25152700	-0.41127900	2.44131800
H	-4.23697000	-0.04281900	0.71619200
H	-1.13523700	-0.38693700	2.88785500
H	-1.19042600	1.26459600	3.54784600
H	-2.48093600	0.12478500	3.90731600
H	0.32373100	-2.26037600	-0.93296100
C	-2.92527300	-3.84959500	-0.10970500
C	-3.31015500	-2.68047500	-0.98573500
H	-2.50677100	-4.67614100	-0.69832300
H	-2.16648200	-3.56474400	0.63055200
H	-3.79507600	-4.23796400	0.42612700
C	-4.59546200	-2.32174700	-1.15559500
H	-5.40340300	-2.85631000	-0.66493600
H	-4.88404500	-1.50342100	-1.80961700
C	-2.26126500	-1.97389100	-1.74028600
C	-1.04423600	-2.54422800	-2.08903400
H	-0.83666300	-3.58083600	-1.83975100
H	-0.49137400	-2.15044200	-2.93681800
H	-2.57833400	-1.10435500	-2.30797700

15-ts

Zero-point correction=1.024613 (Hartree/Particle)

Thermal correction to Energy=1.084476

Thermal correction to Enthalpy=1.085420

Thermal correction to Gibbs Free Energy=0.925103

E(Solv) = -2906.59529205

Pd	2.49758200	0.71529200	0.46076700
P	2.55791800	-1.49851300	-0.39530400
P	-2.31090500	-2.24764000	-1.61819500
C	1.02320800	-2.63316000	-0.27914400
C	0.20109100	-2.72704400	0.99564700
C	0.68345400	-3.48006400	2.07904000
C	-0.02729800	-3.63458300	3.26695600
C	-1.27574900	-3.03021300	3.39414900
C	-1.79232900	-2.31828700	2.31477200
C	-1.09593800	-2.16510300	1.10143200
C	-1.80008900	-1.39034000	0.00468300
C	3.99864100	-2.56374900	0.34503100
C	4.02491000	-2.26758900	1.86313300
C	3.86677800	-4.08469900	0.12169700
C	5.36202600	-2.12235700	-0.22618100
C	2.73610200	-1.40427800	-2.32253800
C	1.43269500	-0.77440800	-2.86570600
C	2.95890800	-2.76085900	-3.01718000
C	3.87675400	-0.43589000	-2.70366300
C	-3.23577000	-0.75183700	-2.43756500
C	-2.11888600	0.14769200	-3.01640700
C	-4.11618400	0.11374600	-1.51388500
C	-4.09191600	-1.24511500	-3.62069200
C	-3.57234000	-3.61810800	-1.11638100
C	-4.80772200	-3.15968400	-0.32380000
C	-4.02112600	-4.35698700	-2.39771400
C	-2.78388600	-4.64931700	-0.27494600
H	0.37558800	-2.29401500	-1.08562800
H	1.35079300	-3.63775700	-0.56732600
H	1.64215200	-3.97694000	1.98709700
H	0.39125000	-4.22450400	4.07796500
H	-1.85014500	-3.12568700	4.31180500
H	-2.78216400	-1.87537400	2.40058200
H	-1.15502800	-0.56652600	-0.31736400
H	-2.68106700	-0.92106500	0.44974100
H	4.25943600	-1.21648900	2.05045800
H	3.07443400	-2.48015500	2.35664700
H	4.79960800	-2.88479300	2.33914500
H	2.99117500	-4.51688500	0.61173400
H	3.82114300	-4.35065800	-0.93784200
H	4.74863700	-4.58318900	0.54611600
H	6.15967500	-2.58574300	0.37013100
H	5.50546500	-2.44572000	-1.26119900
H	5.49861400	-1.03939900	-0.17542000
H	1.16504900	0.13693800	-2.31973000
H	1.58586900	-0.50049500	-3.91810500
H	0.58089600	-1.45884900	-2.83644100
H	2.18382200	-3.49192900	-2.76435100
H	2.92631600	-2.61890400	-4.10607300
H	3.93453000	-3.19547900	-2.77897700
H	4.86374600	-0.78631200	-2.39814800
H	3.89278300	-0.32231700	-3.79604100
H	3.71202500	0.55162100	-2.26230200
H	-2.57520200	1.00140100	-3.53728600

H	-1.46387900	0.55806900	-2.24136500
H	-1.50010500	-0.39265500	-3.74046000
H	-4.89894700	-0.46426800	-1.01410600
H	-3.53113500	0.63535400	-0.75089600
H	-4.61633000	0.88640500	-2.11530700
H	-4.39110900	-0.38524500	-4.23565100
H	-3.54315800	-1.93910400	-4.26762600
H	-5.01091800	-1.73706200	-3.28803800
H	-5.38867800	-4.03389900	0.00377500
H	-4.53397600	-2.59574100	0.57465900
H	-5.47507700	-2.53638500	-0.92749600
H	-3.16006900	-4.67793300	-2.99519500
H	-4.58498800	-5.25721700	-2.11687400
H	-4.66938300	-3.75411700	-3.03631100
H	-1.86301500	-4.96266500	-0.78075200
H	-2.51634500	-4.27196000	0.71302800
H	-3.40469900	-5.54489300	-0.13337300
C	3.57410100	3.60227400	-0.35288900
C	3.70187700	2.58685200	0.76736300
H	2.54186400	3.94731300	-0.48001300
H	4.19266500	4.48922100	-0.15147000
H	3.90134400	3.17565400	-1.30599900
C	4.46406200	1.38249800	0.58534600
H	5.06869400	1.29863600	-0.31685900
H	4.93246400	0.92811200	1.46019400
C	3.21673200	2.90994200	2.06456000
C	2.45688400	4.02673200	2.42805500
H	3.42109200	2.17134000	2.84141700
P	-0.51400900	2.31668900	0.86049300
O	0.27006600	1.04003800	0.71668000
O	-0.03132200	3.37039500	1.89916700
H	1.09640100	3.57562500	2.11356200
O	-0.65589900	2.99900600	-0.59307900
O	-2.06882400	2.04679100	1.22812700
C	-1.36457600	4.24867000	-0.75394200
H	-0.95959800	4.98274300	-0.04777500
H	-2.42204800	4.08600300	-0.51520400
C	-1.19140400	4.71197300	-2.19290200
H	-0.11950000	4.82698500	-2.40186100
H	-1.56067700	3.92541700	-2.86399800
C	-1.92542400	6.03028000	-2.47389800
H	-1.55820500	6.80697900	-1.78831600
H	-2.99497600	5.90780300	-2.25195300
C	-1.75673400	6.50662400	-3.92058100
H	-2.29014600	7.44795100	-4.09425500
H	-0.69972400	6.67175000	-4.16212100
H	-2.14637700	5.76569400	-4.62920100
C	-2.41688500	1.66070600	2.57901100
H	-1.99580500	0.66874700	2.78140100
H	-1.97261400	2.37837600	3.27732000
C	-3.93396400	1.64994900	2.69767600
H	-4.34752300	0.95288700	1.95650200
H	-4.31560900	2.64643300	2.43876000
C	-4.40480000	1.25739800	4.10491800
H	-4.00574500	0.26566100	4.36090200
H	-3.97986000	1.95538500	4.83971800
C	-5.93129300	1.24305300	4.23827400
H	-6.38366200	0.52872800	3.53953600
H	-6.23955200	0.96029200	5.25108400
H	-6.35650900	2.23088700	4.02312200
H	2.35821500	4.23887800	3.49167300
H	2.48836400	4.90938900	1.78890000

16

Zero-point correction= 0.323415 (Hartree/Particle)
Thermal correction to Energy= 0.340444
Thermal correction to Enthalpy=0.341388
Thermal correction to Gibbs Free Energy=0.278861
E(Solv)= -642.663413811

C	-1.92415000	-0.80823800	-0.37010900
C	-1.90084300	0.46120300	0.26582100
C	-3.07533600	1.12941000	0.64948600
C	-4.27249100	0.48879800	0.37625200
C	-4.31798300	-0.77803500	-0.25842000
C	-3.15953400	-1.43375400	-0.63420400
C	-0.55827900	-1.13683000	-0.58318900
H	-3.04710400	2.09980400	1.13491300
H	-5.20507400	0.97054900	0.65574400
H	-5.28346300	-1.23631600	-0.45008500
H	-3.19664200	-2.40402500	-1.12035300
H	-0.11319500	-2.01103300	-1.03217200
N	0.21852000	-0.16472800	-0.12687600
N	-0.58611800	0.80254600	0.38353100
H	-0.14702900	1.60846300	0.80389700
B	1.86672200	0.02693200	-0.16114700
C	2.18605300	0.95647800	-1.47871900
C	1.65304400	2.39913500	-1.51922400
H	1.84731700	0.43419200	-2.38845000

H	3.28246900	1.00101700	-1.56880900
H	0.55419000	2.43380900	-1.50864100
H	2.00799000	2.98859100	-0.66351300
H	1.97070800	2.93220400	-2.42542000
C	2.27382400	0.74828200	1.25494100
C	2.00251500	-0.04196900	2.54761100
H	1.83049100	1.75694800	1.36274800
H	3.35214000	0.95912300	1.19457300
H	2.32575700	0.49976100	3.44678200
H	0.93432200	-0.26803100	2.67457100
H	2.52804400	-1.00398100	2.54099700
C	2.46184700	-1.48869200	-0.33455900
C	3.99688600	-1.57674300	-0.25817000
H	2.15575000	-1.91334300	-1.30651600
H	2.04415000	-2.16869800	0.42626800
H	4.35881900	-2.60363100	-0.40172800
H	4.47473700	-0.95487600	-1.02499200
H	4.37188200	-1.23418200	0.71424000

16-iso

Zero-point correction= 0.323830 (Hartree/Particle)

Thermal correction to Energy= 0.340628

Thermal correction to Enthalpy= 0.341572

Thermal correction to Gibbs Free Energy= 0.279969

E(Solv)=-642.65639320

C	-2.16361500	1.09712000	0.09891600
C	-1.21125000	0.03157100	-0.03223400
C	-1.64976700	-1.29484300	-0.27117900
C	-3.00603500	-1.50778300	-0.37349200
C	-3.95582400	-0.45179300	-0.24518000
C	-3.55359700	0.84168600	-0.01072700
C	-1.39379200	2.24407100	0.32367800
H	-0.93989900	-2.10512800	-0.36575600
H	-3.36890200	-2.51510300	-0.55714100
H	-5.01334300	-0.68205500	-0.33494000
H	-4.27358500	1.64855600	0.08888800
H	-1.66094200	3.27935600	0.47538000
N	-0.11378700	1.84582700	0.31685500
N	0.04889500	0.52421100	0.10484300
B	1.60251700	-0.11983200	0.16246400
C	1.81762800	-0.66103300	1.69580600
C	0.96761300	-1.84490100	2.18580500
H	1.69014900	0.17491500	2.40541300
H	2.87781900	-0.94573400	1.78819200

H	-0.10501900	-1.60993100	2.17958200
H	1.10842800	-2.73146200	1.55450700
H	1.22132300	-2.13957800	3.21336000
C	1.71243700	-1.28069300	-0.98125000
C	1.43789900	-0.86155300	-2.43627700
H	1.08942200	-2.15678500	-0.74580000
H	2.74019000	-1.67138400	-0.92181300
H	1.57909300	-1.68964800	-3.14409700
H	0.40966800	-0.49844900	-2.56853300
H	2.10449200	-0.04942600	-2.75296100
C	2.59305000	1.16617000	-0.14102000
C	4.07554200	0.79094600	-0.32066600
H	2.56338800	1.90121100	0.68976900
H	2.28150500	1.71873400	-1.04459000
H	4.70997800	1.67510100	-0.46726000
H	4.46126500	0.25355600	0.55356300
H	4.21718800	0.14009500	-1.19086200
H	0.72769500	2.39826500	0.42781200

17-ts

Zero-point correction=1.070491 (Hartree/Particle)

Thermal correction to Energy= 1.130131

Thermal correction to Enthalpy=1.131075

Thermal correction to Gibbs Free Energy= 0.975315

E(Solv)= -2590.33846816

Pd	1.31431100	-0.31721700	0.38428400
P	3.38013700	-1.51816200	0.60166100
P	1.95944200	2.03072600	0.07499900
C	4.94907800	-0.46026800	0.46583300
C	5.24956800	0.40022500	-0.74797900
C	6.20204800	-0.06210400	-1.67235700
C	6.65074100	0.71082100	-2.73988400
C	6.18144100	2.01438700	-2.87524100
C	5.26589900	2.50375900	-1.94773900
C	4.75581400	1.72013400	-0.89812000
C	3.82517800	2.38309400	0.09979400
C	3.62069600	-2.92429700	-0.70233200
C	3.18323800	-2.31631800	-2.05484900
C	5.06097800	-3.45706400	-0.84536100
C	2.70935000	-4.12935500	-0.39091400
C	3.64430800	-2.21448800	2.39608300
C	3.84214200	-1.01494400	3.35159700
C	4.85179000	-3.16081400	2.54684600
C	2.37383000	-2.95029300	2.86825400

C	1.34937200	2.72495600	-1.61859600
C	1.62932900	1.61254600	-2.65552000
C	2.02576500	4.02564700	-2.09437000
C	-0.17087900	2.96772800	-1.57180100
C	1.39402400	3.18903200	1.51772900
C	1.55572200	4.69717400	1.24891000
C	-0.08132000	2.88626600	1.84817600
C	2.22318600	2.82453100	2.77037400
H	4.88613500	0.19004500	1.34157900
H	5.80478500	-1.12079800	0.64047000
H	6.63989700	-1.04392800	-1.52756300
H	7.38607700	0.30872900	-3.43094200
H	6.54351700	2.65685600	-3.67277900
H	4.96234400	3.54299300	-2.01786800
H	3.95974500	3.46767900	0.03283800
H	4.12738400	2.10176500	1.11274400
H	2.14179700	-1.98155700	-2.02677200
H	3.80464200	-1.46362400	-2.34374700
H	3.27535200	-3.07883900	-2.83971000
H	5.76299800	-2.68835400	-1.16670500
H	5.44277000	-3.90927100	0.07178900
H	5.06715000	-4.23634500	-1.61876900
H	2.74746700	-4.82379900	-1.24034000
H	3.04236000	-4.68405600	0.49134500
H	1.66608700	-3.84451700	-0.24890900
H	3.07250400	-0.24967400	3.20716300
H	3.76252500	-1.37121100	4.38638800
H	4.82614800	-0.54750800	3.25060600
H	5.78556400	-2.72394700	2.17813900
H	4.99809300	-3.38141100	3.61252200
H	4.69442300	-4.11727600	2.04084900
H	2.10913600	-3.79978000	2.23491000
H	2.54376800	-3.33680400	3.88162800
H	1.51860800	-2.27115500	2.90890200
H	1.28088900	1.94481400	-3.64252600
H	2.69502000	1.38012700	-2.73834000
H	1.09797500	0.68885000	-2.40229100
H	1.91827600	4.84865000	-1.38501000
H	3.08503700	3.88886600	-2.30872700
H	1.54909400	4.34129400	-3.03181300
H	-0.53404400	3.13319700	-2.59453400
H	-0.71506800	2.11677200	-1.16004200
H	-0.43214900	3.85739000	-0.99114200
H	1.31900700	5.25036500	2.16744800
H	2.57700700	4.97119900	0.96260100
H	0.87210900	5.05523300	0.47467900
H	-0.21631700	1.83255700	2.10573500
H	-0.38728800	3.48915900	2.71320100
H	-0.76151400	3.12007800	1.02788300
H	2.21276200	1.74674200	2.96547100
H	3.26324500	3.15887400	2.70480100
H	1.78072000	3.32002900	3.64372000
C	-1.38035600	-0.89786100	2.24303000
C	-0.91352200	-0.98511800	0.80369800
H	-1.44893300	0.13334500	2.60200600
H	-2.38504700	-1.32976000	2.33711700
H	-0.71205700	-1.44871200	2.91141900
C	0.01094400	-1.99955500	0.41376000
H	0.22651700	-2.78895400	1.12636100
H	-0.00213100	-2.33224600	-0.62539200
C	-1.53888100	-0.19932500	-0.19086800
C	-2.64055300	0.67963800	0.00153700
C	-6.18975700	-2.27643700	-1.40281600
C	-4.89875000	-1.98625400	-0.87112500
C	-3.82287100	-2.88190400	-1.06930300
C	-4.06556600	-4.02996900	-1.80202100
C	-5.34834000	-4.32008400	-2.33757900
C	-6.41145600	-3.45830600	-2.14047100
C	-6.97403400	-1.17808900	-0.99433700
H	-2.84332300	-2.68812600	-0.64292700
H	-3.25628400	-4.73793800	-1.96619600
H	-5.49135200	-5.23793500	-2.90166800
H	-7.39611500	-3.68683200	-2.54071100
H	-8.02029500	-0.97570800	-1.15689600
N	-6.21713400	-0.32600300	-0.29487300
N	-4.94238100	-0.80762300	-0.19252000
H	-3.72783500	-0.02674000	-0.06361800
H	-1.25495600	-0.42492400	-1.21980200
H	-2.81551400	1.40217600	-0.79681100
H	-2.74824200	1.11759000	0.99501700
B	-6.74510000	1.03295300	0.46935000
C	-6.77214300	0.69607500	2.08312900
C	-5.44128800	0.48565000	2.82430800
H	-7.40659700	-0.18954700	2.25574700
H	-7.30268000	1.52491700	2.57928400
H	-4.88121400	-0.35972800	2.40492100
H	-4.79644100	1.37248000	2.75197000
H	-5.58622100	0.28225500	3.89599400

C	-5.74508200	2.27592700	0.08687400
C	-5.60272600	2.60924000	-1.40837000
H	-4.74162700	2.13110900	0.51011800
H	-6.12824400	3.16944700	0.60515800
H	-4.93088100	3.46131900	-1.59511500
H	-5.20902800	1.75431200	-1.97584900
H	-6.57189100	2.86200200	-1.85679800
C	-8.28232100	1.28657900	-0.07427000
C	-8.92559100	2.60199900	0.40033900
H	-8.94411000	0.46344300	0.24691800
H	-8.32357900	1.27916700	-1.17680700
H	-9.96373500	2.70551500	0.05318400
H	-8.94370300	2.67171000	1.49532100
H	-8.37440000	3.47675200	0.03289200

18-ts

Zero-point correction=1.076000 (Hartree/Particle)

Thermal correction to Energy=1.135243

Thermal correction to Enthalpy=1.136187

Thermal correction to Gibbs Free Energy= 0.984666

E(solv)= -2590.34543989

Pd	-0.70881500	0.00574500	-0.28228100
P	-1.86566500	1.97853000	0.46868300
P	-2.22697200	-1.85670700	0.21346100
C	-3.63640200	1.67883700	1.09524600
C	-4.69462200	0.98389200	0.25661100
C	-5.69263200	1.77133500	-0.34358300
C	-6.77144500	1.22414000	-1.03291300
C	-6.90322900	-0.16025900	-1.09549800
C	-5.95156400	-0.95819700	-0.46667900
C	-4.83073400	-0.42542000	0.19311200
C	-3.91610100	-1.38216200	0.93208600
C	-2.06542800	3.33178200	-0.90275500
C	-2.39332200	2.56693400	-2.20504700
C	-3.18556900	4.36263100	-0.65119200
C	-0.75696600	4.12474000	-1.10751400
C	-1.10996700	2.78221800	2.06438500
C	-1.28967600	1.78678900	3.23261900
C	-1.74667000	4.12691000	2.46882100
C	0.40896600	2.97813300	1.88211900
C	-2.65267100	-2.86517800	-1.37747600
C	-2.96952500	-1.81638200	-2.46965300
C	-3.83761500	-3.84448300	-1.27215900
C	-1.41248800	-3.65702600	-1.83122500
C	-1.61253200	-3.04113800	1.62058300
C	-2.39603000	-4.36065800	1.75631500
C	-0.12230400	-3.36340400	1.39580100
C	-1.71044300	-2.27904300	2.96185800
H	-3.47999100	1.09961400	2.00877700
H	-4.03414100	2.64707200	1.41563500
H	-5.64850100	2.84874200	-0.22909700
H	-7.51544300	1.87557200	-1.48264300
H	-7.75162300	-0.61744400	-1.59685900
H	-6.10054600	-2.03306700	-0.45775400
H	-4.46805700	-2.30561400	1.13681100
H	-3.66945200	-0.95399400	1.90724200
H	-1.58844400	1.87930400	-2.48067000
H	-3.31709300	1.98627100	-2.11744200
H	-2.52593300	3.28627700	-3.02453500
H	-4.17694100	3.91389200	-0.68858100
H	-3.07974400	4.89121600	0.29820800
H	-3.14856000	5.11644000	-1.44878000
H	-0.84821900	4.71352500	-2.02983100
H	-0.57310300	4.83073900	-0.29267400
H	0.12167400	3.48777500	-1.21110300
H	-0.92929800	0.78808700	2.96909100
H	-0.69221000	2.13318000	4.08497300
H	-2.32587500	1.71127000	3.57726200
H	-2.83476500	4.07041300	2.57985700
H	-1.33962400	4.42938900	3.44264500
H	-1.51237600	4.92776600	1.76225000
H	0.66337900	3.63266700	1.04672200
H	0.81580800	3.43637800	2.79288500
H	0.92109300	2.02447500	1.74308900
H	-3.15960000	-2.33410900	-3.41976400
H	-3.85526900	-1.22204000	-2.23118900
H	-2.12979700	-1.13019700	-2.61845800
H	-3.66518400	-4.64525100	-0.55103600
H	-4.77242000	-3.34684400	-1.01251800
H	-3.98956900	-4.31652400	-2.25214900
H	-1.59303200	-4.06090900	-2.83629300
H	-0.52729300	-3.01914600	-1.88354400
H	-1.19490800	-4.50578100	-1.17586400
H	-2.05897500	-4.88257600	2.66179200
H	-3.47607700	-4.20756900	1.85577100
H	-2.21910000	-5.03552900	0.91419000
H	0.48392400	-2.45424600	1.36733400
H	0.24254800	-3.98072900	2.22682300

H	0.05403800	-3.92208800	0.47451100
H	-1.24771200	-1.28932500	2.90390100
H	-2.74096900	-2.16667000	3.31274600
H	-1.16933000	-2.84607600	3.72950900
C	0.93399400	-0.59114700	-3.05182400
C	1.23751900	-0.35529800	-1.57972900
H	1.80371700	-0.34229700	-3.67164100
H	0.65393100	-1.62610500	-3.26978300
H	0.10884800	0.04957500	-3.37882600
C	1.02124000	0.95475100	-1.04529400
H	1.63959000	1.27636800	-0.21061800
H	0.81433400	1.74011800	-1.76780200
C	2.01427100	-1.28614900	-0.83590500
C	2.32757700	-2.67383400	-1.31809000
H	3.13578100	-3.10305000	-0.72692500
H	1.44668800	-3.31868700	-1.21860300
H	2.10373500	-1.11556200	0.22556300
H	2.62167600	-2.68316400	-2.37082500
C	5.49446400	1.09360700	-1.49814800
C	4.64616300	0.02079700	-1.90414100
C	4.50060800	-0.28211200	-3.27814000
C	5.17335000	0.50351000	-4.19618900
C	6.00080500	1.58478700	-3.79134700
C	6.17409400	1.88043300	-2.45276500
C	5.43744100	1.04457300	-0.09233400
H	3.90511100	-1.12816300	-3.60568200
H	5.08068800	0.28078000	-5.25642500
H	6.51366000	2.17179000	-4.54854400
H	6.82540800	2.69296800	-2.14026500
H	5.96357800	1.63588500	0.63881000
N	4.62905000	0.04607100	0.28717900
N	4.12779900	-0.60549100	-0.81029800
B	4.43216500	-0.46228200	1.84981000
C	4.36748400	-2.10418200	1.84042900
C	5.57885700	-2.84519900	1.24761000
H	3.45474200	-2.47595100	1.34503000
H	4.25666500	-2.42312300	2.88919200
H	5.48225400	-3.93876500	1.32024400
H	5.71799500	-2.59797600	0.18789900
H	6.50493200	-2.57010900	1.76757500
C	5.75504400	0.03521700	2.71449700
C	5.80228900	1.45917100	3.30714600
H	6.67727700	-0.13072600	2.13499500
H	5.84530800	-0.65981300	3.56473000
H	6.73115200	1.64087000	3.86665600
H	5.73336300	2.24978500	2.54729300
H	4.97139900	1.63353300	4.00143500
C	3.04390400	0.21734500	2.42877200
C	2.66869700	-0.20659000	3.86110400
H	2.17441500	-0.00870300	1.78821800
H	3.13421600	1.31582700	2.40276900
H	1.77596000	0.31611800	4.23715400
H	2.46210000	-1.28286700	3.91646800
H	3.48148200	-0.00518700	4.56914900

18-ts-iso

Zero-point correction= 1.075911 (Hartree/Particle)

Thermal correction to Energy=1.135180

Thermal correction to Enthalpy=1.136124

Thermal correction to Gibbs Free Energy=0.984026

E(Solv)= -2590.34275158

Pd	1.01452100	-0.08280700	-0.39278100
P	2.23454500	-1.93114300	0.52370000
P	2.33783400	1.91976700	0.14560900
C	3.95704300	-1.48212700	1.18667300
C	4.97047200	-0.77389300	0.30412300
C	6.00207200	-1.53463400	-0.27276300
C	7.03170700	-0.96547000	-1.01724000
C	7.07498600	0.41799400	-1.16661600
C	6.08760100	1.19343400	-0.56550900
C	5.01692700	0.63487300	0.15369000
C	4.06166500	1.57921200	0.85777500
C	2.55980000	-3.39519300	-0.70117700
C	2.96008600	-2.74564500	-2.04588700
C	3.67670900	-4.37243300	-0.27849200
C	1.28154300	-4.23353100	-0.91823400
C	1.41120000	-2.61793600	2.14174000
C	1.51559700	-1.53336200	3.23545000
C	2.04828100	-3.91234800	2.68397400
C	-0.09559300	-2.85262900	1.90046200
C	2.66779300	2.92308100	-1.47083400
C	3.01773700	1.87584100	-2.55409700
C	3.80273200	3.96380600	-1.41183600
C	1.37365600	3.64178800	-1.89962300
C	1.65308200	3.10395100	1.52345100
C	2.31816000	4.49282400	1.58224800
C	0.13538200	3.27936100	1.32036700
C	1.85166100	2.42690800	2.89788100

H	3.73858900	-0.85083500	2.05130400	C	-0.78208100	0.55194700	-3.14280900
H	4.41443800	-2.39413600	1.58425100	C	-0.93545200	0.10115900	-1.69925400
H	6.02134600	-2.60554600	-0.10318000	H	-1.70633000	0.37536000	-3.71059900
H	7.80513400	-1.59649900	-1.44588900	H	-0.55086500	1.61707100	-3.23555100
H	7.88248300	0.89434600	-1.71539600	H	0.01854000	-0.00691000	-3.63786000
H	6.16804900	2.27389600	-0.62675900	C	-0.51114600	-1.21210700	-1.31899900
H	4.56495800	2.54065400	1.00623300	H	-1.08897100	-1.73161400	-0.55827900
H	3.85322300	1.18980700	1.85751000	H	-0.14856300	-1.85908600	-2.11571200
H	2.17251300	-2.08891800	-2.42612900	C	-1.79609200	0.80511200	-0.80961800
H	3.87738900	-2.15558800	-1.96135400	C	-2.36606900	2.15689300	-1.12894100
H	3.13696200	-3.53461700	-2.78929300	H	-1.58040300	2.92042700	-1.08160100
H	4.65462800	-3.89574800	-0.22020000	H	-2.79122200	2.18161400	-2.13626900
H	3.47722500	-4.86214400	0.67633000	H	-1.76510400	0.52342400	0.23038700
H	3.75295600	-5.16107700	-1.03870200	H	-3.14505300	2.42386700	-0.41343700
H	1.45014200	-4.91206200	-1.76490600	C	-5.37972500	-0.77916800	-2.22761800
H	1.04506500	-4.85637200	-0.05094600	C	-5.88819900	-0.24295500	-1.00655100
H	0.40604800	-3.62782500	-1.15258700	C	-7.28505800	-0.09704700	-0.82955400
H	1.13655000	-0.57178800	2.87926400	C	-8.11450700	-0.48388300	-1.86488500
H	0.89498000	-1.83284000	4.08907400	C	-7.60864600	-1.02327800	-3.07781700
H	2.53440700	-1.39735100	3.61169200	C	-6.25046200	-1.17465200	-3.26739300
H	3.12989400	-3.82334600	2.83212000	C	-3.98355700	-0.74901500	-2.04921000
H	1.60587900	-4.14054900	3.66255400	H	-7.69375700	0.31722500	0.08192700
H	1.85720600	-4.77361700	2.03862900	H	-9.18951700	-0.36928800	-1.74987700
H	-0.29930100	-3.57647000	1.10929900	H	-8.30412500	-1.31450200	-3.86060800
H	-0.54459900	-3.24141200	2.82336000	H	-5.85567400	-1.58386500	-4.19428200
H	-0.61070900	-1.92021900	1.65425000	H	-3.19311900	-1.04402300	-2.72486900
H	3.16593800	2.38743600	-3.51490200	N	-3.69201300	-0.26293900	-0.83994500
H	3.93618700	1.33036400	-2.32078500	N	-4.83647500	0.05239900	-0.17465200
H	2.21243400	1.14460300	-2.67759200	B	-4.93114200	0.44601300	1.43966200
H	3.62072600	4.75522200	-0.68318200	C	-5.91306700	-0.66824000	2.15355000
H	4.77151400	3.51339300	-1.19246700	C	-5.50859900	-2.14304000	1.97971700
H	3.89004200	4.44207500	-2.39664800	H	-6.96185200	-0.56559900	1.83697400
H	1.49529100	4.01890600	-2.92365800	H	-5.93723500	-0.44360600	3.23113600
H	0.51443900	2.96659100	-1.89394600	H	-6.17546900	-2.83135100	2.51930500
H	1.14361800	4.50155900	-1.26354100	H	-5.52550500	-2.44066800	0.92318500
H	1.94608200	5.02629900	2.46701500	H	-4.48952100	-2.32825500	2.34554700
H	3.40825500	4.43734700	1.67442000	C	-3.40510600	0.36867100	2.07563200
H	2.07567300	5.10939600	0.71270900	C	-3.30184800	0.23636400	3.60818900
H	-0.38682600	2.31850200	1.33801700	H	-2.83029900	1.26755700	1.79111000
H	-0.27002200	3.89367000	2.13471200	H	-2.86213400	-0.48854400	1.64378500
H	-0.10669600	3.78318000	0.38252400	H	-2.25611300	0.23400100	3.95352800
H	1.48117600	1.39863000	2.90803800	H	-3.80856300	1.05666400	4.12833200
H	2.89630300	2.42706900	3.22402400	H	-3.75855800	-0.69462200	3.96305900
H	1.28020100	2.98478000	3.65007300	C	-5.56176100	1.97279500	1.46358500

C	-5.95728200	2.54805500	2.83589300
H	-6.45304000	2.02583900	0.81809700
H	-4.84777100	2.67702300	0.99829000
H	-6.42977400	3.53792200	2.75191900
H	-6.66865200	1.89386700	3.35583300
H	-5.08953400	2.66587400	3.49668800

19-ts

Zero-point correction= 0.866588 (Hartree/Particle)

Thermal correction to Energy= 0.915087

Thermal correction to Enthalpy= 0.916031

Thermal correction to Gibbs Free Energy= 0.785300

E(Solv)= -2327.64485253

Pd	-0.31993800	0.23566600	0.42359000
P	-2.09011000	1.86633600	0.27414400
P	-1.43595300	-1.94256800	0.41017300
C	-3.85382500	1.18066500	0.11641600
C	-4.23526200	0.21972600	-0.99235400
C	-5.00090000	0.71047900	-2.06437400
C	-5.48818500	-0.11061700	-3.07726400
C	-5.24771400	-1.48063800	-3.01225800
C	-4.53028500	-1.99125400	-1.93433800
C	-3.99638100	-1.17424500	-0.92208100
C	-3.32838100	-1.85616100	0.25748700
C	-1.88006000	3.04701700	-1.24188900
C	-1.44909800	2.14099600	-2.41842900
C	-3.14355300	3.82418600	-1.66306900
C	-0.75982600	4.07555800	-0.98175000
C	-2.33433000	2.91418800	1.89462900
C	-2.91848300	1.98952600	2.98759900
C	-3.27257700	4.12740200	1.73935800
C	-0.96769100	3.40374500	2.41511000
C	-0.91552500	-3.14538900	-1.01411900
C	-0.98999900	-2.29749100	-2.30700000
C	-1.78777400	-4.40711500	-1.16007400
C	0.54602000	-3.61190100	-0.86164700
C	-1.32740500	-2.81280200	2.14023000
C	-2.32848300	-3.96299300	2.36999800
C	0.09828400	-3.34331400	2.39369700
C	-1.60667700	-1.71122400	3.18700400
H	-4.01660600	0.68535300	1.07694800
H	-4.54273400	2.03159300	0.09261700
H	-5.25679100	1.76467900	-2.08457900
H	-6.07321400	0.31446400	-3.88791000
H	-5.63736300	-2.15121200	-3.77293700
H	-4.39833600	-3.06617500	-1.86249800
H	-3.71301000	-2.87756800	0.31657600
H	-3.63709500	-1.36522900	1.18694200
H	-0.51498100	1.61491000	-2.19872600
H	-2.20957800	1.39231800	-2.66041000
H	-1.29205700	2.75992900	-3.31210000
H	-3.94208300	3.16448600	-2.00152500
H	-3.53723100	4.46666300	-0.87306000
H	-2.88633800	4.47264000	-2.51112400
H	-0.53454700	4.59287900	-1.92358300
H	-1.05853300	4.84100600	-0.25921300
H	0.16412800	3.61231800	-0.63301700
H	-2.36044400	1.05214600	3.06994900
H	-2.84808600	2.50120500	3.95602800
H	-3.97578600	1.75753200	2.82732400
H	-4.25370500	3.85780900	1.33462400
H	-3.44408300	4.57195100	2.72887400
H	-2.84189200	4.90869300	1.10752300
H	-0.43796800	4.03675300	1.70025100
H	-1.12621300	3.99636300	3.32585700
H	-0.32023700	2.56142600	2.67159700
H	-0.66877900	-2.91499700	-3.15648600
H	-1.99734500	-1.93517100	-2.52162600
H	-0.32064600	-1.43311800	-2.24788400
H	-1.66246600	-5.09120700	-0.31567100
H	-2.85251000	-4.19323400	-1.26773100
H	-1.47568500	-4.95037300	-2.06188200
H	0.86714600	-4.06421800	-1.80898900
H	1.22245500	-2.78493800	-0.64776600
H	0.66680800	-4.37154500	-0.08683100
H	-2.16031800	-4.38410500	3.37019800
H	-3.37006100	-3.62914400	2.33598000
H	-2.20679100	-4.77829600	1.65215900
H	0.86720000	-2.62510200	2.09523900
H	0.22283300	-3.53744000	3.46698800
H	0.28645200	-4.28509700	1.87347900
H	-0.88315000	-0.89351500	3.10865700
H	-2.61323400	-1.28926000	3.09419600
H	-1.52886300	-2.14228700	4.19395900
C	2.24072600	0.16772800	2.37345800
C	1.90288400	0.36993500	0.90922900
H	2.11959700	-0.87446200	2.68577700
H	3.29671400	0.42623300	2.52013600

H	1.62632200	0.79728700	3.02577300
C	1.35918500	1.60156100	0.46807100
H	1.29901600	2.42236500	1.17489700
H	1.51311700	1.91038300	-0.56255200
C	2.33860200	-0.61306400	-0.01954500
C	2.67694200	-0.44194600	-1.40349500
H	2.23908700	0.41705900	-1.91580500
H	2.61231600	-1.56768000	0.42191200
C	7.31171000	0.01512700	0.25127800
C	6.60176000	0.20349800	-0.97761200
C	7.27826700	0.59313600	-2.15341900
C	8.64649500	0.78729800	-2.08075700
C	9.35882700	0.60327900	-0.86563800
C	8.70706900	0.22108300	0.29453000
C	6.28910800	-0.36532100	1.15788600
H	6.73577600	0.73482500	-3.08508500
H	9.19373200	1.08753500	-2.97176900
H	10.43391100	0.76710100	-0.85342200
H	9.26244100	0.08300800	1.22028500
H	6.36179800	-0.61513600	2.21000600
N	5.10944200	-0.39705600	0.52854600
N	5.28298400	-0.05152600	-0.75882800
H	3.89414000	-0.21147700	-1.32203300
H	2.61408500	-1.35196600	-2.00567500

20-ts

Zero-point correction= 0.953266 (Hartree/Particle)

Thermal correction to Energy= 1.006206

Thermal correction to Enthalpy= 1.007150

Thermal correction to Gibbs Free Energy=0.869162

E(Solv)= -2394.91262419

Pd	0.03935100	0.07104200	0.11283100
P	-1.34704000	1.65018400	1.08883800
P	-1.15653700	-2.08080200	-0.08396400
C	-3.07477800	0.96590800	1.49361600
C	-3.99275900	0.38014000	0.43801100
C	-5.05400200	1.19045100	-0.00655900
C	-6.01612700	0.74200100	-0.90602700
C	-5.95191800	-0.57172100	-1.36763900
C	-4.93152700	-1.39970600	-0.91054500
C	-3.93418700	-0.95684500	-0.02210100
C	-2.96158000	-1.99971400	0.49280400
C	-1.63632800	3.22661900	0.01290200
C	-2.26475200	2.76054200	-1.31970900
C	-2.55002500	4.28348900	0.66271700
C	-0.28569600	3.88543300	-0.34305200
C	-0.81670800	2.14246200	2.88816900
C	-0.37591300	0.83779400	3.58637500
C	-1.92650100	2.78926400	3.74531700
C	0.39878400	3.08989900	2.85335100
C	-1.20217700	-2.71710500	-1.89923600
C	-1.95697500	-1.65561500	-2.73097500
C	-1.87805000	-4.08456400	-2.10431200
C	0.24471500	-2.77051300	-2.44052600
C	-0.55735400	-3.48257400	1.10935200
C	-1.55617000	-4.63950500	1.32496300
C	0.78227600	-4.06671500	0.62065000
C	-0.30479800	-2.80568600	2.47432700
H	-2.85773100	0.20759400	2.25448500
H	-3.62553300	1.75968000	2.00603400
H	-5.13051200	2.20330300	0.38155600
H	-6.81672000	1.40455000	-1.22287100
H	-6.70062300	-0.95736600	-2.05395200
H	-4.91134400	-2.43742600	-1.23532000
H	-3.41032100	-2.98323400	0.32607500
H	-2.86438200	-1.89515300	1.57997000
H	-1.66542200	1.97297400	-1.78542700
H	-3.28250200	2.38752000	-1.20507700
H	-2.29434200	3.61200800	-2.01238900
H	-3.50524800	3.87013500	1.00307300
H	-2.06866400	4.77726500	1.51203200
H	-2.77932700	5.06331400	-0.07549700
H	-0.47658400	4.71438300	-1.03725800
H	0.23546400	4.29758500	0.52204000
H	0.38591200	3.18385800	-0.84555000
H	0.47281400	0.37749200	3.07686100
H	-0.07096100	1.07038200	4.61544000
H	-1.18333500	0.10008100	3.64767000
H	-2.74678300	2.09704700	3.95831900
H	-1.49501900	3.07724400	4.71291400
H	-2.34776200	3.69204500	3.29707500
H	0.12103800	4.10701100	2.56184300
H	0.83062900	3.14950700	3.86077900
H	1.18338400	2.73036200	2.18051800
H	-1.83616100	-1.88861600	-3.79716600
H	-3.02571400	-1.62879700	-2.51429300
H	-1.55529300	-0.65160100	-2.56121400
H	-1.29906500	-4.90162100	-1.66395400

H	-2.89184800	-4.12009300	-1.68960300
H	-1.96065900	-4.29060000	-3.18004100
H	0.21086900	-2.98007600	-3.51800200
H	0.76603100	-1.81816600	-2.30232700
H	0.84657500	-3.55266800	-1.97486000
H	-1.08823800	-5.38998000	1.97582700
H	-2.47317400	-4.31346800	1.82544500
H	-1.83468600	-5.14349300	0.39637400
H	1.50969500	-3.28796300	0.37149200
H	1.21813700	-4.67876200	1.42109800
H	0.65595200	-4.71779100	-0.24937900
H	0.46373800	-2.03282900	2.40612700
H	-1.21020200	-2.35198900	2.89332600
H	0.03955800	-3.56180100	3.19226500
C	3.39641600	1.39032500	-2.75957700
C	2.08405500	1.03615100	-2.34829200
C	0.98108200	1.24202200	-3.19986300
C	1.22389000	1.79281600	-4.44675200
C	2.53332300	2.14322400	-4.86572300
C	3.62172600	1.94638900	-4.03584200
C	4.20069000	1.04700800	-1.65228500
H	-0.01996900	0.96649000	-2.88698300
H	0.39173300	1.95792100	-5.12635600
H	2.67630700	2.56819100	-5.85527400
H	4.62573600	2.20982800	-4.35737300
H	5.26964200	1.11871800	-1.52680400
N	3.44623300	0.53637600	-0.67901000
N	2.13161400	0.49901000	-1.08511900
H	1.18926100	1.16451100	-0.14857400
B	4.10180700	-0.20924100	0.67293000
C	2.96907700	-0.14607700	1.85612100
C	3.14163600	-1.12118500	3.03545700
H	1.97095100	-0.35409200	1.41820600
H	2.89362700	0.87844000	2.25902300
H	2.35747700	-0.99707000	3.79732700
H	3.10059800	-2.16345600	2.69758700
H	4.10163700	-0.98731700	3.54392200
C	4.43024500	-1.73099200	0.11571100
C	5.47537900	-2.55617600	0.88977900
H	4.78748000	-1.65867500	-0.92476300
H	3.49877100	-2.31950400	0.05167500
H	5.62804800	-3.54772800	0.43952700
H	6.45077000	-2.05408600	0.89565700
H	5.19196600	-2.71771800	1.93597300
C	5.45004800	0.68925400	0.99979900
C	6.03974400	0.58451700	2.41857100
H	5.22787600	1.75593400	0.82644300
H	6.26158300	0.44637400	0.29157400
H	6.94277600	1.20161200	2.52896100
H	5.32414800	0.92687700	3.17604600
H	6.31890400	-0.44335200	2.67677400

21

Zero-point correction=0.309166 (Hartree/Particle)

Thermal correction to Energy=0.325774

Thermal correction to Enthalpy=0.326718

Thermal correction to Gibbs Free Energy=0.265204

E(Solv)= -642.184345728

C	-1.91581900	-0.80589700	-0.37789600
C	-1.85783200	0.48073800	0.26010100
C	-3.05174700	1.14331600	0.63470200
C	-4.25908000	0.52469200	0.37197000
C	-4.31815000	-0.75079200	-0.25981200
C	-3.16481500	-1.41640500	-0.63350000
C	-0.56204400	-1.11925400	-0.58082200
H	-3.00413900	2.11702700	1.11677600
H	-5.18939300	1.01697300	0.65106900
H	-5.29017700	-1.20322600	-0.44854100
H	-3.22123600	-2.39117500	-1.11615600
H	-0.09932300	-1.98760600	-1.02424800
N	0.18465300	-0.10184700	-0.10100200
N	-0.57957300	0.89003100	0.41947200
B	1.79824500	0.03647800	-0.15565200
C	2.16121900	1.00468000	-1.44720900
C	1.75647400	2.48580500	-1.35722200
H	1.70469000	0.57339100	-2.35585500
H	3.24973600	0.95790300	-1.62652200
H	0.68646800	2.58769500	-1.14290500
H	2.28989800	2.99388700	-0.54253500
H	1.97270800	3.04465100	-2.28304800
C	2.28988600	0.68757100	1.27154200
C	2.01136200	-0.15098500	2.53057000
H	1.81514400	1.66891000	1.40941300
H	3.37390900	0.88959800	1.21484400
H	2.36157500	0.33399300	3.45660000
H	0.93420200	-0.32705600	2.64566700
H	2.49588600	-1.13647500	2.48162800
C	2.40410900	-1.48294400	-0.41178200

C	3.93630000	-1.59066300	-0.31817200
H	2.10847300	-1.85453900	-1.40955700
H	1.97195800	-2.20389000	0.30311700
H	4.30595900	-2.60849900	-0.52285200
H	4.43078000	-0.91942400	-1.03300600
H	4.29691600	-1.31099700	0.68025800

21-iso

Zero-point correction= 0.309025 (Hartree/Particle)

Thermal correction to Energy= 0.325659

Thermal correction to Enthalpy= 0.326604

Thermal correction to Gibbs Free Energy= 0.264945

E(solv)=-642.18367227

C	-2.15433300	1.10063500	-0.07905000
C	-1.18593300	0.04633700	-0.05297500
C	-1.61834700	-1.29939600	-0.08856500
C	-2.97843500	-1.55630800	-0.15859400
C	-3.93648000	-0.51227500	-0.19199700
C	-3.53100700	0.81141300	-0.15157100
C	-1.35999800	2.27152300	-0.01411800
H	-0.90553000	-2.11323400	-0.05672500
H	-3.32074500	-2.58942500	-0.18606400
H	-4.99539200	-0.75831600	-0.24694900
H	-4.26318700	1.61753900	-0.17262800
H	-1.67406600	3.30973300	-0.00836700
N	-0.06949500	1.96682300	0.04123900
N	0.06267700	0.61215400	0.01125300
B	1.55715100	-0.02250500	0.15774000
C	1.86996800	-0.18669400	1.77443300
C	0.99676900	-1.16532900	2.57848700
H	1.79439500	0.80769000	2.24633000
H	2.92387700	-0.48917600	1.90503200
H	-0.06622900	-0.89886200	2.51315500
H	1.08987000	-2.19372100	2.20188400
H	1.25677100	-1.19366600	3.64975800
C	1.66957900	-1.46929800	-0.63238000
C	1.35107600	-1.46642300	-2.13801200
H	1.07834100	-2.26685300	-0.15412700
H	2.71135500	-1.80947200	-0.50600300
H	1.50610800	-2.44867300	-2.61431800
H	0.30939400	-1.17637500	-2.32695600
H	1.98112400	-0.74406400	-2.67342400
C	2.58871200	1.08886600	-0.50227300
C	4.06598500	0.66524900	-0.58697400
H	2.53087000	2.02432100	0.07210100
H	2.25337200	1.36648900	-1.51553500
H	4.71322900	1.46236300	-0.98844000
H	4.46761700	0.39604000	0.40004200
H	4.20546500	-0.21246200	-1.23337100

22

Zero-point correction= 0.602181 (Hartree/Particle)

Thermal correction to Energy= 0.638798

Thermal correction to Enthalpy= 0.639743

Thermal correction to Gibbs Free Energy= 0.528656

E(Solv) = -2029.64698778

C	-2.27520600	-2.16919200	2.16141600
O	-1.02040000	-2.84841000	1.90432400
C	-0.30109000	-2.05245200	1.09233700
C	-2.35656000	-1.15274100	1.00549700
H	-3.06839100	-2.91598200	2.16141400
H	-2.19778600	-1.69474000	3.14543800
H	-2.73573500	-0.19018700	1.35745700
N	-0.92474000	-1.01171900	0.62743400
C	1.05113600	-2.56830900	0.78537800
C	1.25187900	-3.93797000	1.04106500
C	2.10298800	-1.81195100	0.21014500
C	2.44889300	-4.56683300	0.71071300
H	0.44990500	-4.51229100	1.48723600
C	3.29848600	-2.46754900	-0.11698900
C	3.47445100	-3.83147600	0.12221000
H	2.57230600	-5.62689100	0.90921400
H	4.10912200	-1.90282300	-0.56414600
H	4.41218200	-4.30885500	-0.14627800
P	1.99325400	0.01350300	-0.02603800
Pd	-0.19157600	0.71193100	-0.44248400
C	-3.21915300	-1.64714000	-0.15342500
C	-2.68120100	-2.17654800	-1.33167400
C	-4.61326100	-1.62173900	0.00286700
C	-3.52119600	-2.66874700	-2.33583200
H	-1.60550200	-2.20464000	-1.47357400
C	-5.45022400	-2.12166900	-0.99331900
H	-5.04778600	-1.20152000	0.90718200
C	-4.90541700	-2.64562300	-2.16925800
H	-3.08845500	-3.07728500	-3.24485200
H	-6.52735900	-2.09897100	-0.85396600
H	-5.55662200	-3.03377300	-2.94699500
C	3.27149000	0.37314300	-1.29235900

C	4.47156800	1.03215000	-0.98925900
C	3.00854200	-0.00163800	-2.62247400
C	5.39546900	1.30697700	-2.00184100
H	4.68896100	1.33171700	0.03085100
C	3.93704600	0.26651800	-3.62628200
H	2.07717800	-0.50481500	-2.87256500
C	5.13211500	0.92427300	-3.31729300
H	6.32124300	1.81979100	-1.75767300
H	3.72724800	-0.03211300	-4.64932100
H	5.85230500	1.13912300	-4.10126500
C	2.61235200	0.70527700	1.56230100
C	3.57233500	0.04310500	2.34561900
C	2.11417200	1.94506400	1.99642100
C	4.02771500	0.61696300	3.53328900
H	3.96295200	-0.92114700	2.03638900
C	2.57490000	2.51726800	3.18260800
H	1.36044500	2.45751700	1.40570300
C	3.53192700	1.85362100	3.95265300
H	4.76854300	0.09514700	4.13207100
H	2.18261800	3.47678600	3.50715700
H	3.88737000	2.29590700	4.87872000
C	-2.14982500	1.86263800	-1.05837600
C	-1.02184100	2.69383800	-1.01406400
H	-2.31589000	1.27404800	-1.96087900
H	-0.97193200	3.47416000	-0.25545000
C	0.14946900	2.40853600	-1.76186100
C	1.33444300	3.34198200	-1.73761400
H	0.01677700	1.86468800	-2.70094200
H	1.19696200	4.12737800	-2.49355400
H	1.44958000	3.83314500	-0.76562500
C	-3.32911800	2.05902500	-0.19864500
C	-4.60185200	1.72789000	-0.69681700
C	-3.23948400	2.61498300	1.09358600
C	-5.74960200	1.97282200	0.05603300
H	-4.68934000	1.28949600	-1.68716400
C	-4.38605200	2.84978000	1.84845400
H	-2.26626700	2.85692500	1.51331400
C	-5.64649700	2.53475700	1.33009000
H	-6.72452200	1.72554000	-0.35393600
H	-4.29831900	3.28029600	2.84197300
H	-6.54003600	2.72498500	1.91743100
H	2.26553800	2.82312000	-1.97697200

23-ts

Zero-point correction= 0.786891 (Hartree/Particle)
Thermal correction to Energy= 0.832513
Thermal correction to Enthalpy= 0.833457
Thermal correction to Gibbs Free Energy= 0.699443
E(Solv) = -2434.12873165

C	-2.76224700	4.00705500	0.40287600
O	-3.77589900	3.11060500	-0.11007300
C	-3.19998300	1.89801800	-0.26492400
C	-1.44264500	3.24235400	0.14271900
H	-2.84497600	4.95231300	-0.13393500
H	-2.96289500	4.16049400	1.46805500
H	-0.80283000	3.26401000	1.02827500
N	-1.92022800	1.85396100	-0.07135700
C	-4.13771700	0.84357000	-0.72063000
C	-5.36178000	1.31228400	-1.23785100
C	-3.88195800	-0.55297800	-0.71638200
C	-6.31043200	0.44171000	-1.76450000
H	-5.55807100	2.37669500	-1.23462200
C	-4.85576800	-1.40861200	-1.25392700
C	-6.05562000	-0.92711400	-1.77754900
H	-7.23989100	0.83592000	-2.16376100
H	-4.67292900	-2.47772600	-1.26130300
H	-6.78382500	-1.62154000	-2.18638700
P	-2.35732300	-1.30382500	0.01934100
Pd	-0.53701100	0.18908200	0.10580600
C	-0.65912900	3.78211900	-1.04764800
C	-0.82108200	3.28019500	-2.34446300
C	0.20398100	4.86888200	-0.84503200
C	-0.13170000	3.85288700	-3.41678000
H	-1.48037500	2.43557900	-2.51922500
C	0.88768700	5.44502500	-1.91615500
H	0.34343300	5.26426700	0.15844700
C	0.72271700	4.93627300	-3.20693800
H	-0.27008100	3.45469200	-4.41840700
H	1.54874100	6.28972500	-1.74284300
H	1.25339700	5.38416100	-4.04228900
C	-2.19329000	-2.88414600	-0.91687600
C	-2.43277700	-4.14239300	-0.34546800
C	-1.76892500	-2.81407700	-2.25674400
C	-2.25713500	-5.30559100	-1.10114700
H	-2.76156300	-4.21838900	0.68606400
C	-1.60730600	-3.97543100	-3.01089200
H	-1.57158400	-1.84567400	-2.71161700
C	-1.84910500	-5.22560700	-2.43288500

H	-2.44744200	-6.27374900	-0.64648200
H	-1.29069200	-3.90601000	-4.04798800
H	-1.71916700	-6.13092900	-3.01883900
C	-2.88478300	-1.80597500	1.71494400
C	-4.22605200	-1.89534500	2.11776000
C	-1.87530400	-2.10911200	2.64592200
C	-4.54778500	-2.28255600	3.42066300
H	-5.02430800	-1.66163700	1.42125000
C	-2.19990100	-2.50802200	3.94223800
H	-0.83256600	-2.02861800	2.35023700
C	-3.53836100	-2.59204500	4.33337400
H	-5.59034200	-2.34275900	3.71993200
H	-1.40912200	-2.74404100	4.64896500
H	-3.79250900	-2.89349300	5.34548300
C	1.40694700	1.01928600	0.47674900
C	1.48970200	-0.41768100	0.54105300
H	1.73523300	1.48917000	-0.45293800
H	1.58318800	-0.90306900	1.51188900
C	1.79201900	-1.21008900	-0.60085500
C	1.65292200	-2.70708400	-0.58843600
H	1.68815000	-0.73156800	-1.57183500
H	2.30748800	-3.18023800	-1.32590800
H	1.86949100	-3.12298900	0.40066100
C	1.56394400	1.87825600	1.67138600
C	2.17712400	3.13944100	1.54691700
C	1.12582100	1.48288400	2.95218200
C	2.35319300	3.96919100	2.65478000
H	2.52154200	3.46706000	0.56924100
C	1.30332000	2.31142400	4.05828700
H	0.62543600	0.52636100	3.07813900
C	1.91847500	3.55895400	3.91663400
H	2.83773800	4.93424700	2.53261800
H	0.95647900	1.98444800	5.03484400
H	2.05645800	4.20266100	4.78045700
H	0.62522700	-2.97980300	-0.85011500
C	4.63952900	-1.66673600	0.28258000
C	6.14596700	-1.50721900	0.15113600
C	6.73015300	-1.17562000	-1.08174600
C	5.86947900	-0.89135300	-2.30052900
C	4.43317800	-1.40207200	-2.13207000
H	6.50101000	-1.98466900	2.22295900
H	4.28327800	-1.24283800	1.22749000
C	6.95939400	-1.73072900	1.26936300
C	8.12558500	-1.07626500	-1.16669300
H	5.84442700	0.19176900	-2.49282500
H	4.40291600	-2.49538200	-2.19901300
C	8.93065900	-1.30251800	-0.05310400
C	8.34494800	-1.63127100	1.17260100
H	8.58199300	-0.81913100	-2.11992800
H	10.01039600	-1.22345000	-0.13947600
H	8.96467600	-1.80536200	2.04729100
N	3.88301600	-1.03182500	-0.81278200
H	3.92957700	-0.01769200	-0.70030800
H	4.37010300	-2.73124200	0.28482800
H	3.77782900	-0.99969600	-2.91228900
H	6.31121500	-1.34510800	-3.19606200

24

Zero-point correction= 0.789987 (Hartree/Particle)
Thermal correction to Energy= 0.835338
Thermal correction to Enthalpy= 0.836282
Thermal correction to Gibbs Free Energy= 0.703446
E(Solv)= -2434.14645737

C	-2.48361900	4.11957100	0.64880700
O	-3.57070300	3.30690000	0.14772300
C	-3.06649200	2.07664700	-0.10668400
C	-1.22066700	3.29360300	0.29636400
H	-2.53355900	5.09132600	0.15653800
H	-2.62654900	4.23830100	1.72758700
H	-0.55290800	3.21995700	1.15873200
N	-1.78565700	1.95287200	0.01332900
C	-4.07557800	1.09420800	-0.57030200
C	-5.28627900	1.64890000	-1.02942600
C	-3.89554700	-0.31335600	-0.62423000
C	-6.29735400	0.85273500	-1.55776600
H	-5.42179200	2.72179300	-0.97889600
C	-4.93175200	-1.09190000	-1.16298600
C	-6.11819200	-0.52611000	-1.63030700
H	-7.21538600	1.31171000	-1.91192000
H	-4.80946600	-2.16826600	-1.21716500
H	-6.89521100	-1.16393000	-2.04179700
P	-2.38758900	-1.17085600	0.03995100
Pd	-0.48166700	0.19490300	0.06594700
C	-0.44090500	3.86123600	-0.88282700
C	-0.70343500	3.48290300	-2.20499300
C	0.52334900	4.84989200	-0.64042400
C	-0.01514800	4.08188900	-3.26318800
H	-1.44077700	2.71280500	-2.40973900

C	1.20685500	5.45304800	-1.69706100
H	0.74298100	5.14598800	0.38269300
C	0.93975800	5.06903400	-3.01349200
H	-0.23268700	3.78074100	-4.28454800
H	1.94696500	6.22185800	-1.49222900
H	1.46907400	5.53907600	-3.83763600
C	-2.36677200	-2.73438100	-0.94424500
C	-2.61571300	-3.99670000	-0.38688800
C	-2.03047000	-2.64919300	-2.30818300
C	-2.53406300	-5.14774500	-1.17711600
H	-2.87985000	-4.08567300	0.66206000
C	-1.96383500	-3.79697400	-3.09665300
H	-1.82855500	-1.67754600	-2.75413700
C	-2.21287000	-5.05171400	-2.53114500
H	-2.73136600	-6.11875100	-0.73124400
H	-1.71740200	-3.71364000	-4.15184000
H	-2.15805600	-5.94682400	-3.14420600
C	-2.90656500	-1.69702800	1.73400600
C	-4.24554500	-1.83312400	2.13292000
C	-1.89164200	-1.97718100	2.66531200
C	-4.55895200	-2.24414200	3.43014600
H	-5.04913400	-1.61467500	1.43740300
C	-2.20695900	-2.39847800	3.95739100
H	-0.85261500	-1.85471300	2.37201100
C	-3.54256900	-2.53029200	4.34334000
H	-5.59998300	-2.33966600	3.72585700
H	-1.41101300	-2.61312500	4.66531600
H	-3.79009300	-2.84895900	5.35192100
C	1.57311700	0.76326900	0.31445200
C	1.42076200	-0.67097800	0.27775600
H	1.90371700	1.25430600	-0.60663700
H	1.52403200	-1.21678500	1.21778900
C	1.93600000	-1.43302200	-0.91177100
C	1.54955600	-2.90540000	-0.96178700
H	1.64668100	-0.93196600	-1.84063400
H	2.04010600	-3.44763200	-1.77712300
H	1.76480600	-3.41583400	-0.01743600
C	1.89095200	1.53889700	1.53712600
C	2.56763900	2.76873200	1.42544900
C	1.55204400	1.09711500	2.83292800
C	2.89568500	3.52223000	2.55357200
H	2.83616500	3.13739600	0.43810900
C	1.88305500	1.84784200	3.95928600
H	1.00522400	0.16630200	2.95736400
C	2.55750400	3.06557500	3.82852000
H	3.42318200	4.46523400	2.43466500
H	1.60749500	1.48309000	4.94545600
H	2.81336100	3.64904100	4.70812500
H	0.47182700	-2.96556100	-1.12768200
C	4.25909400	-1.95176500	0.15432400
C	5.74912100	-1.68705300	0.08446900
C	6.37031100	-1.29465100	-1.11083700
C	5.55270700	-1.03334900	-2.36272500
C	4.14661500	-1.62367700	-2.28273700
H	6.02895800	-2.17614600	2.16659600
H	3.83251100	-1.58783900	1.09152800
C	6.51643700	-1.87852200	1.24110100
C	7.75808200	-1.10280800	-1.12441200
H	5.48165800	0.04885400	-2.54703700
H	4.16260500	-2.71438700	-2.34474300
C	8.51873500	-1.30184700	0.02474800
C	7.89554900	-1.69130900	1.21373000
H	8.24317500	-0.79527700	-2.04770400
H	9.59382100	-1.15253700	-0.00501200
H	8.48090600	-1.84255900	2.11547200
N	3.51821600	-1.27120100	-0.96172400
H	3.63862000	-0.26126100	-0.81513200
H	4.03479400	-3.01948100	0.06972400
H	3.49874000	-1.23822200	-3.07402900
H	6.05122400	-1.45389100	-3.24363000

25

Zero-point correction= 0.587750 (Hartree/Particle)
Thermal correction to Energy= 0.624616
Thermal correction to Enthalpy= 0.625560
Thermal correction to Gibbs Free Energy= 0.511320
E(Solv)= -2029.15848187

C	-4.75685800	0.35909700	-0.74710000
O	-4.04112900	-0.61857900	-1.53617300
C	-2.72164600	-0.48406000	-1.23310800
C	-3.66480400	0.99991500	0.15946700
H	-5.21402800	1.08027600	-1.43068900
H	-5.53840100	-0.16304600	-0.19048500
H	-3.79706800	0.69017800	1.20257000
N	-2.41428600	0.38145500	-0.33285200
C	-1.81665700	-1.33773700	-2.03603300
C	-2.24525600	-1.64311200	-3.33778600
C	-0.57313000	-1.83587300	-1.56514800

C	-1.45031500	-2.39582700	-4.19907900
H	-3.20820900	-1.27311300	-3.67144000
C	0.20339100	-2.59657800	-2.44959500
C	-0.21844600	-2.86791600	-3.75341300
H	-1.79406200	-2.60853800	-5.20722500
H	1.15623300	-2.98912100	-2.11254400
H	0.41588800	-3.45600300	-4.41130200
P	0.02062100	-1.46555200	0.16211000
Pd	-0.36901000	0.80360000	0.61248900
C	-3.66092200	2.51553300	0.10495300
C	-3.18134200	3.19683100	-1.02153200
C	-4.19847900	3.25328200	1.16475300
C	-3.23879800	4.58874900	-1.08351000
H	-2.74161500	2.63573500	-1.84177400
C	-4.26182100	4.64733900	1.10304100
H	-4.56608800	2.73463200	2.04775600
C	-3.78172100	5.31836700	-0.02206300
H	-2.85498800	5.10544500	-1.95923200
H	-4.67986800	5.20630300	1.93595100
H	-3.82457700	6.40303000	-0.07090100
C	1.68622900	-2.26400800	0.22246800
C	1.89791400	-3.62629500	0.49211200
C	2.79783300	-1.43179900	0.01355500
C	3.19357900	-4.14363200	0.53816400
H	1.05251700	-4.28332000	0.67347600
C	4.09290800	-1.95278100	0.05211400
H	2.64820800	-0.36956500	-0.15497400
C	4.29217700	-3.30949900	0.31453000
H	3.34452600	-5.19883100	0.75185100
H	4.94028900	-1.29094300	-0.10448500
H	5.29983900	-3.71518100	0.35476500
C	-1.03196800	-2.60564700	1.17669700
C	-1.66479100	-3.75054600	0.66733900
C	-1.19987200	-2.29107300	2.53591800
C	-2.44037400	-4.56331000	1.49800800
H	-1.55563100	-4.01021700	-0.38169200
C	-1.96721800	-3.10838500	3.36687400
H	-0.73166600	-1.39452000	2.93446500
C	-2.59098900	-4.24600100	2.84914500
H	-2.92556600	-5.44521900	1.08747300
H	-2.08450300	-2.85160500	4.41653400
H	-3.19459900	-4.87920100	3.49411000
C	3.48712900	1.98449100	1.10922200
C	2.23021600	2.33884200	0.75220300
H	3.62258500	1.51732700	2.08570100
H	2.05774100	2.81268500	-0.21468500
C	1.03990800	2.14798800	1.57500300
C	-0.18713500	2.80681600	1.30441700
H	1.22560000	1.78120700	2.58542200
C	4.71258000	2.13503900	0.32241600
C	5.93377700	1.68325500	0.86468700
C	4.75055600	2.70820000	-0.96691000
C	7.13132100	1.79537200	0.15974400
H	5.93396800	1.24098100	1.85873800
C	5.94622400	2.82109500	-1.67011400
H	3.83358200	3.07090700	-1.42255600
C	7.14650500	2.36574900	-1.11442800
H	8.05500500	1.43815900	0.60880100
H	5.94289700	3.26855100	-2.66122800
H	8.07774900	2.45614900	-1.66707900
H	-0.88742900	2.99558700	2.11613900
H	-0.25238100	3.51611300	0.47969100

26-ts

Zero-point correction= 0.952594 (Hartree/Particle)

Thermal correction to Energy= 1.008496

Thermal correction to Enthalpy= 1.009440

Thermal correction to Gibbs Free Energy= 0.847905

E(Solv)= -2821.34636531

C	4.50694600	3.51732300	1.52988000
O	5.27821800	2.79211100	0.54099200
C	4.47317700	1.84453300	0.01104200
C	3.06365900	2.96783200	1.36598400
H	4.93502600	3.29966600	2.51206300
H	4.59991900	4.58354400	1.31526300
H	2.42423100	3.70471000	0.86683800
N	3.24825100	1.84292400	0.41870200
C	5.12585200	0.97854900	-0.99790000
C	6.30510600	1.48692600	-1.56993900
C	4.64376600	-0.29393900	-1.40864100
C	6.99974100	0.77890800	-2.54737500
H	6.67021000	2.45328100	-1.24428300
C	5.36698900	-0.98957400	-2.38694700
C	6.52804500	-0.46415900	-2.95861300
H	7.90202600	1.20005000	-2.98012100
H	5.01748900	-1.96326000	-2.71181900
H	7.05788900	-1.03155800	-3.71835400
P	3.11072000	-1.05525600	-0.68934800

Pd	1.54788900	0.66723000	-0.30220300
C	2.41097600	2.56982300	2.67560600
C	2.74734100	1.37403800	3.32440600
C	1.49572700	3.43807200	3.28336400
C	2.17376400	1.05331200	4.55532700
H	3.44890600	0.68558200	2.86215000
C	0.92637800	3.12124300	4.51883000
H	1.22952700	4.36919800	2.78811800
C	1.26335500	1.92620100	5.15696100
H	2.44224000	0.12123800	5.04505700
H	0.22252800	3.80783600	4.98117900
H	0.82287700	1.67816400	6.11862100
C	3.71919200	-1.91037000	0.82649600
C	5.06414400	-2.25673000	1.03061100
C	2.77636800	-2.22899800	1.81882700
C	5.45514500	-2.91122800	2.20111400
H	5.80950200	-2.01742800	0.27832700
C	3.16793600	-2.89224700	2.98214500
H	1.73530700	-1.95016800	1.67825000
C	4.50912400	-3.23218400	3.17644000
H	6.49966300	-3.17134700	2.34824100
H	2.42759900	-3.13714000	3.73888700
H	4.81606400	-3.74226600	4.08516100
C	2.70090300	-2.41661500	-1.86048000
C	1.96396700	-2.08474100	-3.01059700
C	3.06258200	-3.75520700	-1.64725600
C	1.61771400	-3.06636200	-3.93857500
H	1.66596300	-1.05194800	-3.17632900
C	2.70130900	-4.73906800	-2.57140300
H	3.62744400	-4.03252500	-0.76277400
C	1.98407900	-4.39720100	-3.71890300
H	1.05866100	-2.79454600	-4.82993300
H	2.98714300	-5.77231600	-2.39479000
H	1.70985500	-5.16370800	-4.43832400
C	-0.19758300	1.92985700	-0.23252300
C	-0.53021600	0.71411400	-0.91447600
H	-0.46477800	1.98197800	0.82463300
H	-0.64859300	0.71119000	-1.99727100
C	-0.84131300	-0.48077600	-0.21185900
C	-1.31643800	-1.68847300	-0.76126400
H	-0.82605500	-0.40742400	0.87778400
C	-0.10912600	3.24764600	-0.89426100
C	-0.37488800	4.41312000	-0.15008800
C	0.24107700	3.40006700	-2.25094900
C	-0.29688500	5.67780100	-0.73354600
H	-0.65761900	4.31812300	0.89612400
C	0.31453900	4.66273600	-2.83469300
H	0.47578300	2.52263800	-2.84769100
C	0.04728200	5.80933700	-2.08022400
H	-0.51263900	6.56055800	-0.13739600
H	0.58699100	4.75408600	-3.88280200
H	0.10693000	6.79249000	-2.53800200
H	-1.18519500	-2.57973900	-0.14529700
H	-1.11999700	-1.86299300	-1.82176100
C	-6.48350200	1.03688500	-0.61098500
C	-5.96854100	-0.01664400	-1.26894200
H	-5.78697000	1.69709400	-0.09088100
H	-6.61917900	-0.69109300	-1.82354800
C	-4.49144400	-0.29179600	-1.36550900
C	-4.01715400	-0.32982700	-2.82528200
H	-3.94406500	0.50325100	-0.84636300
H	-2.96345700	-0.61371400	-2.90317200
C	-7.89202800	1.43798900	-0.50388900
C	-8.20914500	2.57413000	0.26169500
C	-8.94363600	0.74111200	-1.12912400
C	-9.52847800	3.00125200	0.40350500
H	-7.40890400	3.12659100	0.74896200
C	-10.26057400	1.16667000	-0.98810200
H	-8.73320000	-0.13675400	-1.73329800
C	-10.55917600	2.29794000	-0.22095900
H	-9.75045000	3.88195100	0.99927100
H	-11.05896500	0.61729800	-1.47890300
H	-11.58861200	2.62750100	-0.11436700
H	-4.13521800	0.66985100	-3.25301900
C	-4.30725200	-1.42578800	0.83884500
C	-3.99186500	-2.68843100	1.61463300
C	-3.88028300	-3.93398800	0.98156500
C	-3.99899300	-4.03662300	-0.52418200
C	-4.67880700	-2.81550700	-1.14327300
H	-3.94117200	-1.63326400	3.49498700
H	-3.67807000	-0.59829200	1.18762100
C	-3.85096000	-2.60143300	3.00671700
C	-3.62870800	-5.07392100	1.75752400
H	-3.00363600	-4.16434600	-0.97051800
H	-5.74915600	-2.80607300	-0.90550100
C	-3.49202000	-4.98377100	3.14001300
C	-3.60408200	-3.73993500	3.76873500
H	-3.54336200	-6.04085300	1.26692000

H	-3.30282200	-5.87814600	3.72644700
H	-3.50028700	-3.65932700	4.84681700
N	-4.06880500	-1.55711600	-0.62481500
H	-5.35074000	-1.12316100	0.99013100
H	-4.57007400	-2.82969600	-2.22899000
H	-4.57418500	-4.92758400	-0.80233700
H	-2.81453500	-1.60465200	-0.72300500
H	-4.61190300	-1.01511500	-3.43759900

27-ts

Zero-point correction= 0.951465 (Hartree/Particle)

Thermal correction to Energy= 1.007413

Thermal correction to Enthalpy= 1.008357

Thermal correction to Gibbs Free Energy= 0.851572

E(Solv)= -2821.34226348

C	4.28065800	1.95014600	-2.64550600
O	4.04839100	0.58727600	-3.06655000
C	3.09063200	0.07437800	-2.25562800
C	3.30055700	2.15160600	-1.45110000
H	5.33033500	2.04388300	-2.35763100
H	4.07316100	2.60432800	-3.49548400
H	2.54750300	2.90776000	-1.70319500
N	2.61391700	0.84213400	-1.34069000
C	2.74829600	-1.33395900	-2.55914500
C	3.76941200	-2.10086700	-3.14415000
C	1.46635700	-1.90921100	-2.35083300
C	3.55912800	-3.42706700	-3.51232400
H	4.73503800	-1.63943300	-3.31418000
C	1.27118500	-3.23428600	-2.76556600
C	2.30033900	-3.99123800	-3.33048900
H	4.36749900	-4.00232100	-3.95320400
H	0.29400100	-3.68866700	-2.66297000
H	2.10527900	-5.01514700	-3.63639900
P	0.07888100	-0.94573700	-1.56194000
Pd	0.83697800	0.24817000	0.37966900
C	4.00562900	2.57588000	-0.17502100
C	4.74829400	1.65727600	0.58040300
C	3.97059700	3.91554400	0.23006200
C	5.43476700	2.07254900	1.72211500
H	4.77550200	0.61167500	0.28580600
C	4.66172300	4.33400500	1.36960900
H	3.40205500	4.63949500	-0.35037000
C	5.39391000	3.41213300	2.11955200
H	5.99920500	1.34781800	2.30206700
H	4.62578900	5.37725000	1.67023300
H	5.93118100	3.73506400	3.00668700
C	-1.38295700	-2.08168000	-1.65003400
C	-2.53036000	-1.73674200	-2.38101200
C	-1.38964100	-3.29219500	-0.93309300
C	-3.64770800	-2.57542700	-2.39154000
H	-2.56011700	-0.81338900	-2.94773500
C	-2.49993100	-4.13492200	-0.95678600
H	-0.51074500	-3.59799800	-0.37589500
C	-3.63739100	-3.77572700	-1.68346200
H	-4.52728000	-2.28483600	-2.95813200
H	-2.47578900	-5.07228200	-0.40759100
H	-4.50751800	-4.42494400	-1.69416600
C	-0.31735800	0.31591000	-2.85401600
C	-0.03732500	0.10348600	-4.21310900
C	-0.96971500	1.49725900	-2.46971800
C	-0.39324900	1.06110700	-5.16474300
H	0.45444300	-0.80920300	-4.53483100
C	-1.33661700	2.44686900	-3.42550800
H	-1.19401300	1.67270500	-1.42185000
C	-1.04384800	2.23366100	-4.77405200
H	-0.16783800	0.88576000	-6.21293400
H	-1.85285300	3.35089600	-3.11392500
H	-1.32450500	2.97424100	-5.51763800
C	-3.65337000	-1.81465400	2.36991400
C	-2.52278200	-1.08986400	2.35531600
H	-3.76109700	-2.57571700	3.14530000
H	-2.34715300	-0.33862300	1.58689500
C	-1.48464000	-1.16745100	3.44513000
C	-1.53524000	0.10651700	4.30666900
H	-1.72235200	-2.01916900	4.10179900
H	-0.91312500	0.03954100	5.20332500
H	-1.23825200	0.99356300	3.74016000
C	-4.80154200	-1.70047100	1.45900000
C	-5.75067600	-2.73755100	1.42933100
C	-5.01169200	-0.58136400	0.63206900
C	-6.85929400	-2.67442700	0.58622700
H	-5.61350200	-3.60189300	2.07521000
C	-6.12005200	-0.51613900	-0.20817900
H	-4.32025800	0.25585500	0.66520900
C	-7.04669000	-1.56331000	-0.23813300
H	-7.58097300	-3.48655500	0.58082100
H	-6.27084300	0.36167200	-0.83063400
H	-7.91594200	-1.50481600	-0.88716000

H	-2.56818000	0.25163000	4.63437900
C	0.96439500	-1.24143800	3.88032700
C	2.33142800	-1.71783800	3.43748800
C	2.47941100	-2.67180300	2.42381400
C	1.26161500	-3.16370100	1.67514900
C	-0.04827900	-2.85693200	2.39801800
H	3.34573600	-0.49329800	4.89546600
H	1.01231400	-0.18213500	4.13027600
C	3.46412000	-1.23159900	4.10501600
C	3.76289500	-3.12629400	2.09272400
H	1.24755300	-2.69962000	0.68091000
H	-0.15918600	-3.51268200	3.27554700
C	4.88619400	-2.64489000	2.76126000
C	4.73617300	-1.69185600	3.77362300
H	3.87676600	-3.87087000	1.30786900
H	5.87353600	-3.01544900	2.50090800
H	5.60527100	-1.31495000	4.30511700
N	-0.10903900	-1.44756300	2.87292000
H	0.11617100	-0.58492600	1.67966600
H	0.66765200	-1.77315900	4.79999500
H	-0.90753200	-3.02298700	1.74719400
H	1.32216300	-4.24746600	1.51364000
C	-1.54481200	3.68421900	0.45207000
C	-0.90367200	2.79085700	1.24387200
H	-0.96412900	4.15550800	-0.34213500
H	-1.42813600	2.31709100	2.06965000
C	0.49803800	2.45954000	1.07539800
C	1.28257800	1.76015400	1.98042000
H	1.00003800	2.96544800	0.25134500
C	-2.93845200	4.11643700	0.53336700
C	-3.37854300	5.14322300	-0.32460100
C	-3.87350900	3.55103700	1.42506800
C	-4.69685900	5.59318300	-0.29376300
H	-2.67041100	5.59667500	-1.01467700
C	-5.19018300	3.99882100	1.45406900
H	-3.57044800	2.75512600	2.09932500
C	-5.60840200	5.02214900	0.59599600
H	-5.01172100	6.38977600	-0.96174100
H	-5.89552900	3.55024500	2.14794200
H	-6.63692100	5.37002500	0.62329400
H	2.36471300	1.82546600	1.93678800
H	0.85671800	1.46582300	2.93335900

28

Zero-point correction= 0.598740 (Hartree/Particle)
Thermal correction to Energy=0.635753
Thermal correction to Enthalpy= 0.636697
Thermal correction to Gibbs Free Energy= 0.522630
E(Solv) =-2029.61102079

C	0.98111300	-3.35239600	2.25932600
O	2.24622900	-2.76112800	1.87278500
C	1.99259900	-1.85402900	0.91351000
C	-0.01687300	-2.83519800	1.18667000
H	1.10086600	-4.43628500	2.25897600
H	0.74897600	-3.00256400	3.26883500
H	-0.87570100	-2.35788100	1.66855800
N	0.77289400	-1.78704600	0.48407900
C	3.18469900	-1.10430100	0.45745400
C	4.41465600	-1.77439700	0.55255700
C	3.14748600	0.21925900	-0.04759800
C	5.59501300	-1.17010800	0.12366500
H	4.43769200	-2.77930400	0.95780600
C	4.34625000	0.81232600	-0.46211600
C	5.56038100	0.12472300	-0.38680300
H	6.53334300	-1.71161700	0.19189800
H	4.33556700	1.82537300	-0.84829400
H	6.47284900	0.60837600	-0.72240700
P	1.57227600	1.17577000	-0.13614900
Pd	-0.06473900	-0.24876100	-0.87502800
C	-0.51459800	-3.93119800	0.25814600
C	0.28980700	-4.43864700	-0.77194800
C	-1.77299800	-4.50463400	0.48262800
C	-0.16265400	-5.49358900	-1.56635000
H	1.26874400	-4.00614600	-0.96105300
C	-2.22453500	-5.56262400	-0.30916500
H	-2.40321600	-4.12696600	1.28573100
C	-1.42052600	-6.05739600	-1.33737400
H	0.46860900	-5.87676200	-2.36304600
H	-3.20252600	-5.99687700	-0.12322100
H	-1.77034900	-6.87851700	-1.95596700
C	1.95688700	2.66703000	-1.12648300
C	2.36193800	3.86603400	-0.51941100
C	1.86616400	2.59676800	-2.52638100
C	2.67192600	4.97751500	-1.30591900
H	2.42865900	3.93865700	0.56148400
C	2.18708600	3.70682100	-3.30632400
H	1.53700400	1.67782600	-3.00361500
C	2.58770600	4.89901900	-2.69701100

H	2.97597100	5.90449600	-0.82863900
H	2.11387000	3.64421600	-4.38810700
H	2.82729800	5.76613700	-3.30554400
C	1.25103100	1.74994700	1.57578200
C	2.27064800	1.84488200	2.53618800
C	-0.05903800	2.12706100	1.91820300
C	1.98138200	2.31410400	3.81925700
H	3.28762300	1.55673700	2.28803100
C	-0.33940500	2.60138200	3.19934100
H	-0.85583700	2.04848500	1.18312900
C	0.67934800	2.69340800	4.15134100
H	2.77535900	2.38431500	4.55708300
H	-1.35355300	2.89473300	3.45465100
H	0.45840300	3.05852800	5.15011200
H	-0.52119900	0.92608900	-1.76463300
C	-4.12660500	0.03296500	-0.13156600
C	-3.23717300	-0.09344900	-1.14710300
H	-4.08935000	-0.71559700	0.66141000
H	-3.22560700	0.61576700	-1.97159800
C	-2.28925700	-1.18547600	-1.20827400
C	-1.44155100	-1.45915500	-2.26277000
H	-2.34549300	-1.91434500	-0.40133900
C	-5.15778600	1.05114700	0.03716700
C	-5.99831000	0.97770700	1.16541800
C	-5.35692000	2.10737500	-0.87626600
C	-7.00087400	1.92094500	1.37731300
H	-5.86071300	0.16732700	1.87760500
C	-6.35751400	3.04883000	-0.66357000
H	-4.72858100	2.19129700	-1.75796200
C	-7.18351500	2.96011700	0.46294500
H	-7.63988900	1.84448000	2.25217900
H	-6.49871500	3.85488700	-1.37780600
H	-7.96529600	3.69680600	0.62338400
H	-0.92447700	-2.41222200	-2.31077100
H	-1.49247200	-0.88127100	-3.18102900

29-ts

Zero-point correction= 0.597488 (Hartree/Particle)

Thermal correction to Energy= 0.633958

Thermal correction to Enthalpy= 0.634903

Thermal correction to Gibbs Free Energy= 0.522858

E(Solv)= -2029.60512671

C	1.65035900	1.76842600	2.51088500
O	0.26295600	2.18088200	2.59993700
C	-0.36949600	1.66954500	1.52941700
C	1.79818200	1.35536200	1.02997700
H	2.27298200	2.61290800	2.80481700
H	1.78993000	0.93383000	3.20539300
H	2.36147100	0.42212200	0.94095400
N	0.38761400	1.10956300	0.63550800
C	-1.82955500	1.91426100	1.51817100
C	-2.26948900	3.02339000	2.26189900
C	-2.77672600	1.13629300	0.80659200
C	-3.61368600	3.38782400	2.28276600
H	-1.54454100	3.60802500	2.81507800
C	-4.12434600	1.51631700	0.84760000
C	-4.54392800	2.63497000	1.57087900
H	-3.92682100	4.25660900	2.85331700
H	-4.85952600	0.92946300	0.30804300
H	-5.59503200	2.90766000	1.57831100
P	-2.28630800	-0.38178400	-0.11447700
Pd	-0.19725200	-0.08184300	-1.08341700
C	2.46981200	2.43048500	0.18079500
C	1.75182300	3.31711100	-0.62957600
C	3.86442800	2.56731600	0.26519500
C	2.41673700	4.31514400	-1.34916400
H	0.67272100	3.22843100	-0.70760100
C	4.52655600	3.56724800	-0.44682800
H	4.43599400	1.88922300	0.89634200
C	3.80281800	4.44325500	-1.26059700
H	1.84594100	4.99583000	-1.97459800
H	5.60579800	3.66214900	-0.36772000
H	4.31694900	5.22068600	-1.81788600
C	-3.70003600	-0.75131200	-1.22157900
C	-4.70700400	-1.66174700	-0.86662100
C	-3.77093700	-0.09320900	-2.46107400
C	-5.76826400	-1.90729600	-1.74110000
H	-4.66320500	-2.18328800	0.08429500
C	-4.83783700	-0.33407900	-3.32532000
H	-2.98989600	0.60592400	-2.74989800
C	-5.83654600	-1.24408700	-2.96727200
H	-6.53977000	-2.61895800	-1.46192800
H	-4.88545600	0.18076000	-4.28049600
H	-6.66237300	-1.43870000	-3.64526900
C	-2.25694800	-1.71319500	1.14968400
C	-2.92208600	-1.59926500	2.38069300
C	-1.55081000	-2.89290100	0.85671700
C	-2.88171000	-2.65014100	3.29952300

H	-3.47179700	-0.69607100	2.62628700
C	-1.52056800	-3.94215300	1.77452100
H	-1.02658700	-2.98864300	-0.09055400
C	-2.18376900	-3.82101900	2.99831200
H	-3.39815900	-2.55209500	4.24999200
H	-0.97540400	-4.85105500	1.53688800
H	-2.15482000	-4.63619800	3.71540400
H	-0.65877900	-0.89443200	-2.36005500
C	3.86445200	-0.85841100	-1.01601700
C	2.66032900	-1.18127200	-1.54883000
H	4.18239800	0.18088800	-1.09377800
H	2.31864900	-2.21555300	-1.54288200
C	1.79356700	-0.21949600	-2.20902100
C	0.77981400	-0.62170000	-3.10469800
H	2.12441600	0.81651500	-2.24222100
C	4.82391300	-1.75169700	-0.37044500
C	6.08924900	-1.23974300	-0.02152700
C	4.54883700	-3.10289000	-0.07538600
C	7.04750700	-2.04422600	0.59112900
H	6.32121100	-0.20151000	-0.24774100
C	5.50517400	-3.90510500	0.53749000
H	3.58031000	-3.52799500	-0.32291500
C	6.75866600	-3.38064100	0.87284000
H	8.01872000	-1.63006700	0.84598800
H	5.27595900	-4.94411900	0.75651300
H	7.50273400	-4.01137600	1.35040100
H	0.40634300	0.09064400	-3.83505300
H	0.74002400	-1.66225600	-3.42130900

30

Zero-point correction= 0.072584 (Hartree/Particle)
Thermal correction to Energy= 0.076262
Thermal correction to Enthalpy= 0.077206
Thermal correction to Gibbs Free Energy= 0.046350
E(Solv)= -226.290238920

C	-1.09736900	-0.36702400	-0.00013100
C	-0.68448900	0.98352300	0.00003000
C	0.69087500	0.91869300	0.00001500
N	1.00481500	-0.39775600	0.00005000
H	1.92334000	-0.81383600	0.00012000
H	-2.10307100	-0.76357000	-0.00022400
H	-1.29867600	1.87032100	0.00004700
H	1.45258400	1.68385000	0.00003800
N	-0.06599800	-1.20051800	0.00002700

31

Zero-point correction= 0.242891 (Hartree/Particle)
Thermal correction to Energy= 0.256024
Thermal correction to Enthalpy= 0.256968
Thermal correction to Gibbs Free Energy=0.200742
E(Solv)= -613.524318877

C	3.83431000	-1.43233200	-0.65043800
C	2.53695300	-0.93298800	-0.72021700
C	2.18055000	0.24829100	-0.04241400
C	3.17666900	0.90935300	0.69781300
C	4.47685700	0.41051800	0.76860700
C	4.81145600	-0.76455200	0.09479700
H	4.08690300	-2.34554900	-1.18282600
H	1.79658000	-1.46278200	-1.31239600
H	2.92233600	1.82581100	1.22572400
H	5.22730500	0.94053600	1.34906200
H	5.82350900	-1.15663200	0.14533900
C	0.82527000	0.82209200	-0.07044900
H	0.73489700	1.78077100	0.43774500
C	-0.26790400	0.29413900	-0.64132600
H	-0.21283200	-0.66980000	-1.14513800
C	-1.63978700	0.92471500	-0.67751500
C	-1.77589600	2.27864900	0.02868900
H	-1.52099400	2.20183500	1.09107000
H	-1.12292800	3.02528100	-0.43525200
H	-1.93086900	1.05437400	-1.72741900
C	-4.54819600	-0.94213600	0.00902000
C	-3.77490600	-1.45220800	1.07440400
C	-2.54644100	-0.83379300	0.93241700
N	-2.63485000	-0.02953100	-0.16261700
H	-5.57336600	-1.17180100	-0.25243600
H	-4.06240900	-2.17120700	1.82797300
H	-1.63191100	-0.90672500	1.50290500
N	-3.85938400	-0.07709300	-0.73617600
H	-2.81020300	2.62377000	-0.05311900

32

Zero-point correction= 0.080563 (Hartree/Particle)
Thermal correction to Energy= 0.086904
Thermal correction to Enthalpy= 0.087849
Thermal correction to Gibbs Free Energy= 0.047779
E(Solv)= -687.146831365

C	-1.25418700	1.15572400	-0.00018800

C	-2.48350500	0.46421100	0.00072000
C	-2.13479700	-0.87455500	0.00045000
N	-0.78036100	-0.91872500	-0.00081700
H	-1.07099800	2.22146300	-0.00039700
H	-3.47992800	0.88003200	0.00132600
H	-2.73118800	-1.77538700	0.00067200
N	-0.22479600	0.30943100	-0.00095900
H	-0.17432100	-1.72710300	0.00013700
H	1.52704600	0.28832300	-0.00115500
Cl	2.83531900	-0.00556200	0.00035100

33-ts

Zero-point correction= 1.026275 (Hartree/Particle)

Thermal correction to Energy= 1.085781

Thermal correction to Enthalpy= 1.086726

Thermal correction to Gibbs Free Energy=0.926251

E(Solv)= -2906.62424475

Pd	-1.35775700	0.03411200	0.23353200
P	-2.94980200	1.56597200	1.17118100
P	-2.65650100	-1.92484600	-0.44679700
C	-4.75401000	0.97579400	1.14327600
C	-5.45726100	0.57009700	-0.13940900
C	-6.37194900	1.47185500	-0.71018700
C	-7.17207800	1.14273300	-1.80097100
C	-7.10806100	-0.14663000	-2.32183000
C	-6.23408700	-1.06553100	-1.74756800
C	-5.37456000	-0.73541800	-0.68534600
C	-4.52055700	-1.83867700	-0.08967400
C	-2.99147900	3.29794800	0.31315700
C	-2.95029100	3.00903300	-1.20534100
C	-4.23235200	4.16217900	0.61562500
C	-1.74472300	4.12680500	0.68498000
C	-2.73681900	1.77816000	3.09112600
C	-3.10670500	0.43683600	3.76579700
C	-3.60108300	2.89050400	3.71691200
C	-1.25637200	2.04835000	3.42933100
C	-2.53468800	-2.25627600	-2.34418400
C	-2.65611300	-0.87124500	-3.02091000
C	-3.60648900	-3.19166800	-2.93693900
C	-1.15071900	-2.83868800	-2.68745000
C	-2.21734000	-3.53231400	0.53844300
C	-2.82658100	-4.83320500	-0.01752900
C	-0.68281800	-3.67274800	0.59727500
C	-2.71090200	-3.34598000	1.99141900
H	-4.74326200	0.11472100	1.81574100
H	-5.35971700	1.74048800	1.64063700
H	-6.49283900	2.45106600	-0.25936500
H	-7.86224200	1.87532500	-2.20972900
H	-7.74973300	-0.44756800	-3.14510600
H	-6.24340600	-2.08734900	-2.11223700
H	-4.96087300	-2.80391500	-0.36127600
H	-4.56800600	-1.77807100	1.00142600
H	-2.04955000	2.45328100	-1.48326100
H	-3.81886200	2.43391900	-1.53969000
H	-2.94739200	3.96060000	-1.75377300
H	-5.16036100	3.69391200	0.28835200
H	-4.33004600	4.41436700	1.67311700
H	-4.14094100	5.10685400	0.06336200
H	-1.71380100	5.01755300	0.04379400
H	-1.77429600	4.47855600	1.72061800
H	-0.81371200	3.57992700	0.53040100
H	-2.60410400	-0.40980500	3.28708500
H	-2.77796700	0.46601000	4.81236800
H	-4.18426000	0.24691100	3.77558900
H	-4.66412600	2.79422300	3.47255200
H	-3.51525200	2.83361400	4.81025300
H	-3.26781500	3.88917700	3.42169800
H	-0.86994400	2.95953900	2.96786800
H	-1.15655200	2.16361500	4.51664100
H	-0.62214000	1.21308600	3.12251000
H	-2.56850300	-0.99282200	-4.10901900
H	-3.61731700	-0.39175500	-2.81468300
H	-1.86072100	-0.19533900	-2.68939600
H	-3.61857600	-4.17879900	-2.47041500
H	-4.60795500	-2.76744900	-2.87509000
H	-3.38833700	-3.33907700	-4.00314200
H	-1.00881900	-2.79507200	-3.77548100
H	-0.33799000	-2.27865500	-2.22272100
H	-1.05869500	-3.88868500	-2.39351900
H	-2.61261400	-5.65429700	0.67979600
H	-3.91532200	-4.78017000	-0.12708100
H	-2.39617400	-5.11281800	-0.98284300
H	-0.22407000	-2.78820000	1.04629000
H	-0.42527500	-4.54206900	1.21698400
H	-0.22948600	-3.82182500	-0.38399700
H	-2.37550500	-2.39346300	2.41505600
H	-3.80004200	-3.40581700	2.08176200
H	-2.29194800	-4.14725800	2.61331600

C	1.64994600	-0.55380800	1.52996900
C	1.00123100	0.03115200	0.29494400
H	1.53767300	-1.64174100	1.58629100
H	2.72562000	-0.32632800	1.49400600
H	1.22621000	-0.11959200	2.44139100
C	0.35553400	1.29888400	0.35891500
H	0.48152100	1.88428800	1.26364500
H	0.30502100	1.89613700	-0.55286900
C	1.21946700	-0.55095000	-0.97548300
C	2.07768100	-1.64685700	-1.26851200
H	2.27195400	-2.35486700	-0.46108100
H	1.91791800	-2.13401700	-2.23272200
H	0.84467200	0.01900100	-1.82686100
P	5.30037300	-0.13606100	-0.55362100
O	4.60186600	0.34102300	0.68532000
O	4.45392800	-0.59577100	-1.75555400
H	3.22322200	-1.09013600	-1.45564700
O	6.37213800	0.97750700	-1.10407900
O	6.33581600	-1.36031600	-0.26456000
C	5.89800200	2.31327600	-1.29928100
H	5.44413900	2.68126900	-0.36991600
H	5.12353300	2.31641000	-2.07998900
C	7.07555700	3.18602100	-1.71557000
H	7.84458600	3.13474400	-0.93292700
H	7.52418600	2.76204600	-2.62382100
C	6.67418300	4.64604800	-1.96072400
H	6.21609700	5.05713600	-1.04968900
H	5.89650000	4.68564600	-2.73698700
C	7.85491100	5.52892000	-2.37927600
H	7.54082100	6.56566700	-2.54849600
H	8.63474200	5.53778900	-1.60778200
H	8.31281200	5.16281900	-3.30645200
C	7.21738200	-1.25892600	0.86120700
H	7.94537800	-0.45624800	0.68055700
H	6.63929200	-0.99685800	1.75611900
C	7.92545400	-2.59635800	1.03717700
H	8.45321600	-2.84150200	0.10581800
H	7.16854700	-3.37892500	1.18292900
C	8.91038100	-2.59350700	2.21319100
H	9.65683800	-1.80055400	2.06267300
H	8.37494800	-2.33508400	3.13799200
C	9.62585200	-3.93617300	2.39772300
H	10.19841800	-4.20536400	1.50155000
H	10.32325500	-3.90632400	3.24318600
H	8.90794900	-4.74434400	2.58508600

34-ts

Zero-point correction= 1.026397 (Hartree/Particle)

Thermal correction to Energy= 1.085948

Thermal correction to Enthalpy= 1.086892

Thermal correction to Gibbs Free Energy=0.927931

E(Solv)= -2906.61889013

Pd	-1.27489100	0.52403300	0.68596300
P	-1.43029200	1.38341100	-1.53667800
P	-3.39571700	-0.60678000	1.14336400
C	-3.02151400	0.89924600	-2.45731900
C	-3.45365700	-0.54831700	-2.61365500
C	-3.24316300	-1.17409000	-3.85451400
C	-3.71577400	-2.45085800	-4.14466700
C	-4.47157300	-3.12586000	-3.19030700
C	-4.72422200	-2.50835400	-1.96870700
C	-4.21124200	-1.24252500	-1.63662400
C	-4.61976000	-0.63506100	-0.30746100
C	0.00663000	0.83151900	-2.70841600
C	0.26188600	-0.66188100	-2.39951400
C	-0.29493200	0.97587600	-4.21493600
C	1.30225100	1.61646600	-2.41402300
C	-1.61917300	3.31756700	-1.60400200
C	-2.99341600	3.69907200	-1.01044800
C	-1.52371600	3.92477400	-3.01781000
C	-0.55332300	3.97473700	-0.70264300
C	-3.18580200	-2.46292300	1.63791900
C	-2.14098700	-3.04080800	0.65480900
C	-4.45829400	-3.33037500	1.57156700
C	-2.61354500	-2.56782600	3.06531400
C	-4.48620200	0.29490300	2.47027700
C	-5.69687700	-0.50459100	2.98796400
C	-3.58718800	0.68601500	3.66086600
C	-5.01129500	1.61045400	1.85388500
H	-3.80083700	1.43458300	-1.90960000
H	-2.98071200	1.35604800	-3.45170600
H	-2.72511700	-0.62729700	-4.63454400
H	-3.52266600	-2.89346600	-5.11779800
H	-4.88563100	-4.10821100	-3.39953600
H	-5.37025500	-3.01259500	-1.25757200
H	-5.54376000	-1.11710700	0.02973900
H	-4.86327300	0.42029900	-0.45776300
H	0.64637700	-0.80269600	-1.38701200

H	-0.63825600	-1.27153900	-2.52905800
H	1.02696000	-1.04008300	-3.08977600
H	-1.10052700	0.32019400	-4.54465000
H	-0.53859000	1.99749800	-4.51321700
H	0.60277000	0.67991200	-4.77299700
H	2.12286300	1.14455200	-2.96787000
H	1.23892600	2.65784500	-2.74565000
H	1.58773900	1.58838500	-1.36315800
H	-3.16343100	3.21261700	-0.04544300
H	-3.01784400	4.78360200	-0.84270600
H	-3.82831500	3.46091300	-1.67654900
H	-2.23168800	3.47389500	-3.72125900
H	-1.76341700	4.99525200	-2.96115800
H	-0.51871400	3.84237300	-3.43925300
H	0.46834200	3.72666600	-0.99607400
H	-0.66096800	5.06610200	-0.76332600
H	-0.68903100	3.68126700	0.34173500
H	-1.95643100	-4.09408400	0.90707200
H	-2.48415400	-3.00323400	-0.38272700
H	-1.18956600	-2.50670800	0.72158400
H	-5.24663500	-2.98962800	2.24514600
H	-4.87034200	-3.39055500	0.56430200
H	-4.19655000	-4.35465200	1.86917300
H	-2.29189100	-3.60254400	3.24225600
H	-1.74322100	-1.92347100	3.20585100
H	-3.35761600	-2.32547500	3.83036600
H	-6.30524300	0.14506300	3.63186500
H	-6.34725600	-0.85749800	2.18029300
H	-5.39951500	-1.36583500	3.59192300
H	-2.76758500	1.33208000	3.33627100
H	-4.18490100	1.23768500	4.39921900
H	-3.15356800	-0.17862700	4.16817200
H	-4.20683700	2.19206200	1.39397600
H	-5.79562200	1.44667800	1.10843200
H	-5.44857200	2.22616000	2.65056500
C	0.00705200	1.72167800	3.45241700
C	0.32816400	0.71983800	2.35367100
H	-0.61229400	1.28368800	4.24233700
H	0.93591700	2.07402200	3.92217200
H	-0.51428200	2.60184300	3.06136500
C	0.72770900	1.18413100	1.06860300
H	0.87875100	2.25436400	0.95495800
H	1.41080700	0.57472000	0.47961300
C	0.48261400	-0.64503400	2.71610300
C	1.14660700	-1.68640100	2.02286600
H	0.17633400	-0.87506400	3.73950700
P	4.21922500	-0.63119400	0.83903200
O	3.24198900	-0.49542800	-0.29480200
O	3.73058400	-1.28906900	2.14879800
H	2.43729100	-1.46706600	2.20283200
O	5.58671200	-1.39669200	0.37001000
O	4.86719700	0.78640200	1.30102600
C	5.45378700	-2.69239700	-0.22833100
H	4.81831800	-2.61949000	-1.12061800
H	4.96567600	-3.37316600	0.48301700
C	6.84412500	-3.20095400	-0.58657700
H	7.31798600	-2.48023500	-1.26675100
H	7.45623800	-3.22150100	0.32476600
C	6.81729200	-4.59195200	-1.23281200
H	6.19235100	-4.56342500	-2.13688000
H	6.33158800	-5.30285300	-0.54927500
C	8.21368300	-5.10879900	-1.59552700
H	8.16597700	-6.10326600	-2.05434900
H	8.71216900	-4.43693500	-2.30514100
H	8.85161900	-5.18132900	-0.70613200
C	5.43783100	1.64522900	0.30363800
H	6.34463000	1.17615500	-0.10021300
H	4.72498500	1.77328800	-0.52098500
C	5.76617800	2.98276800	0.95435400
H	6.43968500	2.80518300	1.80317300
H	4.84329500	3.40858700	1.37125200
C	6.40642700	3.97478000	-0.02514500
H	7.32367400	3.53553800	-0.44239000
H	5.73100400	4.13498600	-0.87791600
C	6.73781700	5.32390300	0.62191500
H	7.43901300	5.20022400	1.45627600
H	7.19423800	6.01255500	-0.09864600
H	5.83513000	5.80494600	1.01858200
H	1.18273900	-1.63170300	0.93450800
H	0.96017300	-2.69297500	2.40068800

35-ts

Zero-point correction= 1.025876 (Hartree/Particle)

Thermal correction to Energy= 1.085704

Thermal correction to Enthalpy= 1.086648

Thermal correction to Gibbs Free Energy=0.926168

E(Solv)= -2906.60991603

Pd	-1.15091100	0.17145100	0.53162300

P	-2.19064800	2.04091200	-0.71769300
P	-2.92519100	-1.19809400	1.30922000
C	-4.07310800	1.98532500	-0.91135800
C	-4.72954400	0.75043200	-1.49883800
C	-5.24317700	0.83819900	-2.80435100
C	-5.92942300	-0.20890100	-3.41249000
C	-6.15289400	-1.37903700	-2.69167000
C	-5.69037700	-1.46742700	-1.38162200
C	-4.96368400	-0.43685500	-0.76058500
C	-4.62249400	-0.57968100	0.71323100
C	-1.49684700	2.08406700	-2.52373200
C	-1.49306900	0.61733400	-3.01482300
C	-2.31526300	2.93049700	-3.51965300
C	-0.04091000	2.59809300	-2.54465900
C	-1.97673900	3.77899700	0.11027000
C	-2.93045400	3.86080500	1.32313200
C	-2.27159600	4.98119900	-0.80715900
C	-0.53670500	3.89140100	0.65557800
C	-2.88849400	-3.06507800	0.82538300
C	-2.52791200	-3.09956900	-0.67814800
C	-4.19806500	-3.84889600	1.04620500
C	-1.76804100	-3.78002600	1.60465000
C	-3.18072700	-1.01009800	3.22439700
C	-4.22051900	-1.96382900	3.84259300
C	-1.82609900	-1.20939700	3.93160900
C	-3.62122100	0.44241200	3.51410600
H	-4.44252200	2.13764300	0.10679000
H	-4.39079000	2.86243300	-1.48544300
H	-5.12713500	1.76982800	-3.34922800
H	-6.30611000	-0.09763700	-4.42530200
H	-6.70692700	-2.20491500	-3.12884200
H	-5.92778700	-2.35850200	-0.81080200
H	-5.38428900	-1.21086700	1.18368800
H	-4.70866200	0.40081700	1.18621700
H	-0.87503500	-0.01956500	-2.37404600
H	-2.49810500	0.18792000	-3.05026400
H	-1.07483000	0.58071000	-4.02948500
H	-3.33661600	2.56580800	-3.63491000
H	-2.35529800	3.98757300	-3.25060800
H	-1.83955100	2.86410500	-4.50697000
H	0.35430600	2.47639000	-3.56123700
H	0.03231200	3.66110300	-2.29822000
H	0.62957300	2.04346800	-1.88279700
H	-2.84721300	2.98336400	1.97198700
H	-2.66275700	4.73894700	1.92450700
H	-3.97803600	3.98032700	1.02946400
H	-3.26607300	4.92782600	-1.26352600
H	-2.23853600	5.90248700	-0.21011300
H	-1.53153100	5.08907600	-1.60412400
H	0.22637400	3.80050000	-0.12073400
H	-0.40795800	4.87301400	1.13050300
H	-0.33944100	3.12564200	1.41157800
H	-2.45639100	-4.14648400	-1.00276200
H	-3.28181600	-2.60939100	-1.29972500
H	-1.56179800	-2.62426100	-0.86452700
H	-4.45851400	-3.95077900	2.10110100
H	-5.05354100	-3.41208100	0.52923900
H	-4.06645300	-4.86272500	0.64553100
H	-1.62230300	-4.78057400	1.17661600
H	-0.81759100	-3.24949600	1.52843000
H	-2.01850500	-3.91401800	2.66143000
H	-4.39157300	-1.67337100	4.88787700
H	-5.18993600	-1.92613600	3.33454600
H	-3.87539500	-3.00104100	3.85152500
H	-1.09255100	-0.47874000	3.58270100
H	-1.96161900	-1.06877600	5.01238900
H	-1.40548500	-2.20498000	3.77931300
H	-2.96630700	1.16595800	3.01857400
H	-4.65603900	0.64292500	3.22006800
H	-3.55459800	0.62263400	4.59464000
C	1.32223300	-1.32921500	2.31207700
C	0.89099600	-0.80892600	0.96243000
H	0.89000800	-2.30262100	2.55930800
H	2.41842000	-1.42039700	2.27289700
H	1.07142900	-0.62476700	3.10943100
C	1.15022700	0.60251300	0.70743900
H	1.19861000	1.23082900	1.59844100
H	0.51916900	1.07426200	-0.10085400
C	1.03354500	-1.70479700	-0.19679600
C	1.39827800	-2.99796400	-0.16391500
H	1.62155600	-3.52029600	0.76088300
H	1.52299200	-3.56307100	-1.08275300
H	0.89241800	-1.23858200	-1.17105500
P	4.51546900	-0.01156800	-0.13311900
O	4.28657100	-0.66528800	1.19908700
O	3.41195200	0.88861200	-0.70740100
H	2.17595600	0.72594400	0.07883800
O	5.93508400	0.82020000	-0.14578000

O	4.85605800	-1.08347500	-1.31694600
C	6.17313400	1.73431600	0.92697000
H	6.06617200	1.21190900	1.88649500
H	5.42554600	2.54111800	0.89625600
C	7.57643300	2.30743400	0.76984100
H	8.29614800	1.47756500	0.77123800
H	7.65320600	2.78638500	-0.21552500
C	7.93445900	3.31288400	1.87148800
H	7.84323700	2.82697800	2.85343500
H	7.20257100	4.13347700	1.86920000
C	9.34570300	3.89079400	1.71916200
H	9.57520000	4.60600200	2.51790400
H	10.10313700	3.09794200	1.75300800
H	9.45783400	4.41269000	0.76073100
C	5.80118700	-2.12288200	-1.04072400
H	6.80846700	-1.68917800	-0.97210000
H	5.56534600	-2.58584800	-0.07435900
C	5.73343000	-3.14661800	-2.16742300
H	5.92530500	-2.63643300	-3.12096400
H	4.70875800	-3.53916100	-2.22349700
C	6.72747700	-4.29979100	-1.98044800
H	7.74770600	-3.89536200	-1.91671700
H	6.53652100	-4.79446800	-1.01733900
C	6.66184800	-5.33677900	-3.10698500
H	6.88275100	-4.87891600	-4.07913500
H	7.38226600	-6.14795600	-2.94856800
H	5.66290200	-5.78571600	-3.17380000

36-ts

Zero-point correction= 1.025311 (Hartree/Particle)

Thermal correction to Energy= 1.084917

Thermal correction to Enthalpy= 1.085862

Thermal correction to Gibbs Free Energy= 0.926916

E(Solv) = -2906.58650282

P	-2.13873700	-0.69985300	0.63639200
P	-2.40055000	1.25794100	-0.70421600
P	2.60633000	2.27194000	-1.49344100
C	-0.88978100	2.42027500	-0.63599200
C	-0.23757800	2.76985200	0.69351100
C	-0.89531600	3.62948900	1.58865300
C	-0.36443300	3.97907500	2.82811500
C	0.87468500	3.46570900	3.20449900
C	1.57182800	2.66310400	2.30543700
C	1.06495400	2.32379800	1.03648100
C	1.97203200	1.50991300	0.13428300
C	-3.93362100	2.39158300	-0.36078400
C	-4.16775000	2.41106500	1.16771300
C	-3.77091800	3.84026300	-0.86843700
C	-5.21093200	1.79638400	-0.99122300
C	-2.39001600	0.77586100	-2.58164300
C	-0.98921200	0.22681700	-2.93575100
C	-2.69968200	1.93556100	-3.54857500
C	-3.39002200	-0.37978500	-2.80453200
C	3.67645600	0.77166000	-2.09836000
C	2.66896600	-0.19528200	-2.76236200
C	4.44749600	-0.00649100	-1.01319900
C	4.67005600	1.22321000	-3.18638800
C	3.75065700	3.73461600	-0.96902700
C	4.91365200	3.39068100	-0.02382400
C	4.30051100	4.40334600	-2.24960100
C	2.83874100	4.78354400	-0.29055100
H	-0.15276200	1.93018500	-1.26811000
H	-1.16762400	3.33982600	-1.16310000
H	-1.85069300	4.05221200	1.30266600
H	-0.91552700	4.64441300	3.48732900
H	1.30698200	3.70625800	4.17215700
H	2.56030600	2.30050200	2.57893800
H	1.45210400	0.59856500	-0.18011500
H	2.82793500	1.18309500	0.73237900
H	-4.52143100	1.43961000	1.51384100
H	-3.27485300	2.66041900	1.74293600
H	-4.94138700	3.15533900	1.40167800
H	-2.97064100	4.38395000	-0.35971300
H	-3.57994400	3.89351900	-1.94293600
H	-4.70309100	4.38729900	-0.67437300
H	-6.07575000	2.36616300	-0.62472600
H	-5.21950100	1.86657600	-2.08166800
H	-5.36319700	0.75387200	-0.69907800
H	-0.62674400	-0.49720100	-2.20171300
H	-1.05217300	-0.27640300	-3.90985000
H	-0.24323500	1.02029700	-3.03742500
H	-2.02073400	2.78331900	-3.40605800
H	-2.56072100	1.58230100	-4.57928700
H	-3.72755200	2.29888000	-3.46923600
H	-4.42345500	-0.11252300	-2.57785800
H	-3.35022200	-0.68594700	-3.85854800
H	-3.12070100	-1.24648300	-2.19245900
H	3.20680000	-1.07241200	-3.14860100

H	1.91312700	-0.56281900	-2.06221800
H	2.15410300	0.27936500	-3.60470700
H	5.13126800	0.62968000	-0.44310900
H	3.77673000	-0.51161400	-0.31204800
H	5.05259200	-0.78904200	-1.49268100
H	5.07255400	0.33781000	-3.69735900
H	4.19403800	1.85380200	-3.94600600
H	5.52230700	1.76852000	-2.76939900
H	5.42986500	4.31056300	0.28690000
H	4.56854800	2.88869300	0.88697300
H	5.65839900	2.74860500	-0.50488700
H	3.49403900	4.63924200	-2.95350900
H	4.79381900	5.34768400	-1.98086600
H	5.03639800	3.79000800	-2.77249100
H	1.96339800	5.01645200	-0.90816000
H	2.48473700	4.46403000	0.69063900
H	3.40477900	5.71548300	-0.15354800
C	-4.96348300	-2.80672500	0.10000600
C	-5.04505400	-1.62978100	1.04596500
H	-4.17038600	-2.66546400	-0.64277500
H	-4.73190100	-3.73551500	0.63734400
H	-5.91150300	-2.95691000	-0.42551300
C	-6.21894900	-1.01120800	1.27047300
H	-7.13151300	-1.31676500	0.76497600
H	-6.30735700	-0.18727400	1.97411600
C	-3.81851100	-1.19805900	1.76247900
C	-2.79908200	-2.16907500	2.17401000
H	-4.00768100	-0.39395400	2.47415000
P	0.55417900	-2.37996300	0.49999100
O	0.01758500	-1.07475700	-0.06203900
O	-0.40761200	-3.10205800	1.46849700
H	-1.51972600	-2.42664000	1.58809000
O	0.96423800	-3.32548500	-0.73426800
O	1.99065900	-2.20036300	1.23424300
C	1.42428400	-4.67520600	-0.49674800
H	0.68519500	-5.19959400	0.11988200
H	2.37137700	-4.63308000	0.05433000
C	1.60453700	-5.35766900	-1.84457000
H	0.64544700	-5.34378900	-2.37884700
H	2.30976000	-4.76966400	-2.44639900
C	2.10790300	-6.80081100	-1.70536700
H	1.40204700	-7.37692300	-1.09057400
H	3.06203400	-6.80442500	-1.15960900
C	2.29219600	-7.49777400	-3.05762600
H	2.65209300	-8.52494100	-2.93014400
H	1.34714900	-7.54100100	-3.61252600
H	3.01869300	-6.96389500	-3.68222500
C	2.03420400	-1.67304500	2.57967800
H	1.67268600	-0.63717700	2.57039800
H	1.36812300	-2.26728600	3.21536600
C	3.47207300	-1.74349800	3.07358200
H	4.11355400	-1.17262200	2.38917500
H	3.81127400	-2.78677600	3.02610100
C	3.62334800	-1.20673100	4.50355200
H	3.26786500	-0.16729900	4.54326200
H	2.97098000	-1.77771400	5.17901300
C	5.06711900	-1.27209000	5.01292700
H	5.73878600	-0.68244300	4.37710100
H	5.14676600	-0.88298500	6.03422200
H	5.43831400	-2.30412800	5.01864600
H	-2.41556100	-2.01685800	3.18529600
H	-3.05401300	-3.21662900	1.99519900

37-ts

Zero-point correction= 1.024445 (Hartree/Particle)

Thermal correction to Energy= 1.084477

Thermal correction to Enthalpy= 1.085422

Thermal correction to Gibbs Free Energy= 0.923955

E(Solv) = -2906.58726636

Pd	1.47773900	1.58210800	0.18616400
P	3.40886200	0.25177800	0.03891500
P	0.23778300	-3.31330000	-0.90015500
C	3.01065500	-1.52196800	0.59242600
C	2.01222600	-1.64788000	1.72783600
C	2.49079100	-1.75448700	3.04381300
C	1.63590200	-1.80187300	4.14323300
C	0.25954300	-1.72454900	3.93535800
C	-0.23219900	-1.66792200	2.63312400
C	0.61206500	-1.67556400	1.50884100
C	-0.03141900	-1.72889900	0.13707600
C	4.77109500	0.87066500	1.25885400
C	4.04186000	1.37864100	2.52479100
C	5.79347200	-0.20701900	1.67221500
C	5.53563400	2.06611100	0.65383100
C	4.11836100	-0.03695000	-1.72777400
C	2.96082300	-0.61766100	-2.56959100
C	5.30966800	-1.01272200	-1.76678600
C	4.52153600	1.29858600	-2.38761900

C	-1.01399200	-3.00776800	-2.34056800
C	-0.74925300	-1.58760700	-2.89007400
C	-2.50836900	-3.11150600	-1.98876400
C	-0.67266500	-4.00412500	-3.47170500
C	-0.38463100	-4.77650000	0.19460000
C	-1.65079700	-4.53214400	1.03588300
C	-0.60563900	-6.01166200	-0.70349300
C	0.78835600	-5.11049400	1.14686600
H	2.62729600	-2.03310600	-0.29337400
H	3.94695700	-2.02137800	0.85857700
H	3.56374200	-1.80543000	3.20953400
H	2.04291600	-1.88677700	5.14712800
H	-0.42841300	-1.72495600	4.77639100
H	-1.30479800	-1.61609800	2.47122200
H	0.37772700	-0.93665900	-0.50130600
H	-1.09481700	-1.49615900	0.23544400
H	3.39107700	2.22788700	2.29475500
H	3.42688400	0.60948300	2.99714800
H	4.79121000	1.70981400	3.25676600
H	5.33725900	-1.02067000	2.24206100
H	6.31896800	-0.64376300	0.81894000
H	6.54969400	0.25334500	2.32152400
H	6.10150900	2.56255400	1.45297000
H	6.25730400	1.75501100	-0.10628500
H	4.86420000	2.80910100	0.21149100
H	2.08817900	0.04379600	-2.55343400
H	3.29354000	-0.70581900	-3.61189100
H	2.64387300	-1.61364800	-2.24771100
H	5.08055800	-1.97497100	-1.29700100
H	5.57344400	-1.21628800	-2.81305900
H	6.20025500	-0.59613100	-1.28653000
H	5.43476600	1.72201500	-1.96684900
H	4.71068300	1.11872200	-3.45404800
H	3.73092400	2.05031300	-2.31096000
H	-1.30126200	-1.46391900	-3.83195400
H	-1.09190200	-0.80501900	-2.20792500
H	0.31087800	-1.42791700	-3.11444200
H	-2.79971800	-4.12475700	-1.69702200
H	-2.79068000	-2.41941700	-1.18878100
H	-3.10833000	-2.84730200	-2.87162300
H	-1.29077700	-3.78222900	-4.35256500
H	0.37827800	-3.92096400	-3.77080500
H	-0.86059300	-5.04435800	-3.19490100
H	-1.89390000	-5.44335500	1.60151100

H	-1.50440500	-3.73161300	1.76394900
H	-2.52183600	-4.28423400	0.42535600
H	0.25146100	-6.20061100	-1.36032800
H	-0.73747800	-6.89914800	-0.06952600
H	-1.50170600	-5.92226300	-1.32450100
H	1.70317600	-5.33945200	0.58862700
H	1.00810300	-4.29496600	1.84049000
H	0.52966500	-5.99477700	1.74604900
C	0.86036100	3.61290500	-2.57934600
C	1.87114800	4.04593700	-1.54289200
H	0.91001900	2.52594600	-2.72748100
H	-0.17140800	3.82695800	-2.27981500
H	1.05555600	4.09843500	-3.54043900
C	2.94512400	4.79188600	-1.86675700
H	3.11844600	5.13013400	-2.88475300
H	3.67365600	5.10070100	-1.12065700
C	1.67185600	3.65913600	-0.12990300
C	0.34596300	3.51616500	0.45877000
H	2.46844100	4.00023600	0.53187600
P	-2.54810000	1.85823100	-0.27808500
O	-2.38961600	3.02892300	-1.19192400
O	-1.32961900	1.36457900	0.54209000
H	-0.28145500	2.33514600	0.47566300
O	-3.75597500	2.17402200	0.77779300
O	-3.05632800	0.50805200	-1.06134500
C	-4.00746200	1.27675200	1.86933500
H	-3.09916600	1.18023800	2.47638700
H	-4.26073100	0.28202400	1.47671800
C	-5.15932300	1.83694700	2.69383000
H	-4.89137300	2.84745200	3.02964900
H	-6.03960800	1.94599000	2.04627500
C	-5.50380000	0.95583500	3.90171500
H	-4.61497000	0.84649000	4.53948800
H	-5.75738800	-0.05667000	3.55611100
C	-6.66260800	1.51322500	4.73536500
H	-6.88771500	0.86582300	5.59076300
H	-6.42555900	2.51101300	5.12434400
H	-7.57555100	1.60181200	4.13384800
C	-3.96631900	0.64986300	-2.17185200
H	-3.74988100	1.58725000	-2.69505700
H	-3.73671400	-0.18314700	-2.84544800
C	-5.42813100	0.59180200	-1.73424400
H	-5.62775900	1.42243900	-1.04633500
H	-5.59249200	-0.33684800	-1.16981100

C -6.39423200 0.65480600 -2.92513700
H -6.21397500 1.57954700 -3.49100500
H -6.17920000 -0.17314100 -3.61636300
C -7.86640900 0.59568400 -2.50346000
H -8.12018000 1.43107600 -1.83967200
H -8.53356100 0.64445500 -3.37185500
H -8.08701900 -0.33408500 -1.96447000
H 0.28220000 3.80886300 1.50837100
H -0.48356400 3.90186000 -0.14283100

38

Zero-point correction= 0.835230 (Hartree/Particle)
Thermal correction to Energy= 0.882485
Thermal correction to Enthalpy= 0.883430
Thermal correction to Gibbs Free Energy= 0.754457
E(Solv)=-2014.87744868

Pd -2.77280400 -0.44123900 -0.76551600
P -1.04554300 0.94961900 0.20187700
P 4.13260900 0.13455800 -0.55181000
C 0.64787700 0.16439000 -0.12222200
C 1.00086900 -1.15001400 0.55677900
C 0.07213600 -2.20373600 0.55485200
C 0.34814700 -3.43410900 1.14746600
C 1.57673200 -3.63091100 1.77619000
C 2.51803800 -2.60458800 1.76524000
C 2.27455800 -1.36847800 1.14049500
C 3.39133200 -0.33913200 1.14034000
C -1.04890800 1.34394400 2.08513200
C -1.50290700 0.05970200 2.81475000
C 0.33012600 1.75045000 2.63946900
C -2.07692900 2.44945500 2.39799900
C -0.87258000 2.56697600 -0.83657700
C -0.47632900 2.14268200 -2.27092200
C 0.16648200 3.57885800 -0.31960800
C -2.25482700 3.24831300 -0.93219800
C 5.07460600 -1.45409400 -1.10566100
C 3.99330500 -2.35197600 -1.75434600
C 5.78747800 -2.25423400 -0.00068300
C 6.09392500 -1.09144200 -2.20460700
C 5.39472000 1.49517000 -0.00803800
C 6.59379500 1.03370900 0.83841700
C 5.90065000 2.19738000 -1.28844800
C 4.60346900 2.55147000 0.79796000
H 0.66607200 0.00577900 -1.20561700
H 1.41509900 0.91500000 0.07099000
H -0.90390600 -2.04421400 0.09677600
H -0.39858200 -4.22327200 1.12759100
H 1.80475600 -4.57328200 2.26692400
H 3.47530800 -2.76023100 2.25634100
H 4.17843700 -0.66858900 1.82759900
H 3.01735300 0.60934900 1.53791900
H -2.49728100 -0.25668900 2.48997100
H -0.81444500 -0.77350300 2.65406100
H -1.54306700 0.25979700 3.89428700
H 1.05218300 0.93389500 2.55043500
H 0.74441300 2.63671800 2.15237400
H 0.23237300 1.97941300 3.70925300
H -2.19012300 2.53131200 3.48705700
H -1.76577600 3.43110600 2.02956900
H -3.06383800 2.22044800 1.98168600
H -1.13175400 1.35097500 -2.65454500
H -0.58140600 3.00828700 -2.93774900
H 0.56191400 1.80466300 -2.33949100
H 1.16393900 3.13843100 -0.21427200
H 0.25306300 4.40472500 -1.03856000
H -0.11920300 4.01725600 0.64111900
H -2.62020200 3.60743100 0.03275800
H -2.18082800 4.11758600 -1.59961300
H -3.00136600 2.56309900 -1.34479900
H 4.46374400 -3.26417600 -2.14694700
H 3.22343300 -2.65772700 -1.04051400
H 3.50009700 -1.84243200 -2.58947700
H 6.55688400 -1.67141800 0.51226900
H 5.08516700 -2.62898500 0.74808000
H 6.28088800 -3.13045600 -0.44457000
H 6.45752500 -2.01305500 -2.67891600
H 5.64705500 -0.47170500 -2.99055900
H 6.96793500 -0.56644300 -1.80682400
H 7.17565300 1.90671800 1.16707700
H 6.28107800 0.49423900 1.73954600
H 7.27331400 0.38828800 0.27400700
H 5.06637000 2.57360900 -1.89097600
H 6.53049300 3.05469700 -1.01365200
H 6.50136000 1.54065900 -1.92173900
H 3.67832100 2.84821400 0.28807300
H 4.34774100 2.20719900 1.80490200
H 5.21738600 3.45463400 0.91226800
B -4.54097600 -0.23422000 0.12659100

C	-5.42183900	1.03428100	-0.26502000
C	-6.92771200	1.01043200	0.07149500
H	-5.28754600	1.29856100	-1.32124500
H	-4.96010200	1.86985400	0.29124900
H	-7.39530500	1.98308400	-0.12742100
H	-7.45951400	0.26562800	-0.53168700
H	-7.10730700	0.76897900	1.12566600
C	-4.92579600	-1.21780000	1.32012600
C	-6.01678100	-2.25298500	0.95215500
H	-5.29869000	-0.61300300	2.16520300
H	-4.05237100	-1.76548600	1.69320600
H	-6.25402100	-2.89006100	1.81307000
H	-6.94674400	-1.77283100	0.63132600
H	-5.68338900	-2.90653800	0.13950100
C	-3.86057700	-1.76770900	-1.98066800
C	-5.09323700	-1.35225800	-2.79071600
H	-4.05542700	-2.71304700	-1.45832300
H	-3.02747700	-1.97287600	-2.68003000
H	-5.36436000	-2.10794000	-3.54472900
H	-5.97566800	-1.21217500	-2.15269500
H	-4.93076400	-0.40649300	-3.32230500

39-ts

Zero-point correction= 0.830785 (Hartree/Particle)

Thermal correction to Energy= 0.877146

Thermal correction to Enthalpy= 0.878091

Thermal correction to Gibbs Free Energy= 0.751576

E(Solv) = -2014.83434712

Pd	-3.33339800	0.01388900	0.54823100
P	4.77040400	-0.51017800	0.38814700
P	-1.41422100	1.16076400	-0.20918300
C	3.16555700	0.49166000	0.58940700
C	2.11702600	-0.13991900	1.49462000
C	2.53645600	-0.55818200	2.77265400
C	1.66931600	-1.15988800	3.67733500
C	0.33742900	-1.36669700	3.31404100
C	-0.10329500	-0.93851400	2.06683900
C	0.75324900	-0.30409300	1.14773800
C	0.17466800	0.13382100	-0.18970000
C	4.23852000	-1.94798300	-0.78335400
C	3.61397200	-3.01424500	0.14840900
C	3.21590900	-1.59460400	-1.87812700
C	5.48528400	-2.57648800	-1.43733400
C	5.86227900	0.76003500	-0.57323300
C	5.90208000	2.05058200	0.27874100
C	5.41690400	1.12632300	-1.99959200
C	7.30301200	0.20143200	-0.60524800
C	-1.51214200	1.62217700	-2.08836000
C	-1.27740300	0.33182000	-2.90634900
C	-0.49246500	2.66416800	-2.58870000
C	-2.93650000	2.14068200	-2.38081500
C	-0.95870100	2.74643600	0.81319000
C	0.45867400	3.28808800	0.52933100
C	-2.00797800	3.84173500	0.53602000
C	-1.01454200	2.41712200	2.32204800
H	3.49333700	1.42756600	1.05452400
H	2.76047600	0.76194100	-0.38778500
H	3.57953500	-0.42067500	3.04388900
H	2.03285300	-1.47261900	4.65263000
H	-0.35608700	-1.84768800	3.99883700
H	-1.14680600	-1.07237300	1.79336400
H	-0.08752700	-0.75570600	-0.77165600
H	0.91377300	0.68279100	-0.77602700
H	4.32287300	-3.33822300	0.91799700
H	2.71510200	-2.64874600	0.65296000
H	3.33067500	-3.89575300	-0.44353200
H	2.26955500	-1.24904500	-1.45234500
H	3.58148900	-0.82793100	-2.56684500
H	2.99131800	-2.49085600	-2.47354700
H	5.20512400	-3.52535400	-1.91467200
H	5.91635500	-1.93833300	-2.21504400
H	6.26632900	-2.79749000	-0.70077700
H	6.09818100	1.83795100	1.33681700
H	6.71139400	2.69891000	-0.08241700
H	4.97417100	2.62714900	0.21019700
H	4.38642800	1.49868600	-2.02784100
H	6.06129100	1.92266500	-2.39896500
H	5.49071000	0.27767000	-2.68639500
H	7.39192000	-0.70566900	-1.20750600
H	7.97902300	0.95117900	-1.03895600
H	7.66127400	-0.02894900	0.40425200
H	-1.56956400	0.51444400	-3.94859300
H	-0.22385200	0.03471300	-2.91136600
H	-1.86979900	-0.50981000	-2.54078700
H	-0.66884200	3.66011200	-2.17505600
H	0.54317700	2.37758500	-2.37664100
H	-0.58580000	2.74784600	-3.67981900
H	-3.04408900	2.31643400	-3.45984700

H	-3.69530400	1.41710100	-2.07458100
H	-3.14624900	3.08473000	-1.87084300
H	0.63080900	4.16455900	1.16809400
H	1.22834900	2.55360900	0.78184900
H	0.60930800	3.60722700	-0.50199600
H	-3.02541800	3.46891000	0.69791900
H	-1.84552200	4.67695900	1.22985600
H	-1.94118800	4.24613600	-0.47812600
H	-1.99019400	2.02113100	2.61326500
H	-0.24834500	1.69822900	2.61917100
H	-0.84016400	3.34547400	2.88260300
B	-3.62884600	-1.50484300	-0.91062000
C	-3.01621800	-2.97513100	-0.94462600
C	-1.93085700	-3.39716200	0.05341000
H	-2.66773000	-3.17977900	-1.97316400
H	-3.88044700	-3.65458000	-0.83146800
H	-1.65886900	-4.45501100	-0.06025200
H	-1.01493700	-2.80919900	-0.07042800
H	-2.26470600	-3.25082400	1.08752800
C	-4.97379400	-1.21926300	1.25246400
C	-4.87301500	-0.09986900	2.13310400
H	-4.62754500	-2.18773200	1.60331500
H	-5.80927100	-1.25359000	0.55809500
H	-5.61737200	0.68871700	2.07359300
H	-4.45659900	-0.24328200	3.12586800
H	-3.57383300	1.13444800	1.77754300
C	-4.68300600	-1.17579700	-2.08191600
C	-5.64993600	-2.30905800	-2.49171300
H	-4.08709200	-0.90627700	-2.97081000
H	-5.27195400	-0.27564900	-1.85716000
H	-5.11178000	-3.19307600	-2.85102500
H	-6.27395800	-2.63020900	-1.64742300
H	-6.32908700	-1.98902700	-3.29213500

40

E(Solv)=-1936.26758189

Zero-point correction= 0.779561 (Hartree/Particle)

Thermal correction to Energy= 0.822835

Thermal correction to Enthalpy= 0.823779

Thermal correction to Gibbs Free Energy= 0.706959

Pd	0.39091900	-0.97360600	-0.16611000
P	1.60515800	0.92631000	0.40322900
P	-1.96807800	-0.57679500	0.39039200
C	0.48808000	2.33604300	1.04509300
C	-0.67266600	2.89567400	0.24204700
C	-0.45548800	4.10235800	-0.44774400
C	-1.46604100	4.77388000	-1.12967500
C	-2.75957600	4.25772500	-1.10209900
C	-3.00325900	3.08195900	-0.39805400
C	-1.98855400	2.36787100	0.26383400
C	-2.39498800	1.14467600	1.06456400
C	2.64631200	1.73822800	-1.00995900
C	1.66964900	1.91892400	-2.19581400
C	3.29867500	3.08946400	-0.66317800
C	3.75132700	0.77211100	-1.48514600
C	2.68004100	0.71236300	2.01004400
C	1.81912200	-0.11559000	2.98961300
C	3.09135300	2.01814800	2.72199000
C	3.95398900	-0.10056800	1.70628400
C	-3.07942900	-0.79473200	-1.17287600
C	-2.34687900	-0.05204100	-2.31545900
C	-4.51625700	-0.24862300	-1.07180900
C	-3.14194100	-2.28864300	-1.55348400
C	-2.63968500	-1.68739200	1.82933800
C	-4.17032400	-1.72257400	1.99236600
C	-2.11256100	-3.12411100	1.62016400
C	-2.02606200	-1.16966000	3.15017600
H	0.10988600	1.92809700	1.98863500
H	1.13468800	3.17534400	1.31690800
H	0.53991800	4.53751300	-0.43088000
H	-1.24750800	5.70163400	-1.65131500
H	-3.57602700	4.77373500	-1.59980000
H	-4.02323600	2.71447200	-0.33438300
H	-3.46829000	1.20298000	1.27900900
H	-1.88620500	1.17113300	2.03270700
H	1.22190200	0.96242100	-2.48290000
H	0.86057300	2.61843300	-1.97985700
H	2.22638300	2.30577900	-3.06031100
H	2.58770300	3.82556100	-0.27685900
H	4.10378600	2.97898200	0.06966600
H	3.74655600	3.51785000	-1.57017500
H	4.14985700	1.13576000	-2.44186400
H	4.58917500	0.71219300	-0.78777200
H	3.36394600	-0.23602700	-1.64811900
H	1.52099300	-1.07122400	2.54741700
H	2.39965100	-0.31946900	3.89974100
H	0.91159900	0.41490700	3.29759700
H	2.23247900	2.57869800	3.10320300

H	3.71769000	1.76589200	3.58852700
H	3.67692200	2.68432400	2.08312300
H	4.71731600	0.50424400	1.20940000
H	4.38819400	-0.45265400	2.65168000
H	3.74618700	-0.97749900	1.08746000
H	-2.90545500	-0.19083300	-3.25168600
H	-2.26809100	1.02237400	-2.12909500
H	-1.33519000	-0.44611800	-2.45849000
H	-5.10617700	-0.74154000	-0.29626300
H	-4.53709300	0.82682500	-0.88433500
H	-5.02998000	-0.41599200	-2.02854200
H	-3.58004600	-2.38252700	-2.55627300
H	-2.14682000	-2.74484900	-1.58045400
H	-3.77177400	-2.86730400	-0.87077900
H	-4.42561200	-2.27819300	2.90546000
H	-4.60483600	-0.72145100	2.09093600
H	-4.66440800	-2.23061100	1.15945100
H	-1.02044000	-3.13984000	1.55114800
H	-2.41241500	-3.74688600	2.47426700
H	-2.50874600	-3.59278900	0.71660600
H	-0.94363600	-1.02937500	3.06884200
H	-2.47773500	-0.22991500	3.48406600
H	-2.20499700	-1.91033300	3.94065900
B	1.69996700	-2.43411700	-1.20474900
C	2.85001900	-3.30106800	-0.51927300
C	2.59475300	-3.86310800	0.88696800
H	3.77266200	-2.69511200	-0.51465700
H	3.09269100	-4.13765500	-1.19886400
H	3.44658800	-4.44271400	1.26705200
H	2.39218200	-3.05948900	1.60472900
H	1.71856400	-4.52393900	0.89123300
H	0.48769800	-2.65286000	-0.76964400
C	1.81341700	-2.21177000	-2.78843100
C	1.25171900	-3.43750400	-3.54997300
H	2.86242200	-2.07090500	-3.09279200
H	1.26599500	-1.32325300	-3.13102800
H	1.78488400	-4.35817400	-3.28284800
H	0.19067700	-3.59668800	-3.31934900
H	1.33815900	-3.31144400	-4.63674000

41

Zero-point correction= 0.492654 (Hartree/Particle)

Thermal correction to Energy= 0.523047

Thermal correction to Enthalpy= 0.523991

Thermal correction to Gibbs Free Energy= 0.427738

E(Solv) = -1759.16502557

C	-3.06292700	2.69480400	-0.56810800
O	-1.71319100	3.22778000	-0.53333200
C	-0.86104700	2.19956700	-0.67633500
C	-2.87597500	1.20781300	-0.96610000
H	-3.49213200	2.81655700	0.42945600
H	-3.63583700	3.27743700	-1.29091900
H	-3.16662900	1.05130600	-2.01118100
N	-1.39746300	1.04227100	-0.89945000
C	0.56950700	2.56640800	-0.59776100
C	0.87712100	3.91385200	-0.84995700
C	1.61492900	1.65918800	-0.28483600
C	2.19327300	4.36969200	-0.81928900
H	0.07328700	4.60284000	-1.07959500
C	2.92910400	2.14251700	-0.24926500
C	3.22232300	3.48197600	-0.51833200
H	2.40722800	5.41291100	-1.02943300
H	3.74025400	1.46352500	-0.01042500
H	4.25293000	3.82280600	-0.48884400
P	1.30012900	-0.11683400	0.11714200
Pd	-0.46680400	-0.85073300	-1.20434300
C	-3.64714600	0.23409600	-0.09799600
C	-3.27765500	0.00058600	1.23441300
C	-4.77690800	-0.41165500	-0.61471100
C	-4.02272100	-0.87036800	2.03003800
H	-2.40286500	0.49227100	1.65197600
C	-5.52677000	-1.27882600	0.18326300
H	-5.07660200	-0.23312000	-1.64525000
C	-5.14892000	-1.51165500	1.50684300
H	-3.72524000	-1.04438600	3.06022500
H	-6.40367200	-1.76940100	-0.22904300
H	-5.73077500	-2.18543200	2.12893700
C	0.94074200	-0.14876600	1.91645200
C	1.34993600	0.87669600	2.78393900
C	0.24967400	-1.25593800	2.43817500
C	1.07365000	0.79134500	4.14994500
H	1.88367800	1.74024000	2.39958300
C	-0.01523500	-1.34078000	3.80503600
H	-0.08120600	-2.05053000	1.77500100
C	0.39434800	-0.31603600	4.66196600
H	1.39246400	1.59016700	4.81314600
H	-0.54384300	-2.20402100	4.19898400
H	0.18347000	-0.37985500	5.72547100

C	2.92200600	-0.94377000	-0.10288800
C	3.37587200	-1.18571400	-1.41203200
C	3.70843300	-1.35178200	0.98414000
C	4.60056200	-1.81500000	-1.62692600
H	2.77299900	-0.87630900	-2.26274900
C	4.93077200	-1.99209300	0.76240600
H	3.37291300	-1.17400700	2.00068300
C	5.37848000	-2.22255700	-0.53861400
H	4.94540800	-1.99285600	-2.64140100
H	5.53146800	-2.30822800	1.61019400
H	6.32897900	-2.72023900	-0.70674200
C	-1.97680900	-1.97716100	-2.46311300
H	-2.58351600	-2.35455300	-1.64260400
C	-0.68423400	-2.46124100	-2.68619200
C	0.10348800	-2.87966600	-1.57796900
H	-0.19726700	-2.25700000	-3.63837800
H	-2.51398700	-1.47976700	-3.26511500
H	1.15323800	-3.11284400	-1.72862900
H	-0.37313300	-3.41261900	-0.75501500

42-ts

Zero-point correction= 0.677584 (Hartree/Particle)
Thermal correction to Energy= 0.716921
Thermal correction to Enthalpy= 0.717865
Thermal correction to Gibbs Free Energy= 0.598111
SCF Done: E(RB3LYP) = -2163.64578385

C	-4.84360600	-1.54162100	-1.27620200
O	-4.76902600	-0.10192800	-1.42753400
C	-3.47828700	0.25593600	-1.26115800
C	-3.36181700	-1.99756100	-1.25488500
H	-5.36414300	-1.75191000	-0.33775700
H	-5.41827600	-1.94071200	-2.11341800
H	-3.07770900	-2.42652800	-2.22305200
N	-2.63158400	-0.71082700	-1.12530100
C	-3.22843000	1.71395300	-1.31443900
C	-4.20645700	2.48159700	-1.96922400
C	-2.08927800	2.35738800	-0.76060500
C	-4.06716000	3.86039700	-2.10981200
H	-5.07755200	1.98368600	-2.37785500
C	-1.97771800	3.74586900	-0.90714800
C	-2.94766800	4.49435100	-1.57902000
H	-4.83079600	4.42963100	-2.63077100
H	-1.11679700	4.25633100	-0.48989100
H	-2.82374800	5.56876400	-1.67892500
P	-0.77749800	1.41778900	0.15643100
Pd	-0.47054100	-0.62665400	-0.98113800
C	-3.04139700	-3.00604700	-0.17030500
C	-3.03752800	-2.63869400	1.18258200
C	-2.79010700	-4.33984100	-0.51282600
C	-2.78308700	-3.58943200	2.17124800
H	-3.22310800	-1.60617300	1.46574800
C	-2.54086000	-5.29411300	0.47658400
H	-2.79441900	-4.63648400	-1.55953800
C	-2.53560900	-4.91994200	1.82137000
H	-2.78383100	-3.29100700	3.21601800
H	-2.35553000	-6.32713200	0.19588600
H	-2.34624000	-5.66085400	2.59297100
C	-1.46013500	1.28101500	1.86303100
C	-2.42729900	2.16125600	2.37418600
C	-0.97560700	0.25101000	2.68651600
C	-2.89319500	2.01606600	3.68281100
H	-2.81948800	2.96119900	1.75362500
C	-1.43561400	0.11373700	3.99666600
H	-0.24203600	-0.44856400	2.29443300
C	-2.39694600	0.99554100	4.49639700
H	-3.64307900	2.70247400	4.06573200
H	-1.04910800	-0.68457000	4.62433700
H	-2.76036100	0.88578700	5.51415600
C	0.61748800	2.61131800	0.29866100
C	1.51647000	2.69536300	-0.77887600
C	0.83145900	3.41474200	1.42892500
C	2.59617300	3.57662700	-0.73355300
H	1.36433700	2.06924600	-1.65513100
C	1.92156500	4.28744700	1.47618900
H	0.15141000	3.36111100	2.27323000
C	2.80210300	4.37258500	0.39661500
H	3.28018600	3.63705900	-1.57542000
H	2.07874700	4.90254500	2.35779500
H	3.64725800	5.05385100	0.43590100
C	0.29015000	-2.34075300	-1.98312800
H	0.09821000	-3.20719200	-1.35001500
C	1.37956500	-1.46633900	-1.68566000
C	2.02550800	-1.44011600	-0.41606200
H	1.79749200	-0.84336400	-2.47467200
H	-0.01781000	-2.45988000	-3.01940200
H	2.59057900	-0.55816900	-0.13677700
H	1.59378000	-2.00369000	0.40514900
C	4.68549100	-2.09968400	-1.40057700

C	5.49646900	-1.00069300	-0.74458200
C	5.54421700	-0.86582500	0.65164800
C	4.71759300	-1.76719900	1.55038100
C	4.21295400	-3.01630500	0.82701500
H	6.20934800	-0.24797000	-2.63482600
H	5.34850700	-2.91618800	-1.72697100
C	6.24745000	-0.13659800	-1.55318700
C	6.34739300	0.13855400	1.21000800
H	5.30696400	-2.07967000	2.42007600
H	3.42690700	-3.51456600	1.40303700
C	7.09451200	0.99213600	0.40301400
C	7.04482800	0.85419400	-0.98737900
H	6.38743600	0.24464900	2.29174900
H	7.71658000	1.75940400	0.85460000
H	7.62490800	1.51456900	-1.62521300
N	3.65519400	-2.64427100	-0.49317000
H	3.22373800	-3.45726300	-0.93234700
H	4.17613100	-1.72766300	-2.29666700
H	5.03218800	-3.73637900	0.68673300
H	3.86214200	-1.20592800	1.95161800

43

Zero-point correction= 0.680545 (Hartree/Particle)

Thermal correction to Energy= 0.719663

Thermal correction to Enthalpy= 0.720607

Thermal correction to Gibbs Free Energy= 0.601907

C	-4.65339800	-2.12793600	-1.10293300
O	-4.81403600	-0.69462500	-1.22933100
C	-3.58310900	-0.14153500	-1.12169100
C	-3.11904100	-2.34339100	-1.17409300
H	-5.07799500	-2.42964900	-0.14097700
H	-5.20628300	-2.60484900	-1.91397500
H	-2.82699700	-2.70788800	-2.16630100
N	-2.59064800	-0.96330700	-1.05214300
C	-3.56570600	1.33865800	-1.15745400
C	-4.69658100	1.95176500	-1.72381300
C	-2.50089200	2.14615400	-0.67364100
C	-4.78408700	3.33630300	-1.84556900
H	-5.50783800	1.32776400	-2.07883700
C	-2.61849200	3.53658800	-0.80045200
C	-3.73937900	4.13177300	-1.38446400
H	-5.66369100	3.78417300	-2.29777100
H	-1.81860800	4.17160400	-0.43614400
H	-3.79060100	5.21342500	-1.47040500
P	-0.99197800	1.41221300	0.12900500
Pd	-0.43175300	-0.55597200	-1.03218300
C	-2.58401900	-3.30725100	-0.13411400
C	-2.54249700	-2.95919900	1.22311600
C	-2.16812800	-4.58711400	-0.51901900
C	-2.09224400	-3.87533900	2.17393800
H	-2.85130400	-1.96612000	1.53753300
C	-1.72195800	-5.50779500	0.43242400
H	-2.19810800	-4.86821900	-1.56958700
C	-1.68251400	-5.15310600	1.78198400
H	-2.06787200	-3.59216400	3.22281500
H	-1.41216200	-6.50101000	0.11893600
H	-1.34246600	-5.86929700	2.52497000
C	-1.53116400	1.18686900	1.88143000
C	-2.55458400	1.94172200	2.47701100
C	-0.87493000	0.21145300	2.65032100
C	-2.90596600	1.72960300	3.81195800
H	-3.08141600	2.69587400	1.90025500
C	-1.22103400	0.00653400	3.98673300
H	-0.09882200	-0.39486500	2.19043600
C	-2.23879900	0.76494100	4.56972100
H	-3.70134800	2.31940100	4.25901600
H	-0.70258500	-0.74969000	4.57022600
H	-2.51411600	0.60203800	5.60791000
C	0.19410100	2.82446100	0.19243200
C	0.96150000	3.07563500	-0.95865200
C	0.38504900	3.63130700	1.32408300
C	1.88280700	4.12204300	-0.98341100
H	0.82925400	2.44753600	-1.83669000
C	1.32084300	4.66914200	1.30203200
H	-0.19474000	3.45332400	2.22425800
C	2.06706400	4.91955100	0.14967200
H	2.45992400	4.31297400	-1.88424900
H	1.46053700	5.28477900	2.18638700
H	2.78948000	5.73081600	0.13381500
C	0.67769900	-2.03874000	-2.06006500
H	0.63092700	-2.99557400	-1.53552500
C	1.53796100	-0.99242300	-1.58202500
C	2.44861300	-1.24378500	-0.42169000
H	1.90294700	-0.25312300	-2.29673800
H	0.43290900	-2.09749300	-3.11898700
H	2.86333500	-0.33542200	0.01814600
H	1.96620900	-1.84373000	0.35312200
C	4.70453100	-1.33643100	-1.64705300

C	5.58001300	-0.48574900	-0.75363000
C	5.74302000	-0.77798200	0.60918400
C	4.95548100	-1.89551400	1.26742500
C	4.34345200	-2.86432300	0.26045300
H	6.14643100	0.80727800	-2.38476100
H	5.29493800	-2.07748100	-2.19943900
C	6.27917800	0.58384000	-1.32865500
C	6.61200700	0.01206400	1.37321600
H	5.59959600	-2.47461900	1.93890700
H	3.58686400	-3.50701100	0.71730600
C	7.31262800	1.06822000	0.79676500
C	7.14576900	1.35644800	-0.56078400
H	6.73880400	-0.20811600	2.43046600
H	7.98642500	1.66586600	1.40322300
H	7.68521900	2.18094100	-1.01666800
N	3.67712800	-2.11098100	-0.86382800
H	3.25743700	-2.79255300	-1.50620200
H	4.16103900	-0.73421500	-2.37870400
H	5.10850000	-3.49814800	-0.19898500
H	4.16424900	-1.47278500	1.90087200

44

Zero-point correction= 0.259826 (Hartree/Particle)
Thermal correction to Energy= 0.271248
Thermal correction to Enthalpy= 0.272192
Thermal correction to Gibbs Free Energy= 0.222073
E(Solv) = -521.702205814

C	-4.03361600	-1.30416800	-0.71079400
C	-3.46397800	-0.12005300	-0.47322600
H	-4.00080300	-2.11811500	0.01034900
H	-3.55211000	0.68076900	-1.20458600
C	-2.77348000	0.20556300	0.81679700
H	-2.88216700	-0.60317900	1.54425500
C	-0.51628300	-0.81282900	0.25325300
C	0.96748100	-0.55381600	0.11146200
C	1.47390200	0.73893300	-0.09134300
C	0.55689600	1.94858400	-0.08386100
C	-0.91181600	1.57953200	-0.26272000
H	1.44018500	-2.65118400	0.31018500
H	-0.72206400	-1.57265400	1.01318900
C	1.83749300	-1.65181400	0.14879000
C	2.85482800	0.90805300	-0.25547300
H	0.67989900	2.51466900	0.85135100
H	-1.12648500	1.22765500	-1.27422200
C	3.71663400	-0.18525600	-0.22641200
C	3.20675500	-1.47095600	-0.02367300
H	3.25415200	1.90755300	-0.40822600
H	4.78360800	-0.03620900	-0.35999000
H	3.87354800	-2.32693000	0.00518300
N	-1.26429600	0.43450400	0.66638200
H	-0.98260900	-1.13987300	-0.68148200
H	-1.57756300	2.41354200	-0.02678700
H	0.82638400	2.64275900	-0.88785700
H	-4.57581300	-1.49507400	-1.63195100
H	-3.15817900	1.12980300	1.26117300
H	-0.91880900	0.69431200	1.59793900

45-ts

Zero-point correction= 0.843033 (Hartree/Particle)
Thermal correction to Energy= 0.892898
Thermal correction to Enthalpy= 0.893842
Thermal correction to Gibbs Free Energy= 0.747414
E(Solv) = -2550.86641313

C	4.50694600	3.51732300	1.52988000
O	5.27821800	2.79211100	0.54099200
C	4.47317700	1.84453300	0.01104200
C	3.06365900	2.96783200	1.36598400
H	4.93502600	3.29966600	2.51206300
H	4.59991900	4.58354400	1.31526300
H	2.42423100	3.70471000	0.86683800
N	3.24825100	1.84292400	0.41870200
C	5.12585200	0.97854900	-0.99790000
C	6.30510600	1.48692600	-1.56993900
C	4.64376600	-0.29393900	-1.40864100
C	6.99974100	0.77890800	-2.54737500
H	6.67021000	2.45328100	-1.24428300
C	5.36698900	-0.98957400	-2.38694700
C	6.52804500	-0.46415900	-2.95861300
H	7.90202600	1.20005000	-2.98012100
H	5.01748900	-1.96326000	-2.71181900
H	7.05788900	-1.03155800	-3.71835400
P	3.11072000	-1.05525600	-0.68934800
Pd	1.54788900	0.66723000	-0.30220300
C	2.41097600	2.56982300	2.67560600
C	2.74734100	1.37403800	3.32440600
C	1.49572700	3.43807200	3.28336400
C	2.17376400	1.05331200	4.55532700
H	3.44890600	0.68558200	2.86215000

C	0.92637800	3.12124300	4.51883000
H	1.22952700	4.36919800	2.78811800
C	1.26335500	1.92620100	5.15696100
H	2.44224000	0.12123800	5.04505700
H	0.22252800	3.80783600	4.98117900
H	0.82287700	1.67816400	6.11862100
C	3.71919200	-1.91037000	0.82649600
C	5.06414400	-2.25673000	1.03061100
C	2.77636800	-2.22899800	1.81882700
C	5.45514500	-2.91122800	2.20111400
H	5.80950200	-2.01742800	0.27832700
C	3.16793600	-2.89224700	2.98214500
H	1.73530700	-1.95016800	1.67825000
C	4.50912400	-3.23218400	3.17644000
H	6.49966300	-3.17134700	2.34824100
H	2.42759900	-3.13714000	3.73888700
H	4.81606400	-3.74226600	4.08516100
C	2.70090300	-2.41661500	-1.86048000
C	1.96396700	-2.08474100	-3.01059700
C	3.06258200	-3.75520700	-1.64725600
C	1.61771400	-3.06636200	-3.93857500
H	1.66596300	-1.05194800	-3.17632900
C	2.70130900	-4.73906800	-2.57140300
H	3.62744400	-4.03252500	-0.76277400
C	1.98407900	-4.39720100	-3.71890300
H	1.05866100	-2.79454600	-4.82993300
H	2.98714300	-5.77231600	-2.39479000
H	1.70985500	-5.16370800	-4.43832400
C	-0.19758300	1.92985700	-0.23252300
C	-0.53021600	0.71411400	-0.91447600
H	-0.46477800	1.98197800	0.82463300
H	-0.64859300	0.71119000	-1.99727100
C	-0.84131300	-0.48077600	-0.21185900
C	-1.31643800	-1.68847300	-0.76126400
H	-0.82605500	-0.40742400	0.87778400
C	-0.10912600	3.24764600	-0.89426100
C	-0.37488800	4.41312000	-0.15008800
C	0.24107700	3.40006700	-2.25094900
C	-0.29688500	5.67780100	-0.73354600
H	-0.65761900	4.31812300	0.89612400
C	0.31453900	4.66273600	-2.83469300
H	0.47578300	2.52263800	-2.84769100
C	0.04728200	5.80933700	-2.08022400
H	-0.51263900	6.56055800	-0.13739600
H	0.58699100	4.75408600	-3.88280200
H	0.10693000	6.79249000	-2.53800200
H	-1.18519500	-2.57973900	-0.14529700
H	-1.11999700	-1.86299300	-1.82176100
C	-6.48350200	1.03688500	-0.61098500
C	-5.96854100	-0.01664400	-1.26894200
H	-5.83622584	1.70935200	-0.08780079
H	-6.61917900	-0.69109300	-1.82354800
C	-4.49144400	-0.29179600	-1.36550900
H	-3.95935761	0.54067852	-0.95469243
C	-4.30725200	-1.42578800	0.83884500
C	-3.99186500	-2.68843100	1.61463300
C	-3.88028300	-3.93398800	0.98156500
C	-3.99899300	-4.03662300	-0.52418200
C	-4.67880700	-2.81550700	-1.14327300
H	-3.94117200	-1.63326400	3.49498700
H	-3.67807000	-0.59829200	1.18762100
C	-3.85096000	-2.60143300	3.00671700
C	-3.62870800	-5.07392100	1.75752400
H	-3.00363600	-4.16434600	-0.97051800
H	-5.74915600	-2.80607300	-0.90550100
C	-3.49202000	-4.98377100	3.14001300
C	-3.60408200	-3.73993500	3.76873500
H	-3.54336200	-6.04085300	1.26692000
H	-3.30282200	-5.87814600	3.72644700
H	-3.50028700	-3.65932700	4.84681700
N	-4.06880500	-1.55711600	-0.62481500
H	-5.35074000	-1.12316100	0.99013100
H	-4.57007400	-2.82969600	-2.22899000
H	-4.57418500	-4.92758400	-0.80233700
H	-2.78415493	-1.60580338	-0.72538329
H	-7.54056408	1.20277537	-0.61059264
H	-4.22770819	-0.39354298	-2.39749300

46

Zero-point correction= 0.244126 (Hartree/Particle)
Thermal correction to Energy= 0.255547
Thermal correction to Enthalpy= 0.256491
Thermal correction to Gibbs Free Energy= 0.206354
E(Solv) = -521.239340703

C	-4.16303100	-1.29354500	-0.55062800
C	-3.46541700	-0.15935300	-0.47161100
H	-4.23170900	-1.97932300	0.29251400
H	-3.42467600	0.50022300	-1.33936700

C	-2.70844300	0.29895500	0.75767200
H	-2.86694400	-0.42512700	1.56793300
C	-0.55564500	-0.74805200	0.28174800
C	0.94029000	-0.54228600	0.13263200
C	1.48412300	0.74073400	-0.03357000
C	0.58029100	1.95633900	-0.01095400
C	-0.87733700	1.58024300	-0.28808700
H	1.36444800	-2.64998900	0.25064500
H	-0.74758100	-1.46890900	1.08831800
C	1.79022100	-1.65632100	0.12281300
C	2.86861800	0.87801900	-0.20534200
H	0.63432300	2.43493500	0.97658700
H	-0.99673300	1.32223700	-1.35869200
C	3.70857300	-0.23307900	-0.21090200
C	3.16504900	-1.51006700	-0.04556800
H	3.28803200	1.87363800	-0.33716700
H	4.77948200	-0.10584700	-0.34591400
H	3.80935600	-2.38531800	-0.04694400
N	-1.26325700	0.48794800	0.60135100
H	-0.94300200	-1.21730000	-0.64497800
H	-1.52943800	2.43859900	-0.08825900
H	0.92372100	2.69801500	-0.74388000
H	-4.70043200	-1.57970300	-1.45130000
H	-3.11980600	1.25870900	1.10182800

47-ts

Zero-point correction=0.783267 (Hartree/Particle)

Thermal correction to Energy= 0.828328

Thermal correction to Enthalpy=0.829273

Thermal correction to Gibbs Free Energy= 0.699822

E(Solv)= -2434.11589350

C	-3.96346400	1.38038500	2.07438400
O	-2.78209000	1.84264300	2.77357200
C	-1.71355400	1.31772700	2.13494200
C	-3.41895200	0.31289000	1.08069200
H	-4.65877700	0.97886300	2.81304700
H	-4.41063500	2.24332500	1.57364000
H	-3.72689100	0.54839200	0.05737700
N	-1.94763800	0.48600500	1.17753700
C	-0.40554800	1.75373000	2.67705800
C	-0.35155600	2.00898000	4.05590000
C	0.75342800	1.93929300	1.88131000
C	0.83810900	2.40296300	4.66562000
H	-1.25231200	1.89197400	4.64809800
C	1.93410500	2.35014800	2.51251800
C	1.98353000	2.57065000	3.89145900
H	0.86301000	2.58380000	5.73584500
H	2.82811300	2.50938800	1.92010100
H	2.91580700	2.88606100	4.35093800
P	0.71677500	1.63959100	0.05325100
Pd	-0.29169400	-0.47175600	-0.30282700
C	-3.87523600	-1.09753200	1.41752400
C	-3.34930000	-1.78950000	2.51706300
C	-4.90131900	-1.69385900	0.67291900
C	-3.83508300	-3.05268400	2.85837600
H	-2.55640000	-1.34012100	3.10915100
C	-5.38792700	-2.95916500	1.01107600
H	-5.33436800	-1.15823200	-0.16918200
C	-4.85362600	-3.64269100	2.10466000
H	-3.42217000	-3.57500300	3.71711100
H	-6.18600500	-3.40562900	0.42468500
H	-5.23321900	-4.62422300	2.37348100
C	2.38851500	2.12384900	-0.53965700
C	2.67037400	3.41932800	-1.00332500
C	3.41408400	1.16490400	-0.52821800
C	3.95432900	3.74612200	-1.44262400
H	1.89079800	4.17323400	-1.02737200
C	4.70005700	1.50023400	-0.95362000
H	3.21248200	0.15545300	-0.18144900
C	4.97122100	2.79018000	-1.41518400
H	4.15801500	4.74996600	-1.80441200
H	5.48303300	0.74889000	-0.92715000
H	5.97013200	3.04840200	-1.75526800
C	-0.39868100	2.96490000	-0.57834300
C	-0.60450700	4.16965600	0.11359300
C	-1.04716500	2.76411100	-1.80734600
C	-1.44230500	5.15276700	-0.41716400
H	-0.11206900	4.34562100	1.06527200
C	-1.88009500	3.75098300	-2.33664000
H	-0.90699300	1.83383000	-2.35053300
C	-2.08076000	4.94571300	-1.64202900
H	-1.59283900	6.08110200	0.12667300
H	-2.37586100	3.57979300	-3.28789200
H	-2.73183200	5.71244600	-2.05228600
C	-0.74179200	-1.77591000	-2.07613000
C	-0.97722000	-2.50552900	-0.90952200
H	0.21699700	-1.87247600	-2.57580300
H	-2.00131100	-2.65629000	-0.57744300

C	0.01712400	-3.49495100	-0.30017100
C	-0.43083200	-3.86125800	1.12041600
H	0.00569900	-4.42268600	-0.89245400
H	0.19476300	-4.63570400	1.57052000
H	-0.45398600	-2.98686700	1.77748400
C	-1.75458500	-1.09582300	-2.90956600
C	-1.32101700	-0.40938200	-4.06018800
C	-3.13461200	-1.13000300	-2.63454100
C	-2.22934900	0.23240500	-4.90007000
H	-0.25911200	-0.38446400	-4.29489400
C	-4.04180100	-0.48768700	-3.47444600
H	-3.50310000	-1.67622000	-1.77240300
C	-3.59506300	0.19898500	-4.60758500
H	-1.87276200	0.74893800	-5.78664900
H	-5.10501200	-0.53544900	-3.25487500
H	-4.30723400	0.68961500	-5.26463500
H	-1.45095000	-4.25316700	1.06827100
C	2.27437600	-3.12691100	0.79749600
C	3.70525500	-2.66078200	0.59954000
C	4.25697400	-2.48694100	-0.67764600
C	3.38327300	-2.65326700	-1.90151600
C	2.13975900	-3.48009900	-1.59150400
H	4.07935200	-2.61399800	2.72312900
H	1.81849300	-2.58195400	1.62754100
C	4.50945100	-2.47584100	1.73320900
C	5.61199600	-2.14653000	-0.79645300
H	3.08100900	-1.66747100	-2.28325500
H	2.42011300	-4.52622500	-1.38783400
C	6.40913000	-1.97257900	0.33302500
C	5.85288900	-2.13281200	1.60579900
H	6.04331000	-2.02790900	-1.78800900
H	7.45988500	-1.72008900	0.22322600
H	6.46523000	-1.99759300	2.49243500
N	1.42595000	-2.94248500	-0.40252800
H	1.04109200	-1.43682500	-0.50052700
H	2.28782700	-4.19238500	1.07404300
H	1.45639600	-3.48998000	-2.44175100
H	3.94133000	-3.14202900	-2.70886000

48-ts

Zero-point correction= 0.596390 (Hartree/Particle)

Thermal correction to Energy= 0.632628

Thermal correction to Enthalpy= 0.633572

Thermal correction to Gibbs Free Energy=0.519804

E(Solv)= -2046.37642331

C	1.21412100	4.33335400	-0.04732000
O	2.18647500	3.56368200	0.69840400
C	1.69578500	2.29469700	0.77998600
C	0.16333500	3.28034600	-0.51447200
H	0.78618800	5.08443600	0.62329300
H	1.73504900	4.83066700	-0.86864500
H	0.24591500	3.10614400	-1.59428500
N	0.59566000	2.04657900	0.17039100
C	2.49522200	1.37426200	1.62158400
C	3.18075800	1.95465500	2.69994000
C	2.57919500	-0.02604700	1.40171100
C	3.91264900	1.17431100	3.59236100
H	3.12313300	3.02870500	2.83678800
C	3.32421100	-0.78764700	2.31241400
C	3.97669900	-0.20314300	3.40063200
H	4.42462400	1.64222500	4.42827200
H	3.40291300	-1.85914200	2.16783400
H	4.54024900	-0.82887400	4.08754500
P	1.71554400	-0.83716400	-0.03776400
Pd	-0.45055500	-0.17303400	-0.19363400
C	-1.26499700	3.68588600	-0.20320100
C	-1.77908400	3.58638900	1.09565400
C	-2.06909400	4.23005700	-1.21026300
C	-3.07270000	4.02365100	1.37926600
H	-1.17181000	3.14386800	1.88044400
C	-3.36299500	4.67286000	-0.92796400
H	-1.68314700	4.30282500	-2.22503800
C	-3.86788200	4.57101800	0.36895700
H	-3.46287700	3.93197100	2.38953800
H	-3.97634000	5.09026400	-1.72214100
H	-4.87633000	4.90968600	0.59084000
C	2.10934200	-2.63145700	0.17209700
C	3.33288100	-3.20652900	-0.20791000
C	1.11538800	-3.45379800	0.72505400
C	3.55844500	-4.57234300	-0.02833700
H	4.10856400	-2.59058700	-0.65327800
C	1.34641400	-4.81756400	0.91334400
H	0.15473000	-3.01783900	0.98672000
C	2.56764100	-5.37937400	0.53607300
H	4.50732600	-5.00619700	-0.33317600
H	0.56641300	-5.44169500	1.34119400
H	2.74414300	-6.44319100	0.67191800
C	2.78159400	-0.33403200	-1.46639400

C	4.10942600	0.10106200	-1.33360700
C	2.21721900	-0.40072500	-2.75142800
C	4.85606100	0.45594700	-2.46004000
H	4.56438300	0.16795700	-0.34966300
C	2.96700300	-0.05465400	-3.87595100
H	1.18283600	-0.71688200	-2.86021700
C	4.28813300	0.37637900	-3.73266000
H	5.88219600	0.79413800	-2.34061500
H	2.51615200	-0.11354400	-4.86317500
H	4.87014000	0.65319300	-4.60778300
C	-3.44761700	-0.27015100	0.67879000
C	-4.36007300	-1.48338200	0.52742400
C	-4.53937500	-2.09313900	-0.72647700
C	-3.78494400	-1.58248200	-1.94020100
C	-3.21760700	-0.17115200	-1.70829900
H	-4.92102900	-1.49854300	2.60515200
H	-2.86537500	-0.36273900	1.60200800
C	-5.06002400	-1.97693100	1.63637500
C	-5.41564500	-3.18248300	-0.83421300
H	-2.94873400	-2.26229600	-2.16334200
H	-4.04775700	0.55890400	-1.74243200
C	-6.10509300	-3.66994700	0.27434700
C	-5.92493600	-3.06344000	1.52058600
H	-5.55672200	-3.65103800	-1.80688700
H	-6.77992200	-4.51570700	0.16784000
H	-6.45367500	-3.43640200	2.39444200
N	-2.54034800	-0.02505700	-0.42986000
H	-1.55690100	-1.29434800	-0.42128300
H	-4.08758600	0.62460000	0.81450900
H	-2.52978200	0.09499800	-2.51753900
H	-4.44031200	-1.59536000	-2.82329300

49
Zero-point correction= 0.599046 (Hartree/Particle)
Thermal correction to Energy= 0.634786
Thermal correction to Enthalpy= 0.635730
Thermal correction to Gibbs Free Energy= 0.525400
E(Solv)=-2046.40272

C	-0.61481500	-3.95377100	1.42394500
O	0.54399800	-3.93817900	0.54962000
C	0.64481500	-2.66659100	0.08062800
C	-1.37735500	-2.66002700	1.05140300
H	-1.17098000	-4.87137000	1.22853900
H	-0.24951600	-3.94988800	2.45670400
H	-1.70758200	-2.09658300	1.92520100
N	-0.32672000	-1.87060600	0.36333600
C	1.81745400	-2.42199700	-0.79253500
C	2.32525400	-3.55349200	-1.45604800
C	2.39696400	-1.15053200	-1.03875000
C	3.35724500	-3.44466700	-2.38371200
H	1.88552900	-4.52152800	-1.24719700
C	3.43042700	-1.06785600	-1.98331900
C	3.90547900	-2.19393100	-2.65715800
H	3.72200400	-4.33234000	-2.89237200
H	3.87711600	-0.10232800	-2.19395300
H	4.70549300	-2.08911000	-3.38488900
P	1.86628600	0.36500400	-0.10197600
Pd	-0.34838400	0.36463900	0.48048700
C	-2.58397400	-2.91447700	0.16143200
C	-2.44334700	-3.31038900	-1.17493900
C	-3.87012600	-2.82253700	0.70627200
C	-3.56612000	-3.61150500	-1.94809100
H	-1.45482000	-3.37552600	-1.62161000
C	-4.99361800	-3.12843700	-0.06275800
H	-3.98976800	-2.48993500	1.73389000
C	-4.84459600	-3.52439200	-1.39355100
H	-3.44096100	-3.91209700	-2.98512600
H	-5.98509800	-3.04698800	0.37474300
H	-5.71862600	-3.75655400	-1.99615200
C	2.46692400	1.76274500	-1.14232300
C	3.76800900	2.28226900	-1.05796300
C	1.55814600	2.33822300	-2.04464500
C	4.15636000	3.34691300	-1.87403600
H	4.47598300	1.86387600	-0.34837700
C	1.95187900	3.39619200	-2.86453200
H	0.53636800	1.97019400	-2.08124600
C	3.25121700	3.90170400	-2.78110300
H	5.16449900	3.74538600	-1.79580400
H	1.23760300	3.83556600	-3.55537300
H	3.55392200	4.73318400	-3.41203200
C	2.99847800	0.36512500	1.35844400
C	4.18426200	-0.38227800	1.42726000
C	2.63324000	1.16381800	2.45654200
C	4.99017800	-0.32696400	2.56726200
H	4.48242700	-1.01200500	0.59478000
C	3.44538300	1.22365600	3.58875000
H	1.70505300	1.72700000	2.41978600
C	4.62478300	0.47706900	3.64780400

H	5.90383600	-0.91425500	2.60813500
H	3.15049700	1.84619000	4.42922900
H	5.25257900	0.51764600	4.53393700
C	-3.28595700	0.61950100	0.12236200
C	-3.56710300	2.08889700	-0.17114100
C	-3.28540000	3.06751800	0.79764600
C	-2.63241900	2.67593000	2.10964800
C	-2.63174100	1.14970200	2.33166400
H	-4.36492500	1.71158900	-2.13322300
H	-2.99468300	0.10886200	-0.80318300
C	-4.14727600	2.47440700	-1.38638200
C	-3.59491300	4.40652600	0.52267900
H	-1.59296400	3.03317300	2.10942800
H	-3.64858300	0.85772800	2.66496100
C	-4.17258600	4.78278300	-0.68920700
C	-4.44944300	3.80936400	-1.65281100
H	-3.37764100	5.16359400	1.27486600
H	-4.40558800	5.82749700	-0.88098800
H	-4.89590100	4.08957600	-2.60415700
N	-2.29806100	0.35028800	1.16067300
H	-0.34108500	1.91110400	0.62624300
H	-4.24882400	0.15537800	0.42473300
H	-1.94809900	0.90158200	3.15333200
H	-3.13863700	3.17959400	2.94692600

50-ts
Zero-point correction= 0.766073 (Hartree/Particle)
Thermal correction to Energy= 0.812246
Thermal correction to Enthalpy= 0.813190
Thermal correction to Gibbs Free Energy= 0.675025
E(Solv)=-2433.5813

C	-1.84096600	4.34138700	0.06624200
O	-2.99312100	3.65943200	-0.47934900
C	-2.66570700	2.34378100	-0.58371100
C	-0.70191900	3.29619300	-0.05462300
H	-1.67581800	5.24813000	-0.51766500
H	-2.06776000	4.60264900	1.10509000
H	-0.16310200	3.20146000	0.89140200
N	-1.44189300	2.03995800	-0.30856500
C	-3.76629000	1.49031000	-1.09475000
C	-4.80027900	2.18546700	-1.75481300
C	-3.83167400	0.07353800	-1.01162000
C	-5.86598600	1.51740600	-2.34726100
H	-4.74875500	3.26539600	-1.80983100
C	-4.91916400	-0.57293000	-1.62032100
C	-5.92494600	0.12728100	-2.28466300
H	-6.64020400	2.08310600	-2.85743100
H	-4.97945800	-1.65487200	-1.57263800
H	-6.74739500	-0.41361900	-2.74453500
P	-2.58058000	-0.96574300	-0.10303300
Pd	-0.52582500	0.09849400	0.21056600
C	0.29903600	3.61624700	-1.15686300
C	0.16240200	3.12144300	-2.45882600
C	1.36275900	4.48324800	-0.86946600
C	1.07206600	3.48718700	-3.45387900
H	-0.64513100	2.43435000	-2.69157000
C	2.26792600	4.85360600	-1.86433700
H	1.48972800	4.85849900	0.14334600
C	2.12516500	4.35486100	-3.16126300
H	0.95759600	3.08920300	-4.45867500
H	3.08959900	5.52312200	-1.62437800
H	2.83383100	4.63554200	-3.93551100
C	-2.63191100	-2.55375000	-1.04257500
C	-3.41473200	-3.65601700	-0.66701500
C	-1.81415900	-2.65777900	-2.18038900
C	-3.38670000	-4.83241000	-1.42029300
H	-4.04362700	-3.60022200	0.21639600
C	-1.79775900	-3.82836700	-2.93809300
H	-1.17858700	-1.82169400	-2.46162900
C	-2.58344100	-4.91979400	-2.55820900
H	-3.99406500	-5.68039500	-1.11493400
H	-1.16020700	-3.89370800	-3.81560300
H	-2.56137200	-5.83642300	-3.14123600
C	-3.44054200	-1.35904200	1.48608400
C	-4.73571800	-0.93164300	1.81212700
C	-2.71170300	-2.09551000	2.43837900
C	-5.28971900	-1.23611100	3.05911200
H	-5.31906500	-0.36040600	1.09754600
C	-3.27286900	-2.41212400	3.67400200
H	-1.70029500	-2.41895700	2.20611700
C	-4.56385200	-1.97848200	3.99017800
H	-6.29297600	-0.89282800	3.29776100
H	-2.69843400	-2.98855000	4.39424600
H	-4.99749500	-2.21550200	4.95789400
C	1.50950300	0.48499200	0.90363900
C	1.21995100	-0.90600400	1.06022500
H	2.08545700	0.76222500	0.02026300
H	1.02573800	-1.30469000	2.05540600

C	1.34928500	-1.83747900	0.00498000
C	1.20105800	-3.27756600	0.14014000
H	1.65634900	-1.45507900	-0.96466500
H	0.71452700	-3.59701500	1.06752000
C	1.65780300	1.41107100	2.04085100
C	2.53666500	2.50817400	1.93455900
C	0.95483200	1.25450500	3.25501800
C	2.70928500	3.40419800	2.99007300
H	3.09659900	2.64544300	1.01301100
C	1.13086900	2.14756300	4.30922700
H	0.25298200	0.43203400	3.36335000
C	2.00793800	3.23014000	4.18499000
H	3.40315900	4.23438900	2.88136800
H	0.57721400	2.00034500	5.23337800
H	2.14344400	3.92498800	5.00933200
H	0.74647900	-3.76174900	-0.72997700
C	4.90521900	-3.16203600	-0.95674700
C	6.05829000	-2.15528600	-0.95149600
C	6.29665300	-1.37222500	0.19117400
C	5.38749100	-1.49779200	1.39878700
C	4.54023400	-2.78948600	1.33341300
H	6.70945100	-2.63033300	-2.94616300
H	5.36725300	-4.17151400	-0.84109400
C	6.89858900	-2.02051000	-2.06350800
C	7.37531000	-0.47655500	0.18918100
H	5.98205900	-1.46689700	2.32391200
H	3.77597800	-2.75932500	2.12452800
C	8.20679300	-0.34613100	-0.92244900
C	7.96591400	-1.12311600	-2.05867200
H	7.56286700	0.12506100	1.07740700
H	9.03828600	0.35450900	-0.90240700
H	8.60516600	-1.02942400	-2.93367400
N	3.89923100	-2.92188700	0.04782000
H	2.34064000	-3.57299900	0.13694500
H	4.43739000	-3.16381700	-1.95311900
H	5.20988100	-3.64793900	1.56616700
H	4.70633700	-0.63576800	1.43456900

51

Zero-point correction= 0.697609 (Hartree/Particle)

Thermal correction to Energy= 0.742485

Thermal correction to Enthalpy= 0.743429

Thermal correction to Gibbs Free Energy= 0.618208

E(Solv) = -3085.09196565

Pd	0.52161700	-2.13492000	0.07650300
P	-1.53101000	-0.98191300	-0.28361900
P	1.90261500	-0.22350400	0.04315700
C	-2.53372600	-2.00100000	-1.46810400
C	-3.83963200	-2.42842800	-1.19203400
C	-4.51770100	-3.26699200	-2.08319300
C	-3.90734400	-3.68308300	-3.26508900
C	-2.60390000	-3.26495700	-3.55109300
C	-1.92135400	-2.44335400	-2.65605500
C	-2.69187700	-0.64207700	1.10197200
C	-3.83534800	0.15477400	0.90990500
C	-4.73146000	0.36049900	1.95699800
C	-4.48996100	-0.21820000	3.20725700
C	-3.35038700	-0.99673400	3.40502200
C	-2.44803900	-1.21326400	2.35851700
C	2.20619600	0.56296400	-1.59475500
C	2.63137600	1.89385500	-1.72807700
C	2.92704700	2.42056400	-2.98574300
C	2.80732100	1.62275900	-4.12598000
C	2.38564300	0.29764800	-4.00482300
C	2.08428200	-0.22754400	-2.74688900
C	3.61757200	-0.48281900	0.69952200
C	3.79096200	-1.33925000	1.80279600
C	5.06863900	-1.57953300	2.31117600
C	6.18872900	-0.98853500	1.72316300
C	6.02490900	-0.14936800	0.62065700
C	4.74953600	0.10433100	0.11120600
H	-4.33264000	-2.11550800	-0.27859600
H	-5.52732400	-3.59215300	-1.84654800
H	-4.43700700	-4.33287200	-3.95639000
H	-2.11542700	-3.58735200	-4.46700300
H	-0.90044300	-2.14131300	-2.87558400
H	-4.02674200	0.61112000	-0.05793200
H	-5.61383100	0.97563800	1.80016400
H	-5.18745500	-0.05306200	4.02457000
H	-3.14851100	-1.43722500	4.37744300
H	-1.54555200	-1.79782000	2.53089400
H	2.72594400	2.52283200	-0.84817100
H	3.24282000	3.45627600	-3.07413600
H	3.03774200	2.03455300	-5.10490900
H	2.28746900	-0.32705600	-4.88879300
H	1.74558100	-1.25585500	-2.64931600
H	2.92472200	-1.81113500	2.26582700
H	5.18230500	-2.23860300	3.16775600

H	7.18251400	-1.18414900	2.11778300
H	6.88969700	0.31130600	0.14998800
H	4.64882200	0.75383100	-0.75054400
C	2.08296000	-3.70853900	-0.15907000
H	2.25451000	-3.90146400	0.89580500
C	0.89314200	-4.15147700	-0.77428000
C	-0.28922600	-4.21360900	-0.00922500
H	0.83134200	-4.19414300	-1.86064100
C	-1.37439400	0.66755800	-1.14197800
C	-1.96722600	0.83591800	-2.42650800
C	-0.65966700	1.72601200	-0.57197900
C	-1.83903100	2.00453200	-3.13387800
C	-0.51329800	2.95861800	-1.30801500
C	-1.10312800	3.09191400	-2.60642100
C	1.11262800	1.04578600	1.14695300
C	1.65205500	1.18221400	2.45640700
C	-0.08424600	1.69431800	0.82107500
C	1.02494500	1.93438900	3.41632100
C	-0.76679800	2.46560700	1.82996600
C	-0.20060500	2.58485200	3.14097700
H	-2.30833800	2.10632200	-4.10967600
H	-2.54221500	0.02601800	-2.85687500
H	2.57484600	0.67568700	2.70686500
H	1.46156900	2.02437300	4.40776900
C	-0.86893500	3.35455700	4.13121000
C	-2.00219200	3.13190800	1.58158000
H	-0.41934800	3.42915400	5.11838300
C	-2.05740500	3.98677900	3.85190500
H	-2.56143600	4.57204600	4.61618900
C	-2.62821200	3.86772200	2.56237100
H	-3.57248100	4.35957200	2.34473500
H	-2.46137400	3.04919400	0.60396000
C	0.21247600	4.07469100	-0.79748200
C	-0.95076200	4.30398800	-3.33243000
C	0.34589400	5.23669800	-1.52478600
H	-1.40944700	4.37989400	-4.31549600
C	-0.24127000	5.35821000	-2.80683900
H	0.66784700	4.00620300	0.18311700
H	0.90462200	6.07027600	-1.10771200
H	-0.13179200	6.28187400	-3.36857900
Cl	0.72329900	-2.62561200	2.85267400
H	2.95887200	-3.49923800	-0.76688900
H	-1.23799800	-4.39054500	-0.50637600
H	-0.24154300	-4.41317200	1.05786400

52-ts

Zero-point correction= 0.768958 (Hartree/Particle)
Thermal correction to Energy= 0.817946
Thermal correction to Enthalpy= 0.818890
Thermal correction to Gibbs Free Energy= 0.681021
E(Solv) = -3311.36988456

Pd	-1.03644900	0.62914600	-1.20243700
P	0.61369800	1.93673000	-0.11688400
P	0.10163200	-1.44181900	-0.98584900
C	0.75425800	3.59934100	-0.92071700
C	0.85256100	4.80394100	-0.20997700
C	0.91291700	6.02515700	-0.88737100
C	0.88377000	6.06160500	-2.28128800
C	0.78008900	4.86801000	-3.00085600
C	0.70392400	3.65061300	-2.32554700
C	0.28598500	2.27528700	1.66018400
C	1.25293100	2.86822800	2.49283200
C	0.96577000	3.11688800	3.83411000
C	-0.28044200	2.76181300	4.36284600
C	-1.23492600	2.15277900	3.54931500
C	-0.95457500	1.91114100	2.20159200
C	1.63221100	-1.72127200	-1.98464200
C	2.49159000	-2.80862700	-1.76419800
C	3.58317900	-3.02837200	-2.60257400
C	3.82601000	-2.17051700	-3.67880600
C	2.97335400	-1.09134700	-3.91202700
C	1.88201200	-0.86898400	-3.06925200
C	-0.75410400	-3.06170900	-1.26925500
C	-1.63303300	-3.13509600	-2.36086400
C	-2.24962300	-4.33923400	-2.70407500
C	-2.00313700	-5.48933500	-1.95272600
C	-1.12926100	-5.42965200	-0.86552900
C	-0.50351200	-4.22805300	-0.52928900
H	0.87008500	4.79623000	0.87419600
H	0.98095600	6.94898800	-0.31899600
H	0.93111600	7.01266500	-2.80482100
H	0.74384000	4.88565800	-4.08697600
H	0.59128900	2.72801900	-2.88999800
H	2.23257000	3.12331400	2.09648100
H	1.71746200	3.57877700	4.46949600
H	-0.49616800	2.94808400	5.41183100
H	-2.19664200	1.83569600	3.94130700
H	-1.70520800	1.40257700	1.60378200

H	2.30836300	-3.48896100	-0.93788000
H	4.24541800	-3.86930100	-2.41569000
H	4.67600100	-2.34574200	-4.33290500
H	3.15483500	-0.42195400	-4.74883400
H	1.21517700	-0.02934700	-3.24825600
H	-1.82937100	-2.24121000	-2.94731700
H	-2.92707100	-4.37600900	-3.55311400
H	-2.49033800	-6.42570600	-2.21068000
H	-0.93211500	-6.32048900	-0.27498500
H	0.16892900	-4.19926100	0.32204900
C	-2.59001500	2.07832500	-1.38944800
H	-2.95137700	2.28571700	-0.38352800
C	-3.05641900	0.91430000	-2.06555100
H	-3.04684600	0.88460800	-3.15387700
C	-3.60744700	-0.19089800	-1.36581000
C	2.35650100	1.27103500	-0.14090400
C	2.68353000	0.05117700	0.46656500
C	3.36064400	1.99488800	-0.84214000
C	4.03110000	-0.44515000	0.37890900
C	4.64943300	1.53007400	-0.93610600
H	3.10831800	2.94207600	-1.30396000
C	5.02253800	0.30410000	-0.33573200
C	0.56083700	-1.42644600	0.80410900
C	-0.36794800	-1.93151600	1.70216200
C	1.69806700	-0.70584500	1.31355000
C	-0.19657700	-1.84216700	3.10594100
C	1.89583200	-0.66005000	2.68344900
C	0.98851900	-1.22713200	3.61139900
H	2.76605200	-0.13437400	3.06828900
H	-3.53638000	-0.23960300	-0.28018300
H	-1.28101500	-2.39433100	1.34197300
C	1.19242100	-1.16937000	5.01599900
C	-1.16457000	-2.34210000	4.01828800
H	2.09767000	-0.70365600	5.39822600
C	0.25011200	-1.68563800	5.87722700
H	0.41029000	-1.63604200	6.95112100
C	-0.94199500	-2.26687900	5.37474500
H	-1.68830300	-2.64350700	6.06860600
H	-2.09026400	-2.75265800	3.62647500
H	5.40070000	2.10754100	-1.46994800
C	4.43149200	-1.68076900	0.96610000
C	6.35185600	-0.19067800	-0.42283900
H	3.69759900	-2.26938000	1.50393300
H	7.08880200	0.39671400	-0.96558500
C	5.72726800	-2.13569800	0.86265900
C	6.70241200	-1.38392000	0.16435600
H	6.00402000	-3.08146200	1.32102000
H	7.72224700	-1.75226600	0.09306600
C	-7.44889400	0.34371600	0.03252000
C	-7.83598000	0.41645300	-1.30132500
C	-6.65435500	0.18947200	-2.02579000
N	-5.63338800	-0.01166000	-1.18556200
H	-8.01030100	0.45276000	0.94929100
H	-8.82610600	0.60474000	-1.68975600
H	-6.49355100	0.16141600	-3.09515500
N	-6.12747400	0.08834600	0.05901800
H	-5.46541800	-0.01758200	0.87961300
Cl	-3.96020000	-0.21538800	2.16311000
H	-2.32927900	2.95868100	-1.97217000
H	-3.69767900	-1.14232900	-1.87545700

53

Zero-point correction= 0.769109 (Hartree/Particle)

Thermal correction to Energy= 0.818739

Thermal correction to Enthalpy= 0.819684

Thermal correction to Gibbs Free Energy= 0.679806

E(Solv) = -3311.38810101

Pd	-1.15227100	0.21688300	-1.05650700
P	0.36311700	1.91818400	-0.35139800
P	0.27784900	-1.63855600	-0.74864000
C	0.28167200	3.40183300	-1.46061000
C	0.41746400	4.72264700	-1.00814000
C	0.31964000	5.79271600	-1.90111300
C	0.09220000	5.56004600	-3.25824000
C	-0.04815400	4.24916200	-3.72037500
C	0.03557500	3.18135000	-2.82670500
C	0.09818500	2.61864000	1.33387400
C	1.11303800	3.28069100	2.04685000
C	0.85061700	3.82306100	3.30525300
C	-0.42543400	3.71021000	3.86664500
C	-1.43721000	3.05137700	3.16781200
C	-1.17577000	2.50518700	1.90897600
C	1.75824100	-1.81616900	-1.84656000
C	2.85554000	-2.63610800	-1.54302200
C	3.89661100	-2.79418800	-2.45665900
C	3.85195800	-2.14210200	-3.69181200
C	2.76473200	-1.32642200	-4.00604900
C	1.72545900	-1.16318200	-3.08721600
C	-0.36783600	-3.38144200	-0.82279800

C	-1.46916500	-3.61208100	-1.66061400
C	-1.96803700	-4.90312300	-1.85218100
C	-1.37871200	-5.98425600	-1.19652000
C	-0.28287300	-5.76848200	-0.35685800
C	0.22281200	-4.48066400	-0.17726500
H	0.59052600	4.92049200	0.04461000
H	0.41928900	6.81008500	-1.53168000
H	0.01522500	6.39452800	-3.95028300
H	-0.23719800	4.05834000	-4.77369700
H	-0.10474600	2.16359700	-3.18292700
H	2.10932700	3.36739100	1.62114800
H	1.64290000	4.33338300	3.84781300
H	-0.62549800	4.13230000	4.84829700
H	-2.43498200	2.94570600	3.58337700
H	-1.97600600	1.98725200	1.39054000
H	2.90669400	-3.14668700	-0.58623100
H	4.74567700	-3.42281900	-2.20222800
H	4.66369400	-2.26834000	-4.40342200
H	2.72442500	-0.81382800	-4.96369500
H	0.87942400	-0.52336300	-3.32585200
H	-1.92600900	-2.76632300	-2.16807400
H	-2.81707800	-5.06294100	-2.51222400
H	-1.76837800	-6.98890700	-1.33747800
H	0.18290600	-6.60572200	0.15657400
H	1.07357700	-4.33283500	0.48075900
C	-3.00274300	1.23818100	-1.42465600
H	-3.36255500	1.73792200	-0.52647800
C	-3.22208100	-0.14960600	-1.59698900
H	-3.29304000	-0.55408800	-2.60789700
C	-3.95325500	-0.94785000	-0.54368700
C	2.17525800	1.47385300	-0.36185100
C	2.66419900	0.39070100	0.37754200
C	3.06626500	2.20951100	-1.19339700
C	4.06132300	0.04813900	0.29855600
C	4.39753700	1.88818200	-1.28696900
H	2.68628600	3.05062700	-1.76103200
C	4.93363300	0.80496200	-0.55083900
C	0.88007000	-1.42179900	0.98914500
C	0.20178300	-2.08752200	1.99891900
C	1.81892500	-0.38819900	1.35207800
C	0.40640700	-1.81322400	3.37456700
C	2.02300000	-0.11706700	2.69381600
C	1.34261600	-0.79755000	3.73378500
H	2.73761800	0.65493800	2.96661600
H	-3.73772200	-0.58065200	0.46231400
H	-0.53341800	-2.84517500	1.74601800
C	1.55797900	-0.50928200	5.10765200
C	-0.28808700	-2.50700700	4.40210300
H	2.26896400	0.26823100	5.37604100
C	0.87221800	-1.19931800	6.08243500
H	1.03944700	-0.97064300	7.13144600
C	-0.05979200	-2.20658700	5.72635000
H	-0.59778300	-2.73899900	6.50590800
H	-1.00595900	-3.27542800	4.12524400
H	5.05725200	2.47085800	-1.92594000
C	4.63046200	-1.03878400	1.02537100
C	6.31013000	0.46197400	-0.63451100
H	3.99490400	-1.63022700	1.67375000
H	6.95217100	1.05384200	-1.28281200
C	5.96844900	-1.34921800	0.92163500
C	6.82279900	-0.59181400	0.08524800
H	6.37256600	-2.18339300	1.48928800
H	7.87738500	-0.84438800	0.01504900
C	-7.42164300	0.00403500	-0.39580000
C	-7.55269300	-1.00664600	-1.35286300
C	-6.27045800	-1.52635900	-1.50613100
N	-5.44137900	-0.85918100	-0.68055500
H	-8.15333500	0.66591400	0.04419400
H	-8.45186500	-1.32296800	-1.85917800
H	-5.89402400	-2.31629800	-2.13955700
N	-6.14204000	0.07693700	-0.01657700
H	-5.64113000	0.69999900	0.79857400
Cl	-4.81439000	1.55383900	2.06647300
H	-2.89644700	1.88082600	-2.29695100
H	-3.72165300	-2.01525200	-0.58922900

54

Zero-point correction= 0.697245 (Hartree/Particle)

Thermal correction to Energy= 0.742266

Thermal correction to Enthalpy= 0.743210

Thermal correction to Gibbs Free Energy= 0.613327

E(Solv) = -3085.06730844

Pd	1.80179200	0.89040600	-0.33438800
P	1.11880400	-1.37945200	-0.20282900
P	-0.31829600	1.88011300	0.07287200
C	2.22791400	-2.60199800	-1.05920000
C	2.38277100	-3.94043000	-0.66686800
C	3.25893300	-4.78593000	-1.35112900

C	3.98878400	-4.31227600	-2.44247200
C	3.84518300	-2.98235400	-2.84309000
C	2.98015600	-2.13413200	-2.15067400
C	0.91447800	-2.12269400	1.47176200
C	0.13117500	-3.25798500	1.73151000
C	0.07432200	-3.80229900	3.01508400
C	0.80079000	-3.22054200	4.05647100
C	1.57868300	-2.08766300	3.81178300
C	1.63153400	-1.54041700	2.52862700
C	-1.32248300	2.33244000	-1.40565000
C	-2.70144500	2.58982700	-1.35322300
C	-3.39326400	2.98182400	-2.49992200
C	-2.71695900	3.11954800	-3.71417500
C	-1.34718200	2.85901000	-3.77940000
C	-0.65470200	2.46514800	-2.63302000
C	-0.18689500	3.48267500	1.00126800
C	0.83441900	3.58864900	1.96206700
C	1.01147000	4.76237400	2.69427800
C	0.18273400	5.86289600	2.46223800
C	-0.82353900	5.77674600	1.49963300
C	-1.01106300	4.59536000	0.77730700
H	1.83195300	-4.32740400	0.18341800
H	3.37117500	-5.81726700	-1.02667100
H	4.67181200	-4.97182900	-2.97106700
H	4.41681700	-2.59943300	-3.68431200
H	2.88881300	-1.09312300	-2.44779500
H	-0.44580500	-3.71235000	0.93173600
H	-0.54532200	-4.67487500	3.20266500
H	0.75467700	-3.64528400	5.05573700
H	2.14079600	-1.62585300	4.61918600
H	2.22670600	-0.65101600	2.33776000
H	-3.23729300	2.47888400	-0.41489100
H	-4.46182600	3.17199600	-2.44624200
H	-3.25791400	3.42233600	-4.60680500
H	-0.81681100	2.95705100	-4.72292100
H	0.40976100	2.25102700	-2.68247000
H	1.49865700	2.74381200	2.12523800
H	1.80545000	4.82196900	3.43402800
H	0.32699300	6.78325800	3.02179000
H	-1.46731800	6.63090600	1.30602800
H	-1.79683300	4.55033000	0.03092300
C	-0.51830100	-1.57395700	-1.08176000
C	-0.48985100	-2.17397100	-2.37453300
C	-1.71049900	-0.99566200	-0.62366100
C	-1.59347600	-2.19918300	-3.18857400
C	-2.88016000	-1.01963300	-1.46962300
C	-2.81526800	-1.62283100	-2.76773100
C	-1.44689200	0.84703400	1.14473900
C	-1.74098500	1.32090400	2.45639700
C	-1.88672800	-0.42790200	0.76412900
C	-2.43788800	0.56264000	3.36263800
C	-2.62986300	-1.23135800	1.70506500
C	-2.89788900	-0.73095000	3.02054400
H	-1.53996300	-2.67165900	-4.16672600
H	0.42881400	-2.62777100	-2.72569900
H	-1.40862200	2.30962700	2.74765100
H	-2.65034400	0.95491200	4.35454500
C	-3.62518300	-1.52618700	3.94699300
C	-3.12442900	-2.53006800	1.38455800
H	-3.81333700	-1.12238600	4.93905500
C	-4.08487800	-2.77478200	3.59997300
H	-4.64320200	-3.37379400	4.31423700
C	-3.82925700	-3.27734300	2.30191200
H	-4.19472800	-4.26267300	2.02478300
H	-2.94295400	-2.93637000	0.39682300
C	-4.13106400	-0.46671400	-1.06629900
C	-3.96935400	-1.64844600	-3.59646100
C	-5.23442700	-0.50911500	-1.88927300
H	-3.89153900	-2.11194800	-4.57714600
C	-5.15848600	-1.10535300	-3.17022900
H	-4.21496800	-0.00557200	-0.08976000
H	-6.17383200	-0.08023200	-1.55044400
H	-6.03662100	-1.13295700	-3.80961000
Cl	6.56783600	1.22959900	0.08907500
C	3.90597200	1.22853600	-0.79613300
C	3.23374400	2.46294400	-0.66262400
C	4.78142900	0.67080700	0.27069600
H	4.12648600	0.86299500	-1.79846400
H	3.35573800	3.05627100	0.24278800
H	2.97871400	3.04287300	-1.54750800
H	4.85141500	-0.41609000	0.24462800
H	4.49871500	1.00674700	1.26873900
